# Structural Basis for Plexin Activation and Regulation

**DOI:** 10.1016/j.neuron.2016.06.018

**Published:** 2016-08-03

**Authors:** Youxin Kong, Bert J.C. Janssen, Tomas Malinauskas, Vamshidhar R. Vangoor, Charlotte H. Coles, Rainer Kaufmann, Tao Ni, Robert J.C. Gilbert, Sergi Padilla-Parra, R. Jeroen Pasterkamp, E. Yvonne Jones

**Affiliations:** 1 Division of Structural Biology, Wellcome Trust Centre for Human Genetics, University of Oxford, Oxford, United Kingdom; 2 Department of Translational Neuroscience, Brain Center Rudolf Magnus, University Medical Center Utrecht, Utrecht, The Netherlands; 3 Department of Biochemistry, University of Oxford, Oxford, United Kingdom

**Keywords:** semaphorin signaling, axon guidance, autoinhibition, structure-function

## Abstract

Class A plexins (PlxnAs) act as semaphorin receptors and control diverse aspects of nervous system development and plasticity, ranging from axon guidance and neuron migration to synaptic organization. PlxnA signaling requires cytoplasmic domain dimerization, but extracellular regulation and activation mechanisms remain unclear. Here we present crystal structures of PlxnA (PlxnA1, PlxnA2, and PlxnA4) full ectodomains. Domains 1–9 form a ring-like conformation from which the C-terminal domain 10 points away. All our PlxnA ectodomain structures show autoinhibitory, intermolecular “head-to-stalk” (domain 1 to domain 4-5) interactions, which are confirmed by biophysical assays, live cell fluorescence microscopy, and cell-based and neuronal growth cone collapse assays. This work reveals a 2-fold role of the PlxnA ectodomains: imposing a pre-signaling autoinhibitory separation for the cytoplasmic domains via intermolecular head-to-stalk interactions and supporting dimerization-based PlxnA activation upon ligand binding. More generally, our data identify a novel molecular mechanism for preventing premature activation of axon guidance receptors.

## Highlights

• Structural studies reveal a major ring-like conformation for PlxnA ectodomains
• PlxnA ectodomains make head-to-stalk *cis*-interactions in vitro and on cell surface
• Disruption of PlxnA *cis*-interactions induces cell and growth cone collapse
• PlxnA ectodomain structure and interaction enable autoinhibition and activation

## Teaser

PlxnA signaling, important in nervous system development and plasticity, requires multi-leveled regulation. Kong et al. reveal a novel mechanism for PlxnAs, in which autoinhibition pre- and activation post-ligand binding are achieved through distinct conformations and *cis*-interactions of the receptor ectodomains.

## Introduction

The guidance of axons to their synaptic targets is an important event during neural circuit development. Axon guidance cues are also implicated in processes such as neuronal migration and polarization, neurite growth and pruning, and synaptogenesis (Kolodkin and Tessier-Lavigne, 2011, Bashaw and Klein, 2010, Yogev and Shen, 2014). Cell surface receptors at the leading tips of growing neurites or migrating cells function to detect axon guidance cues and to elicit a cellular response. Different families of guidance receptors have been identified, but our understanding of how these receptors signal or how their activity is regulated is very incomplete. For example, receptor pre-clustering prior to ligand binding may be important for the spatial and temporal regulation of signaling. However, the molecular mechanisms by which receptor activation is achieved and ligand-independent activation avoided are for many systems still obscure (Atanasova and Whitty, 2012).

**Figure 1:**
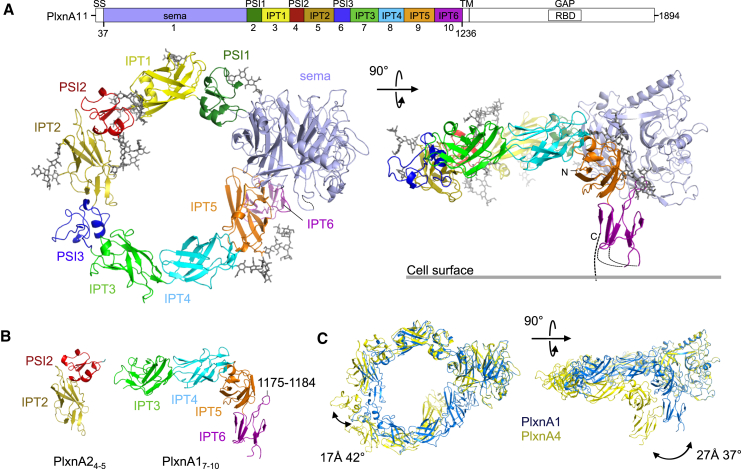
Crystal Structures of PlxnAs Extracellular Segments (A) Schematic domain organization of PlxnA1 (top). SS, signal sequence; sema, semaphorin; PSI, plexin-semaphorin-integrin; IPT, Ig domain shared by plexins and transcription factors; TM, transmembrane region; GAP, GTPase activating protein; RBD, Rho GTPase-binding domain. The domains included in the crystallization constructs are colored. Ribbon representation of PlxnA1_1-10_ at 4 Å resolution in top and side views (left and right, respectively). A gray line indicates how PlxnA1 is attached to the cell surface (right). N-linked glycans are shown in gray stick representation. (B) Ribbon representation of PlxnA2_4-5_ at 1.36 Å resolution (left) and of PlxnA1_7-10_ at 2.2 Å resolution (right). Loop 1,175–1,184 involved in contacts with domain IPT5 is indicated. (C) Ribbon representation of PlxnA1_1-10_ and PlxnA4_1-10_ superposed on the basis of the sema domain (views as in [A]). Differences in PSI3 and IPT6 domain orientation (left and right, respectively) are indicated by domain translation and rotation. See also Figure S1 and Table S1.

Semaphorins form the largest family of secreted and membrane-anchored axon guidance cues and generally require plexins as cell surface receptors in neurons (Kolodkin and Tessier-Lavigne, 2011, Pasterkamp, 2012, Jongbloets and Pasterkamp, 2014). Plexins are large type 1 transmembrane proteins comprising typically ten domains in their extracellular segment, a single membrane spanning region that is predicted to be helical and a conserved, cytoplasmic region that contains a GTPase-activating protein (GAP) domain with a Rho GTPase-binding domain insert (Figure 1A). Plexins are subdivided into four classes (A to D) based on sequence similarity. Class A plexins (PlxnAs) are the best-characterized and act as neuronal receptors for secreted class 3 (Sema3s), as well as transmembrane class 5 and class 6 (Sema5s and Sema6s) semaphorins (Pasterkamp, 2012).

The largest portion of plexin ectodomain structurally characterized to date, PlxnA2_1-4_, comprises the ligand-binding sema domain, followed by a PSI, IPT, and a second PSI domain (Janssen et al., 2010). The mechanism of semaphorin–PlxnA recognition has been uncovered by crystal structures of PlxnA ectodomain fragments in complex with their cognate semaphorins (Janssen et al., 2010, Liu et al., 2010, Nogi et al., 2010). In isolation, the semaphorin ligand is characteristically a dimer (Siebold and Jones, 2013). In semaphorin–plexin structures each subunit of a semaphorin dimer binds a monomeric plexin sema domain (Janssen et al., 2010, Janssen et al., 2012, Liu et al., 2010, Nogi et al., 2010). These 2:2 semaphorin–plexin complexes indicate a bivalent binding mode is required to activate plexins, a mechanism supported by in solution and cell-based assays (Janssen et al., 2010, Nogi et al., 2010). Sema3s require neuropilins to cross-brace the Sema3–PlxnA complex, but the generic, 2:2 plexin–semaphorin interaction, mediated by the sema domains of both binding partners, is conserved (Janssen et al., 2012). Dimerization of the plexin cytoplasmic region is a pre-requisite for activation (Wang et al., 2012) and crystal structures of engineered cytoplasmic region dimers support a model in which the bivalent binding of the semaphorin ligand to two plexin ectodomains drives the juxtaposition of their cytoplasmic regions with consequent dimerization and activation (Wang et al., 2013). Interestingly, functional and fluorescent light microscopy analyses have also suggested that the PlxnA ectodomain plays an essential role in autoinhibition of signaling prior to semaphorin binding (Takahashi and Strittmatter, 2001, Marita et al., 2015). To establish how semaphorin binding is coupled to cytoplasmic domain dimerization and how the PlxnA ectodomain provides autoinhibition pre-ligand binding, structural data are required on the full extracellular PlxnA segment.

In this study, we first detail a distinctive ring-like architecture that is common to the full-length (ten domain) extracellular segments of PlxnA1, PlxnA2, and PlxnA4. Negative stain electron microscopy (EM) confirms this ring-like structure as the predominant form (alongside a minor twisted-open, chair-like conformation). Our crystal structures also reveal an intriguing intermolecular “head-to-stalk” interaction common to all PlxnAs. We confirm this interface mediates PlxnA–PlxnA interactions and occurs pre-semaphorin binding using structure-guided mutagenesis, biophysical assays, and fluorescence microscopy-based live cell imaging. Functional assays (i.e., COS-7 cell-based and dentate gyrus [DG] granule cell growth cone collapse experiments) confirm that intermolecular *cis*-interactions of the PlxnA ectodomains impose pre-signaling autoinhibition. Based on these results, we propose that, before signaling, PlxnAs exist as pre-formed dimers in which the association between the ring-like PlxnA ectodomains maintains a separation of their cytoplasmic domains. This “outside-together-inside-apart” mechanism keeps PlxnA signaling in check in the absence of ligands while, possibly, allowing co-localization of receptors to facilitate rapid signaling in response to ligand binding.

## Results

### Crystal Structures of Full Extracellular Segments of PlxnAs Reveal Ring-Like Conformation

We determined crystal structures of the full extracellular segments of mouse PlxnA1, PlxnA2, and PlxnA4 from eight crystal forms providing a total of 13 independent structures that range in maximum resolution from 4 to 10 Å (Figures 1 and S1; Table S1). The 4 Å dataset was obtained by dehydrating crystals of PlxnA1_1-10_ that diffracted otherwise at best to 6 Å resolution (see Experimental Procedures). Structure determination of the full extracellular segments was aided by the available structures of PlxnA2_1-4_ (Janssen et al., 2010) (PDB: 3OKT), PlxnA2_1-2_ (Nogi et al., 2010) (PDB: 3AL9), and additional higher resolution crystal structures that we determined of portions of PlxnA1 and PlxnA2; that is, PlxnA2_4-5_ to 1.36 Å resolution and PlxnA1_7-10_ to 2.2 Å resolution (Figure 1B). In five out of 13 full extracellular structures all ten domains are resolved whereas in the other eight structures some of the C-terminal domains were not modeled due to fragmentary or absent electron density. This indicates that some flexibility is present in the extracellular segment of plexins.

In the crystal structure of PlxnA1-full ectodomain, the sema domain (domain 1, “head”) together with domains 2–9 (“stalk”) lie in a 230 Å long curve that forms a ring-like conformation (Figures 1A and S1A). All consecutive domains are arranged in a beads-on-a-string-like fashion, with short interdomain linkers (up to two residues) and extensive interfaces (buried surface area ranging from 716 to 1,172 Å^2^), each interdomain interface having a small hydrophobic core. These substantial domain–domain interactions likely contribute to conservation of the overall curved ring-like structure among the different PlxnA members and crystal forms. Some flexibility in the stalk is evident on comparison of the 13 different structures and contributes to the ring diameter ranging from 84 Å to 99 Å (Figures 1C and S1A). The interface between PSI3 and IPT3 (domains 6 and 7) differs markedly from those of PSI1-IPT1 and PSI2-IPT2, imposing a more acute angle on the relative arrangement of these two domains, and thus reducing the ring circumference (Figure S1B). The ring is closed only in the 4 Å structure of PlxnA1_1-10_, by interactions between the sema domain and domain IPT5 (domain 9). This relative compaction can, most likely, be attributed to the dehydration procedure applied to generate this crystal form. In all other structures, the plexin extracellular domains associate only via the consecutive domain-domain interactions. Thus, contrary to previous speculations on plexin ectodomain structure (Antipenko et al., 2003, Janssen et al., 2010, Takahashi and Strittmatter, 2001), in the unliganded state the sema domain does not make any intramolecular interactions with the remainder of the plexin other than to the directly ajoining PSI1 domain.

The membrane proximal domain IPT6 (domain 10) is positioned perpendicular to the ring formed by domains 1 to 9, and the IPT6 C terminus points away from the plane of the ring. This out-of-the-ring orientation of IPT6 is observed in all five PlxnA ectodomain structures for which the electron density is sufficiently well ordered to allow IPT6 to be resolved. The same relative domain orientation recurs in the structure of PlxnA1_7-10_. Possibly this distinctive orientation is stabilized by a loop (1,175–1,184) extending from IPT6 that provides hydrophobic interactions with three residues in domain IPT5, Trp1055, Ile1057, and Tyr1145 (Figures 1B and S1C), which are conserved across the A class Plxns. In the full-length plexin, the linker between IPT6 and the transmembrane helix is predicted to be short (6 residues for PlxnA1), and thus, for this conformation, the plexin extracellular segment is most likely positioned close to the membrane with its ring parallel to the cell surface.

### Negative Stain EM Supports a Major Ring-Like Conformation for PlxnA1_1-10_

**Figure 2:**
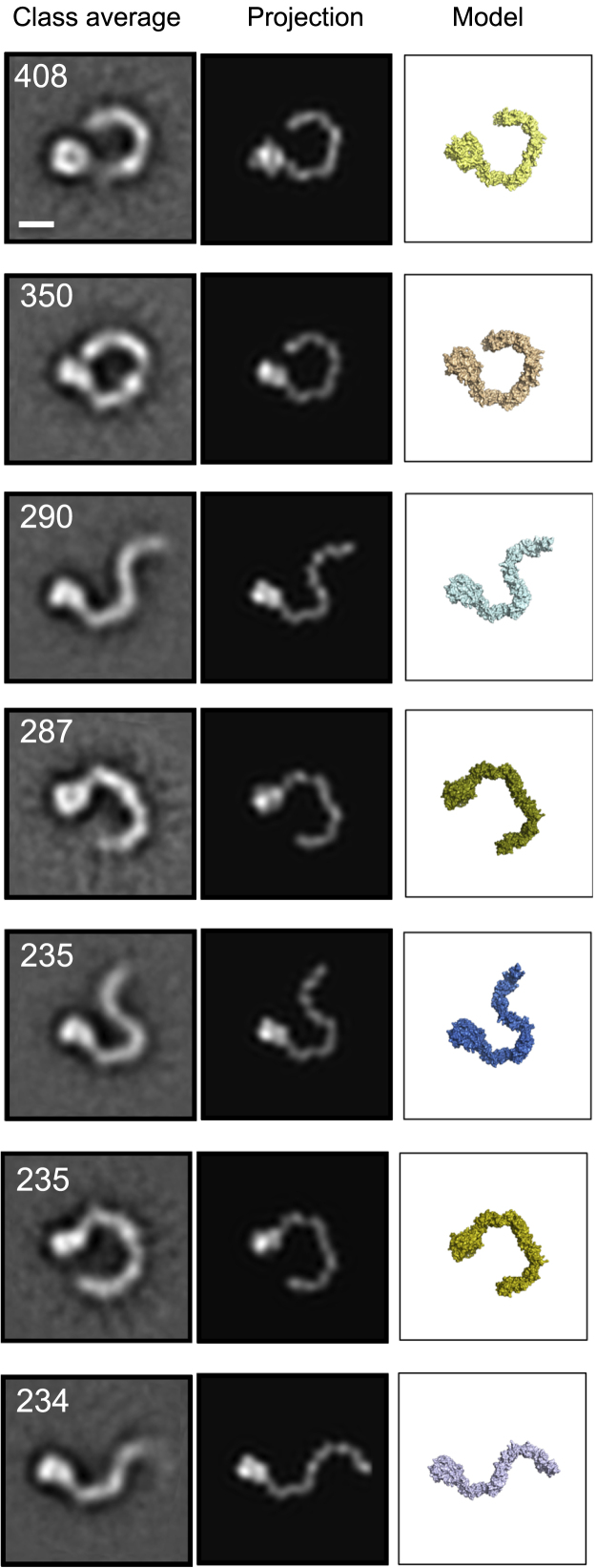
Negative Stain EM Reveals Two Alternative Conformations of PlxnA1_1-10_ Negative stain EM class averages of PlxnA1_1-10_ correlate well with 2D projections of the crystal-structure-based models. Seven representative class averages of PlxnA1_1-10_ are shown alongside the correlated projections and corresponding structural models (colored according to models shown in Figure S2B). The number of particles within each class is listed on the top left corner. The projections were generated from the 6 Å PlxnA1_1-10_ crystal structure of space group P2_1_ (see Figure S1A) filtered to a Gaussian resolution of 10 Å. Scale bar, 5 nm. See also Figure S2.

To assess the possible conformations of PlxnA full extracellular segments when removed from the constraints of crystal packing, we analyzed the structure of PlxnA1_1-10_ with negative stain EM (Figures 2 and S2). In the electron micrographs, PlxnA1_1-10_ is monomeric (Figure S2A). Assessed by eye, the majority of PlxnA1_1-10_ molecules appeared to orient with the flat face of the ring lying on the grid in a conformation resembling the crystal structures, though a proportion adopted a twisted-open conformation (Figure S2A). In agreement with this initial assessment, the class averages of PlxnA1_1-10_ sorted into two categories: “ring-like” or “chair-like” (twisted-open) conformations (Figures 2, S2B, and S2C). The ring-like species, which includes 66.0% of the particles, essentially matches the PlxnA_1-10_ crystal structures. A bulky feature at one end of the ring is readily identifiable as the sema domain (flat or sideways orientations on the grid resulting in a disk-like or bulb-like projection, respectively). From the sema domain onward, the stalk region extends in a range of curved conformations, resulting, as in the crystal structures, in some variation in ring circumference. A second species, which includes 30.5% of the particles, can be identified by its distinct chair-like conformation. The N-terminal half of the stalk region curves out from the sema domain as in the ring-like structures, but the C-terminal half appears twisted by a 180° flip so that it curves away rather than completing the ring. No clear class averages could be generated for the remaining 3.5% of the particles. The PlxnA_1-10_ crystal structures could be fitted to the class averages by assuming two flexion points, one between domains 1 and 2 (sema–PSI1) and another at the domain 5–domain 6 (IPT3–PSI3) junction (see Figure S2 and Supplemental Experimental Procedures). The excellent agreement between the class averages and projections of the structural models generated by rotations around these two potential flexion points reinforces the notion that the ten domain extracellular segments of PlxnAs have relatively limited flexibility (Figures 2 and S2C). Our results indicate that PlxnA ectodomains exist in only two alternative conformations with limited interdomain flexibility: the preferred, ring-like conformation as observed in crystal structures and the less frequent, chair-like conformation.

### PlxnA Crystal Structures Reveal an Intermolecular Interface between the Sema and PSI2-IPT2 Domains

**Figure 3:**
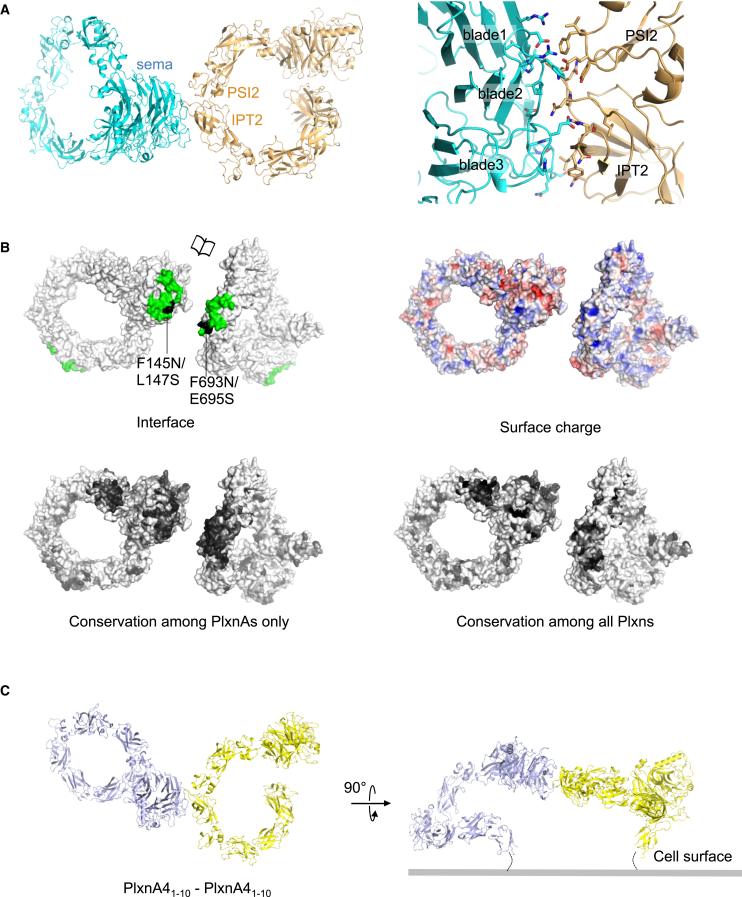
PlxnA Intermolecular Interface between the Sema Domain and Domains PSI2-IPT2 (A) Ribbon representation of the PlxnA1_1-10_ intermolecular interaction in the 4 Å crystal structure (left) and enlargement with interface residues shown in stick representation (right). (B) Surface representation of an opened view of two PlxnA1_1-10_ molecules with the sema–PSI2-IPT2 interface in green and interface mutants in black (top left) and electrostatic potential from red (−6 k_b_T/e_c_) to blue (6 k_b_T/e_c_) (top right). PlxnA1_1-10_ is color coded according to residue conservation (from nonconserved, white, to conserved, black) on the basis of alignments containing vertebrate PlxnA sequences only (bottom left) or Plxn sequences from all classes (bottom right). The structure of domain IPT6 has several unmodelled loops (see Experimental Procedures) and hence is revealing its conserved hydrophobic core in these views. See also Figure S3. (C) Possible cell surface orientation of PlxnA dimers formed via the sema–PSI2-IPT2 interface based on the conformation and packing of the PlxnA4_1-10_ crystal structure (space group P4_1_).

All our PlxnA full ectodomain crystal structures have an intermolecular plexin–plexin interface in common (Figures 3 and S3A). This interface is formed by blades 1–3 of the sema domain of one plexin molecule and the outer side of domains 4-5 (PSI2-IPT2) from the ring of a second plexin (Figure 3A). In the 4 Å PlxnA1_1-10_ structure, the interface has a buried surface area of 1,303 Å^2^ (355 Å^2^ between sema–PSI2 and 1,008 Å^2^ between sema–IPT2) and a complementary electrostatic charge distribution (Figure 3B). The residues in this interface are conserved among vertebrate PlxnAs but much less among all plexins (Figure 3B), suggesting that this plexin–plexin interaction may be an important functional feature of PlxnAs. This finding raises the possibility that PlxnA molecules on the same cell surface can interact in *cis* in a “head-to-stalk” manner using the sema–PSI2-IPT2 intermolecular interface to form dimers or small oligomeric clusters. A simple model-building exercise confirmed this possibility (Figures 3C and S3B). Taking into account the tilt of 50 degrees between the rings of two interacting PlxnAs, and the flexibility in the stalk region, PlxnA dimers can be accommodated on the cell surface (Figure 3C), but arrays incorporating more than three molecules clash with the plasma membrane in the absence of substantial membrane curvature (Figure S3B). Thus, our structural data raise the possibility that the ring-like PlxnA ectodomains exist as “head-to-stalk” dimers on the cell surface.

### In Solution Assays Confirm the Intermolecular Head-to-Stalk Interactions between PlxnA Ectodomains

**Figure 4:**
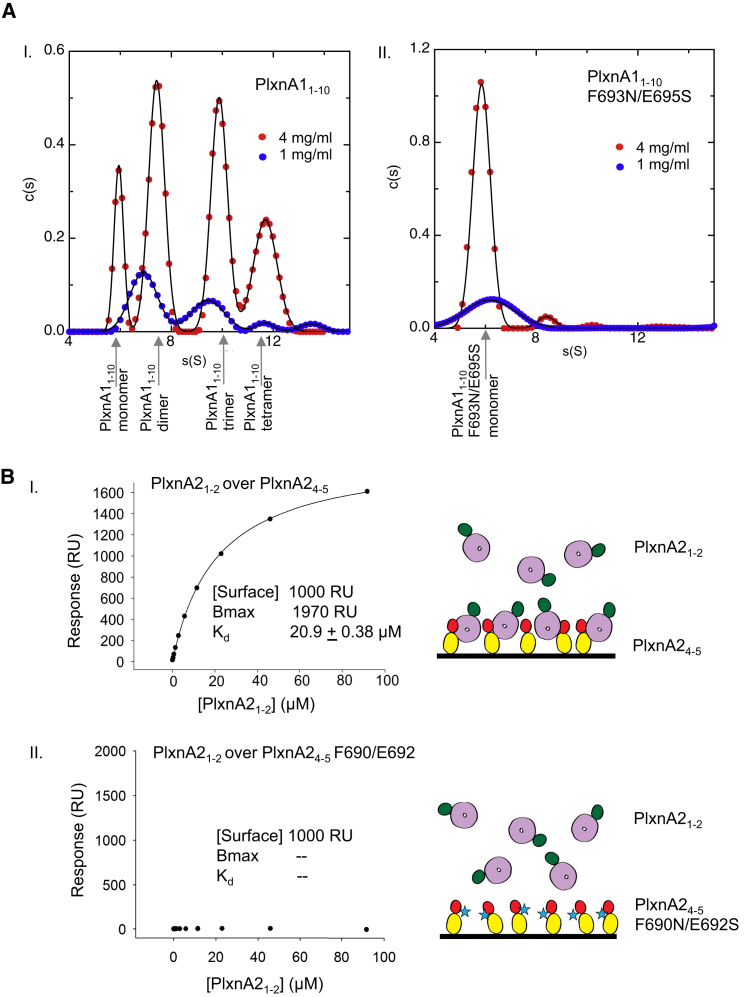
AUC and SPR Experiments Reveal PlxnA–PlxnA interactions via the Sema–PSI2-IPT2 Interface (A) Distribution of sedimentation coefficients of PlxnA1_1-10_ (I) and PlxnA1_1-10_ F693N/E695S (II) at different concentrations measured by AUC sedimentation velocity experiments. Calculated from the Lamm equation model, c(s) (in a.u.) is plotted against the sedimentation coefficient, S (in svedbergs). The predicted sedimentation coefficients of different oligomeric states that best correspond to the peak values are indicated by arrows. For PlxnA1_1-10_ F693N/E695S 28.1 μM (4 mg/ml) minor dimer and trimer peaks potentially result from other weak or unspecific intermolecular interactions. (B) PlxnA2_1-2_ bound to PlxnA2_4-5_ with a K_d_ of 20.9 ± 0.38 μM (I), while PlxnA2_4-5_ F690N/E692S completely abolished binding with PlxnA2_1-2_ (II) revealed by SPR equilibrium experiments. In the illustrations (right), the sema domain, PSI1 domain, PSI2 domain, and IPT2 domain are colored in purple, green, red, and yellow, respectively. The N-linked glycans introduced by the F690N/E692S mutation are represented as blue stars.

Intermolecular interaction between full plexin ectodomains has been shown previously by us for PlxnA2 (Janssen et al., 2010) and by others for PlxnC1 (Liu et al., 2010), but the basis for this association remained unclear. In light of our PlxnA ectodomain structures, we set out to assess whether PlxnAs in solution interact via the sema–PSI2-IPT2 interface. The relatively low resolution of our full-length ectodomain structures mean that the residue-to-residue level details of the interface interactions must be interpreted with caution; we therefore chose to test the sema–PSI2-IPT2 interface by introducing glycosylation sites rather than simple point mutations. We first found that disrupting the sema–PSI2-IPT2 interface by inserting a bulky, N-linked glycan onto either the sema domain (PlxnA1_1-10_ F145N/L147S) or the PSI2 domain (PlxnA1_1-10_ F693N/E695S) can block the PlxnA1–PlxnA1 interaction using multi-angle light scattering (MALS) experiments coupled to size-exclusion chromatography (Figure S4). We then used analytical ultracentrifugation (AUC) sedimentation velocity experiments to demonstrate that in solution PlxnA1_1-10_ exists in heterogeneous states encompassing monomers up to tetramers arising from intermolecular sema–IPT2-PSI2 interactions (Figure 4A). At a high concentration of 28.1 μM (4 mg/ml), PlxnA1_1-10_ gave four distinct peaks with sedimentation coefficients corresponding to monomer, dimer, trimer, and tetramer species, respectively (Figure 4AI). When the concentration was decreased to 7.0 μM (1 mg/ml), the population of PlxnA1_1-10_ dimer and higher order oligomers decreased, and most of the PlxnA1_1-10_ remained monomeric (consistent with the monomeric PlxnA1_1-10_ seen at concentrations of 7 to 33 nM in the EM studies; Figure S2 and Experimental Procedures). The discrete shape of the peaks is indicative of relatively slow exchange between the monomeric and oligomeric states, and the sedimentation coefficients predicted by the structural models for the different oligomeric states fall within the spectrum of each peak. There was no evidence of plexin oligomerization states larger than a tetramer, consistent with the relatively weak intermolecular interactions of PlxnA1_1-10_ being insufficient to build up large oligomers. In order to assess directly the contribution of sema–IPT2-PSI2 interactions, we also analyzed the behavior of PlxnA1_1-10_ F693N/E695S during AUC. This putative interaction-blocking mutant showed one major sedimentation coefficient peak corresponding to the monomer species at both 28.1 μM and 7.0 μM (Figure 4AII).

We then undertook surface plasmon resonance (SPR) binding equilibrium experiments to probe the sema–IPT2-PSI2 interface and determine the affinity of interaction. We engineered two separate segments consisting of the PlxnA2 sema-PSI1 domains (PlxnA2_1-2_) and the PSI2-IPT2 domains (PlxnA2_4-5_); each segment encompasses one side of the putative “head-to-stalk” intermolecular interface. We found that PlxnA2_1-2_ and PlxnA2_4-5_ associated with an equilibrium dissociation constant (K_d_) of 20.9 ± 0.38 μM (Figure 4BI). The introduction of glycosylation by mutation F690N/E692S in PlxnA2_4-5_ completely abrogated the interaction with PlxnA2_1-2_ (Figure 4BII), consistent with our MALS and AUC results for the equivalent mutation in PlxnA1_1-10_ (PlxnA1_1-10_ F693N/E695S). Taken together, the results of our in solution assays point to a head-to-stalk mode of interaction between receptor ectodomains that is common to all PlxnAs.

### FRET-FLIM Confirms PlxnA–PlxnA Interactions via the Sema–PSI2-IPT2 Interface on the Cell Surface

**Figure 5:**
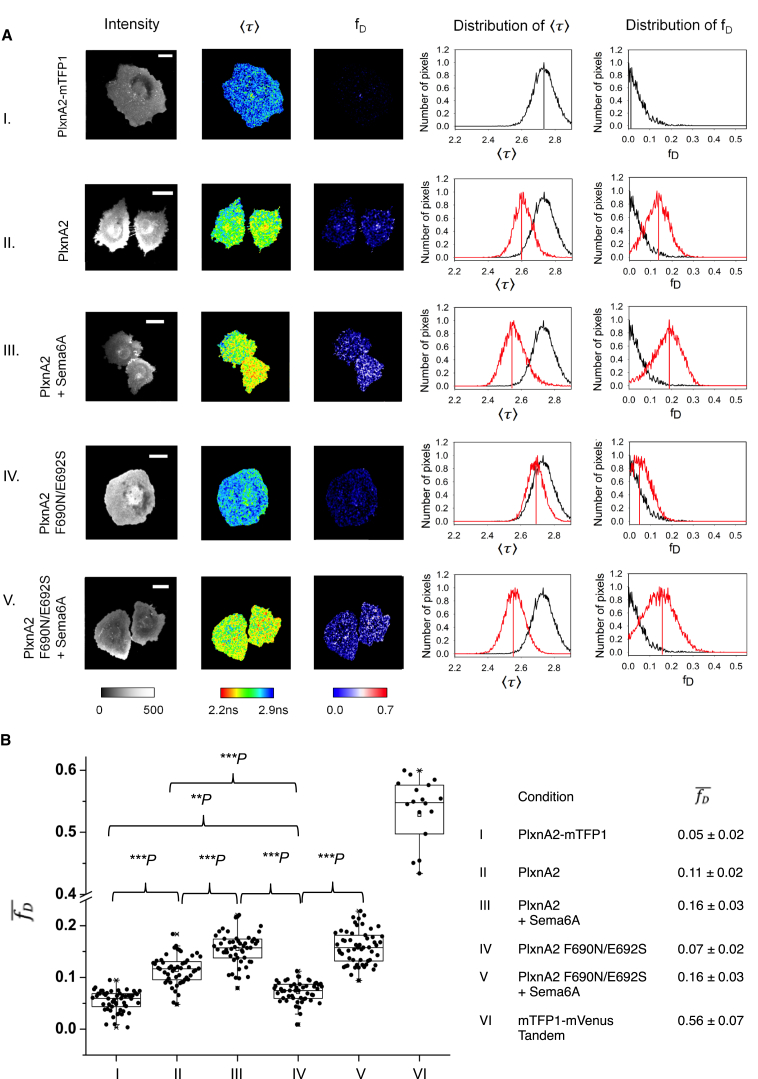
FRET-FLIM in Live COS-7 Cells Indicates Cell Surface PlxnA-PlxnA Head-to-Stalk *cis-*Interactions Occur Pre-Ligand Binding (A) Pixel-by-pixel analysis of representative cells expressing PlxnA2-mTFP1 alone (I), PlxnA2-mTFP1 and PlxnA2-mVenus before (II) and after (III) Sema6A stimulation, and PlxnA2 F690N/E692S-mTFP1 and PlxnA2 F690N/E692S-mVenus before (IV) and after (V) Sema6A stimulation. From left to right, the images show the fluorescence intensity (photon count), 〈τ〉, and *f*_*D*_ in pseudo-color scales. The histograms display the corresponding distribution of 〈τ〉 and *f*_*D*_ (black: PlxnA2-mTFP1 expressed alone; red: mTFP1- and mVenus-fused PlxnA2 or PlxnA2 F690N/E692S coexpressed). The lines within the peaks indicate average values $\bar{\langle \tau \rangle }$ and $\bar{f_{D}}$. Scale bar, 10 μm. (B) The distribution of $\bar{f_{D}}$ for large populations of cells (N > 52) expressing fluorescent-protein-fused PlxnA2s (I to V) and a high-FRET mTFP-mVenus Tandem (VI; Padilla-Parra et al., 2008). The upper and lower quartiles of each sample are represented by the upper and lower sides of the boxes; the medians are represented by the black lines, and the means by hollow points. The range of the whiskers indicates the statistical outliers with a coefficient 1.5. The p values signify the statistical significances between two selected samples determined by paired two-sample t tests. The values of $\bar{f_{D}}$ for each condition are listed in the table (right). ^∗∗^P, p < 0.01. ^∗∗∗^P, p < 0.001. See also Figure S5B.

We next sought information on the oligomeric state of PlxnAs at the cell surface. Our first experiments, using localization microscopy, suggested that PlxnAs form only dimers or small oligomers (Figure S5A). As the size of these structures was too close to the resolution limit (∼50 nm) of localization microscopy, we turned to Förster resonance energy transfer measured by fluorescence lifetime imaging microscopy (FRET-FLIM), a method suited to measure transient and low-affinity interactions (Padilla-Parra et al., 2008, Padilla-Parra et al., 2015, Yamada et al., 2009, Zhao et al., 2014) (Figures 5 and S5B). Fluorescent proteins mTFP1 and mVenus were fused to the PlxnA2 extracellular-transmembrane segment via a RTLEVLFQGP linker to provide the FRET donor (PlxnA2-mTFP1) and acceptor (PlxnA2-mVenus), respectively. The linker was designed to allow FRET to occur if PlxnA2-mTFP1 and mPlxnA2-mVenus interact via the sema–IPT2-PSI2 interface, or through ligand binding (Figure S5BI).

For each FLIM image of a live COS-7 cell, intermolecular interactions were quantified by the mean fluorescence lifetime, 〈τ〉, and the fraction of interacting donor, *f*_*D*_, on a pixel-by-pixel basis (Figure S5BII; see also Supplemental Experimental Procedures) (Padilla-Parra et al., 2015). Average mean lifetime $\left(\bar{\langle \tau \rangle }\right)$ and average fraction of interacting donors $\left(\bar{f_{D}}\right)$ were calculated from these pixel-by-pixel distributions (Figure 5). To exclude potential artifacts due to protein overexpression, we only analyzed cells expressing similar PlxnA levels (assessed by fluorescence intensity). The ratio between FRET donor-fused PlxnAs and acceptor-fused PlxnAs did not correlate with the level of FRET (i.e., extent of PlxnA–PlxnA interactions) (Figure S5BII). A representative cell expressing PlxnA2-mTFP1 alone (no-FRET) has a $\bar{\langle \tau \rangle }$ of 2.73 ns and an $\bar{f_{D}}$ of 0.05 (Figure 5AI). In contrast, a cell co-expressing PlxnA2-mTFP1 and PlxnA2-mVenus shows a $\bar{\langle \tau \rangle }$ of 2.61 ns and an $\bar{f_{D}}$ of 0.11, indicating FRET is occurring (Figure 5AII). A cell co-expressing the putative interaction-inhibiting mutants PlxnA2 F690N/E692S-mTFP1 and PlxnA2 F690N/E692S-mVenus exhibits much reduced FRET, with a longer $\bar{\langle \tau \rangle }$ (2.65 ns) and a lower $\bar{f_{D}}$ (0.07) (Figure 5AIV). These results demonstrate that the sema–PSI2-IPT2 interface indeed mediates PlxnAs *cis*-interactions at the cell surface (for a sample size of >52 cells, p < 0.001; Figures 5BI, 5BII, and 5BIV). After Sema6A stimulation, a representative cell co-expressing PlxnA2-mTFP1 and PlxnA2-mVenus showed a $\bar{\langle \tau \rangle }$ of 2.55 ns and an $\bar{f_{D}}$ of 0.16 (Figure 5AIII). Similarly, a cell co-expressing PlxnA2 F690N/E692S-mTFP1 and PlxnA2 F690N/E692S-mVenus showed a level of FRET as high as the wild-type PlxnA2 ($\bar{\langle \tau \rangle }$ = 2.54 ns and $\bar{f_{D}}$ = 0.16) (Figure 5AV). These results (identical values of $\bar{f_{D}}$ = 0.16 ± 0.03 for >52 cells; Figures 5BIII and 5BV) point to increased dimerization of both wild-type and mutant PlxnA2s on ligand binding. In sum, our fluorescent microscopy-based experiments show compelling evidence for PlxnA *cis*-interactions mediated by the sema–PSI2-IPT2 interface occurring on the cell surface. Semaphorin binding to PlxnAs induces higher levels of interaction, which is most likely independent from the *cis*-interaction-modulated PlxnA pre-association.

### PlxnAs Are Autoinhibited by Head-to-Stalk *cis*-Interactions

**Figure 6:**
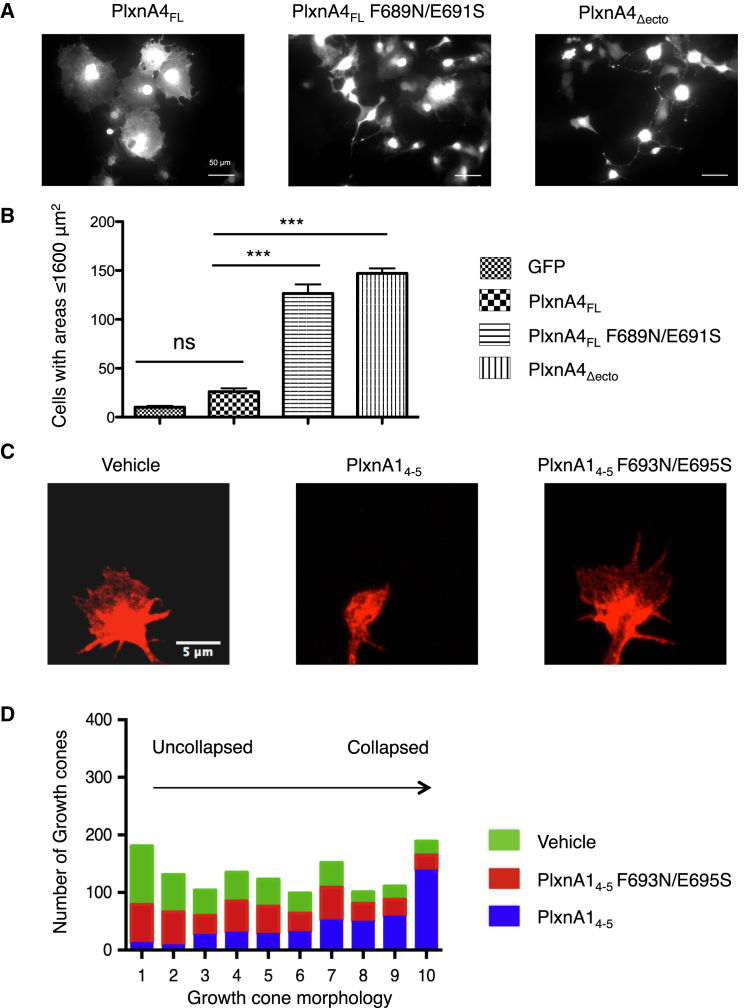
PlxnA *cis*-Interactions Mediate Functional Autoinhibition of PlxnA Signaling (A) Representative images of COS-7 cells transfected with GFP and PlxnA4_FL_, PlxnA4_FL_ F689N/E691S, or PlxnA4_Δecto_ constructs. GFP signal is shown. Scale bar, 50 μm. (B) The average number of GFP-positive COS-7 cells with an area less than 1,600 μm^2^. Data are presented as means ± SEM from three to five independent experiments. N = 350–440 cells per condition. ^∗∗∗^p < 0.0001, one-way ANOVA with Bonferroni multiple comparison test. ns, not significant. (C) Representative images of dentate gyrus (DG) granule cell growth cones (stained for phalloidin in red) treated with vehicle, PlxnA1_4-5_, or PlxnA1_4-5_ F693N/E695S. Scale bar, 5 μm. (D) Morphology of growth cones graded using a growth cone morphology matrix (Figure S6) that ranges from 1 (uncollapsed) to 10 (fully collapsed). Number of growth cones per condition is 453 (vehicle), 431 (PlxnA1_4-5_,), and 442 (PlxnA1_4-5_ F693N/E695S), respectively.

The PlxnA head-to-stalk *cis*-interactions that we observed using various approaches could function to impose pre-signaling autoinhibition. To test this model at the functional level, we first used COS-7 cells as a heterologous system that mimics growth cone collapse (Takahashi and Strittmatter, 2001). As reported previously, COS-7 cells expressing full-length, wild-type PlxnA4 (PlxnA4_FL_) displayed a normal, spread-out morphology (Figure 6A) (Suto et al., 2003). In contrast, expression of PlxnA4_FL_ F689N/E691S in which the sema–PSI2-IPT2 interface is blocked by an N-linked glycan (analogous to that used previously in PlxnA1 and PlxnA2) results in robust cell contraction (Figure 6A). The extent of cell contraction is similar to that induced by PlxnA4_Δecto_, a constitutively active mutant lacking the entire extracellular region (Figure 6B).

To further probe whether *cis*-interactions between PlxnA ectodomains mediate autoinhibitory interactions, dentate gyrus (DG) granule cells from the hippocampus were incubated with wild-type PlxnA1_4-5_ (domains PSI2-IPT2) or its interaction-blocking mutant, PlxnA1_4-5_ F693N/E695S (Figures 6C and S6). PlxnA1_4-5_ (final concentration 200 μM, 10-fold the K_d_ of PlxnA2_4-5_–PlxnA2_1-2_ interactions measured by SPR) was predicted to bind full-length PlxnA molecules on the growth cone surface, thereby counteracting *cis*-interactions between PlxnA molecules (PlxnA1 but also other PlxnAs expressed by DG granule cells) and leading to receptor activation; DG granule cells were used because they endogenously express various PlxnAs and are responsive to a wide range of PlxnA ligands, including Sema3s and Sema6s (e.g., Suto et al., 2007). Following treatment with vehicle, growth cones of DG granule cells showed a typical fan-shaped morphology (Figure 6C). In contrast, incubation with PlxnA1_4-5_, but not PlxnA1_4-5_ F693N/E695S, induced marked growth cone collapse (Figure 6C). These qualitative observations were confirmed by a detailed analysis of growth cone morphology (Figure 6D). Growth cone collapse was graded using a scale from one (uncollapsed) to ten (fully collapsed) according to a matrix of growth cones with different morphologies, allowing the detection of even subtle changes in growth cone morphology (Figure S6) (van Erp et al., 2015). This analysis revealed that whereas PlxnA1_4-5_ induced robust collapse, this effect was not observed when using a construct unable to bind endogenous PlxnAs, PlxnA1_4-5_ F693N/E695S (Figures 6C and 6D). These results coupled with our observations in COS-7 cell contraction assays support a model in which PlxnAs are normally autoinhibited by intermolecular *cis-*interactions. Disruption of these interactions, by introducing mutations or addition of competitive protein domains, induces constitutive signaling and collapse.

## Discussion

The construction of a functional neuronal network by axon guidance cues requires multi-level and spatiotemporal regulation of their growth cone receptors. Most guidance cue receptors are single-pass transmembrane (TM1) class cell-surface receptors, and studies in the past decades have shown that their signaling is often triggered, upon ligand binding, by receptor dimerization followed by clustering. Despite considerable progress in our understanding of the structure and function of axon guidance receptors, the multifaceted regulatory mechanisms that must underlie axon guidance receptor signaling remain poorly understood. These include the mechanisms that prevent premature signaling or that translate ligand binding into the activation of downstream signaling. To address these questions, we have focused on semaphorins and their PlxnA receptors, which have been implicated in the control of various aspects of neural circuit development (Pasterkamp, 2012). Thus far, our understanding of the molecular regulation of semaphorin–PlxnA signaling has been limited by a lack of information on the architecture and interaction modes of full-length PlxnA extracellular segments. The structural, microscopy, and functional data on full-length PlxnA ectodomains we report here show that PlxnA ectodomains are autoinhibited through intermolecular *cis-*interaction prior to ligand binding and support the notion of dimerization-based PlxnA activation upon ligand binding.

**Figure 7:**
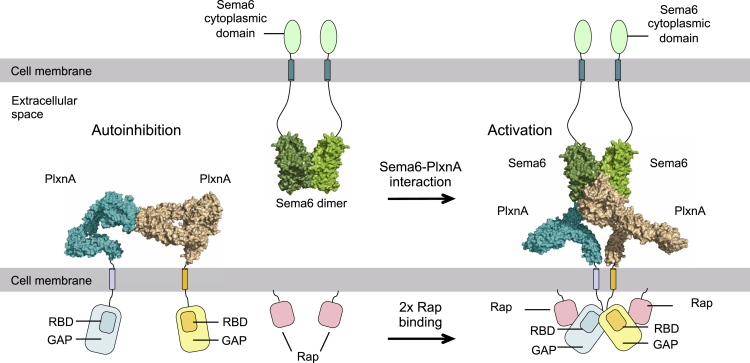
A Structural Model for PlxnA Autoinhibition Pre- and Activation Post-Ligand Binding The PlxnA extracellular domain is represented by the 7.5 Å crystal structure of PlxnA4_1-10_ (space group P4_1_, see Figure S1) and superposed onto the Sema6A_ecto_–PlxnA2_1-4_ complex structure (PDB: 3OKY) (Janssen et. al., 2010). The transmembrane and cytoplasmic region of PlxnA as well as the extracellular C-termini, transmembrane domain, and cytoplasmic region of Sema6 are illustrated as cartoons.

Data from multiple crystal structures of the PlxnA1, PlxnA2, and PlxnA4 ectodomain reveal that the extracellular segment in class A plexins adopts a distinctive, relatively rigid, ring-like conformation. Negative stain EM of PlxnA1_1-10_ also shows this ring-like state to be the favored ectodomain conformation. The sequential arrangement of ten domains to form a well-defined, open ring is unexpected and striking. This distinctive ectodomain architecture immediately provides a satisfyingly simple solution to a longstanding puzzle. The precise arrangement of bivalent semaphorin interfacing with two diverging PlxnA_1-4_ ectodomain fragments is conserved for Sema6A- and Sema3A-containing complexes (Janssen et al., 2010, Janssen et al., 2012). How does a recognition assembly, involving only the N-terminal (sema) of ten “beads-on-a-string” domains, position the membrane proximal tenth domain (IPT6) to initiate dimerization, and consequent activation, in the cytoplasmic region? Superposition of the crystal structure for a full-length PlxnA extracellular segment onto our previously reported Sema6A_ecto_–PlxnA2_1-4_ complex (Janssen et al., 2010) brings the two IPT6 domains into close proximity (Figure 7). The distances between the two PlxnA ectodomain C-termini in these modeled complexes range from 10 Å to 59 Å for the five PlxnA crystal structures that have all ten domains resolved. This positioning of the membrane proximal domains from two PlxnAs appears appropriate to promote interaction between the transmembrane regions and, ultimately, dimerization of the cytoplasmic region. The available structural information for the Sema3-PlxnA-neuropilin complex and neuropilin a1-a2-b1-b2 ectodomain segment (Janssen et al., 2012) can also be accommodated in this model. Based on these observations, we believe that the distinctive ring-like conformation of the PlxnA ectodomain provides a mechanism for efficient and rather precisely defined coupling between extracellular and intercellular dimerization states. Interestingly, negative stain EM uncovers a less-frequent, twisted-open chair-like conformation for PlxnA1_1-10_ generated by a 180° rotation around a midway hinge in the ring-like stalk. The fact that this conformation is the only alternative to the ring-like conformation suggests it may play a role in another aspect of PlxnA signaling, possibly the recently discovered PlxnAs–Sema6s *cis-*interaction that modulates PlxnA signaling in different neuronal populations (Andermatt et al., 2014, Haklai-Topper et al., 2010, Sun et al., 2013). However, uncovering what specific role (if any) the chair-like conformation plays will require significant further experimentation.

Our studies also uncover pre-signaling autoinhibition by intermolecular, head-to-stalk interactions in the receptor ectodomains as a novel regulatory mechanism for PlxnAs. Although the PlxnA head-to-stalk interaction appears relatively weak when measured in solution, for *cis*-interaction occurring between plexins tethered to the same plasma membrane, there is potential for substantial enhancement in binding affinity (Wu et al., 2011). We find that manipulations that disrupt intermolecular *cis-* interactions lead to constitutive plexin signaling and growth cone collapse. Previous work has hinted at the presence of auto-inhibitory intramolecular plexin interactions (Takahashi and Strittmatter, 2001). However, based on the structural data we report, now it seems unlikely that PlxnA ectodomains can engage in intramolecular interactions. Conversely, our work suggests that it is the combination of distinct structure and intermolecular, head-to-stalk interactions of the PlxnA ectodomains that maintains separation of the transmembrane and cytoplasmic segments pre-signaling (Figure 7). One should note that the mutated PlxnAs are not restricted to the auto-inhibited head-to-stalk dimer form; instead, they are free to rearrange and move around on the cell surface. The transmembrane and cytoplasmic regions of two mutated PlxnAs may, in many ways, transiently come into close vicinity, which would inadvertently lead to cytoplasmic domain dimerization and signaling. This conclusion is supported by previous findings (Takahashi and Strittmatter, 2001) that demonstrate that deleting the entire ectodomain or the sema domain of PlxnAs has the same cell-collapsing effect as using a glycan to block the head-to-stalk interface. Data from disparate signaling systems point to the importance of pre-association and ligand-induced conformational reorganization in the regulation of receptor activity (Atanasova and Whitty, 2012). This outside-together-inside-apart mode of PlxnA pre-clustering can prevent cytoplasmic dimerization while permitting localization of receptors poised for rapid response to ligand binding. Such receptor pre-clustering may be necessary for the exquisite spatial-temporal control of signaling required in axon guidance. Whether this previously unsuspected autoinhibitory mechanism, based on intermolecular interactions of receptor ectodomains, is employed by other classes of plexins or axon guidance signaling systems requires further investigation.

## Experimental Procedures

### Protein Production, Crystallization, and Data Collection

Mouse PlxnA1, PlxnA2, and PlxnA4 were produced using the pHLsec vector (Aricescu et al., 2006) and expressed in HEK293S (Reeves et al., 2002) cells for crystallization and in HEK293T cells for all other experiments. Samples were purified from dialysed medium by immobilized metal-affinity and size-exclusion chromatography. Crystallization experiments were set up by mixing 100 nl (or 200 nl for PlxnA2_1-10_) of protein solution with 100 nl reservoir solution at 20.5°C or 4.0°C. Optimization experiments including lysine methylation of protein samples and crystal dehydration are detailed in the Supplemental Experimental Procedures. Crystallographic data was collected at 100 K, and diffraction data were integrated, scaled, and merged with MOSFLM (Leslie, 2006) and SCALA (Evans, 2011) or AIMLESS in CCP4 (1994).

### Structure Solution and Refinement

The sema-PSI2 domains of the 4.0 Å crystal structure of PlxnA1_1-10_ were first solved by molecular replacement in PHASER (McCoy et al., 2007) using the structure of PlxnA2_1-4_ (Janssen et al., 2010) (PDB: 3OKT) (54% sequence identity with PlxnA1_1-4_). To solve the structures of the remaining stalk region, homology models of PlxnA1 domains IPT2-IPT5 were placed by molecular replacement in PHASER, and the resulting structure was further optimized by manual rebuilding in COOT (Emsley and Cowtan, 2004) and refinement in REFMAC (Murshudov et al., 2011) using jelly-body restraints (Nicholls et al., 2012). The 1.36 Å structure of PlxnA2_4-5_ and 2.2 Å structure of PlxnA1_7-10_ were solved by molecular replacement in PHASER using the corresponding partially refined domains from the initial 4.0 Å structure of PlxnA1_1-10_. These refined structures were then used to aid further refinement of the 4.0 Å structure of PlxnA1_1-10_. Our final model is only missing the following residues: 265–271, 1,153–1,164, 1210–1,216, and 1,228–1,236. The solution and refinement of each individual PlxnA1, PlxnA2, PlxnA4, PlxnA2_4-5_, and PlxnA1_7-10_ structures are detailed in the Supplemental Experimental Procedures.

### Negative Stain EM

For the preparation of carbon-coated grids and electron microscope set up, we followed the previously described protocols for negative stain EM (Booth et al., 2011). Samples were imaged at room temperature using an FEI Tecnai T12 electron microscope operating at an acceleration voltage of 120 kV and a dose of ∼15 electrons per Å^2^. Images were taken using a 4k × 4k FEI Eagle™ CCD camera at a magnification of 55,000× with under-focus values ranging from 1.0 to 1.5 μm. Images analysis was performed using a combination of EM processing programs including EMAN2 (Tang et al., 2007), IMAGIC (Van Heel and Keegstra, 1981), SPIDER and WEB (Frank et al., 1996), and WellMAP (Flanagan et al., 2010), as detailed in the Supplemental Experimental Procedures.

### MALS

MALS experiments based on size-exclusion chromatography were performed with a system in which an analytical Superdex S200 10/30 column (GE Healthcare) was coupled to static light-scattering (DAWN HELEOS II, Wyatt Technology), differential refractive index (Optilab rEX, Wyatt Technology), and Agilent 1200 UV (Agilent Techologies) detectors. Data analysis was done using the ASTRA software package (Wyatt Technology).

### AUC

AUC sedimentation velocity experiments were conducted using a Beckman Optima XL-I analytical ultracentrifuge and an An-60 Ti rotor (Beckman) in absorbance mode (280 nm incident light) at 40,000 rpm rotor speed. A total number of 100 scans were collected with one scan every 6 min. Scans 6–100 were analyzed with Sedfit (Schuck, 2000) and a c(s)-based sedimentation profile analysis. Expected sedimentation coefficients of the structural models were predicted using PROHYDROUS-SOMO (Brookes et al., 2010). For the ring-like and chair-like monomer, it was 5.6S and 5.5S, respectively; for the ring-like and chair-like dimers, it was 8.4S and 7.8S, respectively; for the ring-like and chair-like trimers, it was 10.6S and 9.56S, respectively; and for the ring-like and chair-like tetramers, it was 12.4S and 11.2S, respectively. The systematic peak broadening and peak shift to higher absolute sedimentation coefficients in wild-type and mutant experiments is a general observation at decreasing concentrations (Brown et al., 2001).

### SPR

SPR equilibrium experiments were performed on a Biacore T200 instrument (GE Healthcare) at 25°C in SPR running buffer (10 mM HEPES [pH 7.5], 150 mM NaCl, and 0.005% (v/v) Tween 20) and regeneration buffer (2 M magnesium chloride). Wild-type and mutant PlxnA2_4-5_ were enzymatically biotinylated with BirA via an engineered C-terminal tag and attached to streptavidin covalently coupled to the surface of the SPR chip. PlxnA2 was used for SPR experiments because the PlxnA2_1-2_ and PlxnA2_4-5_ segments were much better expressed than their PlxnA1 counterparts. Data analysis was done using Biacore™ T200 Software v2.0 with the nonlinear curve fitting of a 1:1 Langmuir binding model (Langmuir, 1918) to calculate the equilibrium dissociation constant (K_d_) and the maximum analyte binding value (B_max_) via equation ${\text{R}}_{bound}=\left(C_{A}\times {\text{B}}_{\max}\right)/\left(C_{A}+{\text{K}}_{\text{d}}\right)$, where “R_*bound*_” is measured in response units (RU) and C_A_ is the concentration of analyte.

### Förster Energy Transfer by FRET-FLIM

COS-7 cells expressing fluorescently tagged PlxnA2 were imaged using a SP8-SMD Leica microscope from Leica Microsystems (Manheim, Germany) together with a 63×/1.4 oil immersion objective, a 440 nm pulsed laser at 40 MHz coupled with single photon counting electronics (PicoHarp 300) to excite mTFP1, and a Leica Hybrid external detector to record photons. To achieve high signal to noise and minimize artifacts, only cells with 700–1,000 photons per pixel and negligible amount of bleaching after a 2 × 2 image binning were included in the analysis (Leray et al., 2013, Padilla-Parra et al., 2008). The mean fluorescence lifetime (〈τ〉) and fraction of interacting donor (*f*_*D*_) were calculated as described in in the Supplemental Experimental Procedures (Padilla-Parra et al., 2008, Padilla-Parra et al., 2015, Zhao et al., 2014).

### COS7 Collapse Assay

COS7 cells were cultured in DMEM (high glucose) supplemented with 10% FCS, penicillin-streptomycin, and L-glutamine. Cells were plated on glass coverslips at a density of 0.5–1 × 10^4^ cell/cm^2^ in a 12-well plate. At day in vitro (DIV) 1, cells were transfected with 0.5 μg of PlxnA4 constructs in pcDNA3.1 (+) plasmids together with eGFP using X-tremeGENE™ 9 (Roche) following the manufacturer’s instruction. One day after transfection, cells were fixed in 4% PFA and immunostained for GFP. Images were acquired with a 40× objective with AxioScopeA1 (Zeiss) microscope. Cell area was measured using ImageJ as described previously (Takahashi and Strittmatter, 2001).

### DG Growth Cone Assay

DG granule cell cultures were prepared as described (Van Battum et al., 2014, van Erp et al., 2015). In brief, dissociated DG granule cells were prepared from P6–8 mouse hippocampi and cultured in Neurobasal medium supplemented with B-27, L-glutamine, penicillin-streptomycin, and β-mercaptoethanol on coverslips coated with poly-D-lysine (20 mg/ml) and laminin (40 μg/ml) in a humidified incubator at 37°C and 5% CO_2_. On DIV1, cells were treated with vehicle or wild-type or mutant PlxnA1_4-5_ recombinant proteins (3 mg/ml) for 30 min at 37°C, fixed in 4% PFA and sucrose for 20 min at room temperature, and immunostained with anti-βIII-tubulin antibodies and phalloidin. Images were acquired using a 100× objective on an AxioScopeA1 (Zeiss) microscope and analyzed using a growth cone morphology matrix (Figure S6) in an observer-blind manner.

## Author Contributions

Y.K., B.J.C.J., T.M., R.J.P., and E.Y.J. designed the experiments. Y.K., B.J.C.J., and T.M. performed structural and biophysical measurements. Y.K. carried out negative stain EM and light microscopy experiments. R.K. helped to perform localization experiments, C.H.C. was involved in negative stain EM, T.N. was involved in AUC experiments, R.J.C.G. helped interpret EM and AUC data, and S.P.-P. guided and interpreted the FRET-FLIM experiments. V.R.V. conducted COS-7 cell and DG growth cone assays. T.M. and V.R.V. contributed equally to this work. Main authors responsible for writing and editing the paper are Y.K., B.J.C.J., T.M., R.G., S.P.-P., R.J.P., and E.Y.J.

## Accession Numbers

The structure factors and coordinates for PlxnA1_1-10_ (spacegroup P4_3_2_1_2; 4 Å, P2_1_ and P4_3_2_1_2; 6 Å), PlxnA1_7-10_, PlxnA2_1-10_, PlxnA2_4-5_, and PlxnA4_1-10_ (spacegroup P4_1_, P2_1_, P4_3_2_1_2, and P4_3_22) have been deposited in the Protein Data Bank with accession codes PDB: 5L56, 5L59, 5L5C, 5L7N, 5L5G, 5L74, 5L5K, 5L5L, 5L5M, 5L5N.

## Supplemental Information

Document S1. Figures S1–S6, Table S1, and Supplemental Experimental Procedures

Document S2. Article plus Supplemental Information
