# Supplementary material for: Structural Basis for Plexin Activation and Regulation
Source: Neuron. 2016 Aug 3;91(3):548–60. doi: 10.1016/j.neuron.2016.06.018 (PMC4980550; doi:10.1016/j.neuron.2016.06.018)
Supplement: Document S1. Figures S1–S6, Table S1, and Supplemental Experimental Procedures [file mmc1.pdf]

Neuron, Volume *91*

Supplemental Information

Structural Basis for Plexin Activation and Regulation

Youxin Kong, Bert J.C. Janssen, Tomas Malinauskas, Vamshidhar R. Vangoor, Charlotte H. Coles, Rainer Kaufmann, Tao Ni, Robert J.C. Gilbert, Sergi Padilla-Parra, R. Jeroen Pasterkamp, and E. Yvonne Jones

Structural Basis for Plexin Activation and Regulation

Youxin Kong[1*], Bert J.C. Janssen[1, 4*], Tomas Malinauskas[1], Vamshidhar R. Vangoor[2], Charlotte H. Coles[1, 6], Rainer Kaufmann[1, 3], Tao Ni[1], Robert J.C. Gilbert[1], Sergi Padilla-Parra[1], R. Jeroen Pasterkamp[2#] & E. Yvonne Jones[1#]

[1] Division of Structural Biology, Wellcome Trust Centre for Human Genetics, University of Oxford, Oxford, United Kingdom

[2] Department of Translational Neuroscience, Brain Center Rudolf Magnus, University Medical Center Utrecht, Utrecht, The Netherlands

[3] Department of Biochemistry, University of Oxford, Oxford, United Kingdom

[4] Present address: Crystal and Structural Chemistry, Bijvoet Center for Biomolecular Research, Department of Chemistry, Faculty of Science, Utrecht University, Utrecht, The Netherlands

[6] Present address: Laboratory for Axon Growth and Regeneration, German Center for Neurodegenerative Diseases (DZNE), Bonn, Germany.

[*] These authors contributed equally to this work

[#] Correspondence and requests for materials should be addressed to R.J.P. or E.Y.J. (Email: r.j.pasterkamp@umcutrecht.nl or yvonne@strubi.ox.ac.uk).

SUPPLEMENTAL INFORMATION

This file includes:

Supplemental Figures S1 to S6

Supplemental Figure Legends

Supplemental Table S1

Extended Experimental Procedures

Supplemental References

Figure S1

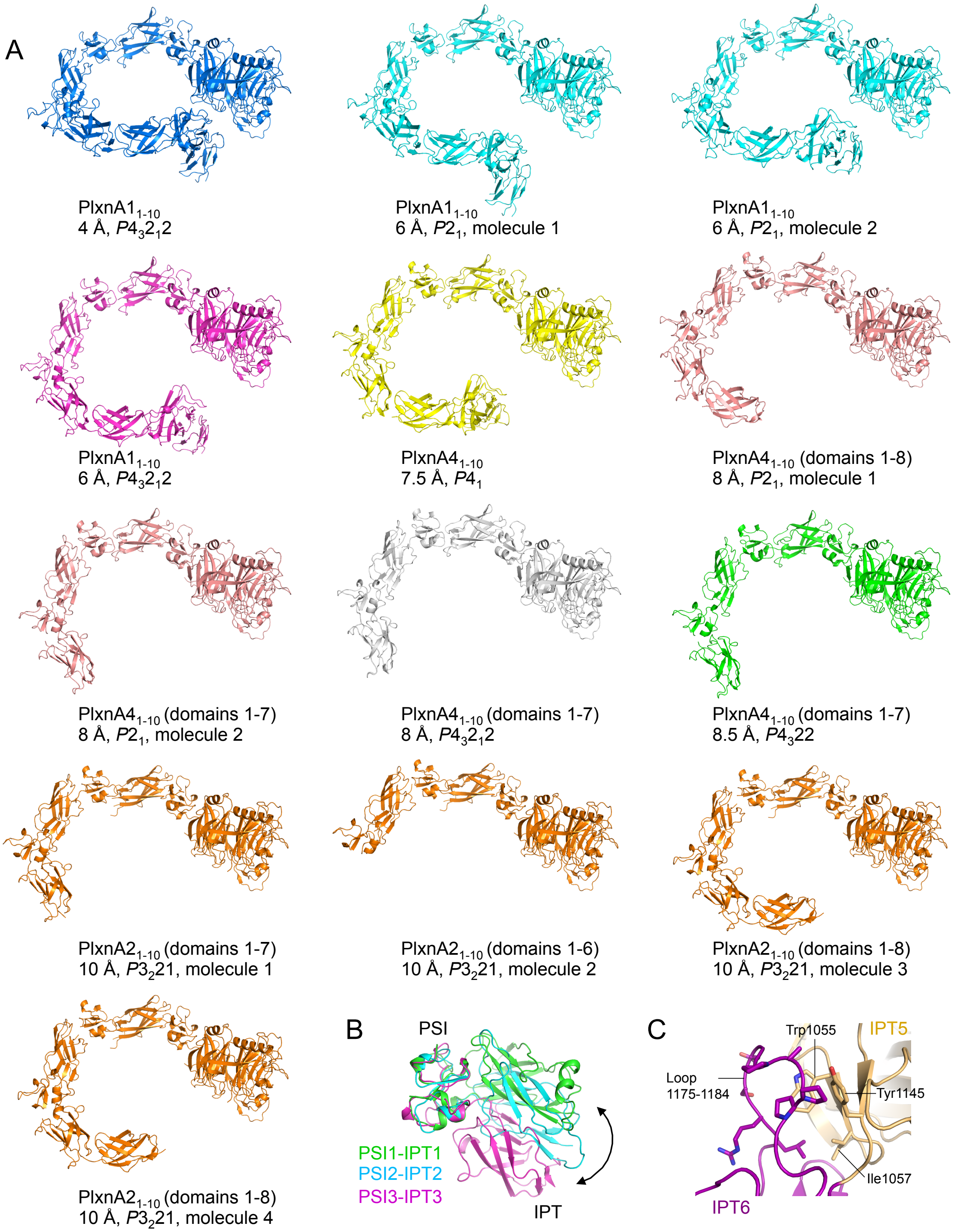
A
PlxnA1$_{1-10}$
4 Å, $P4_32_12$
PlxnA1$_{1-10}$
6 Å, $P2_1$, molecule 1
PlxnA1$_{1-10}$
6 Å, $P2_1$, molecule 2
PlxnA1$_{1-10}$
6 Å, $P4_32_12$
PlxnA4$_{1-10}$
7.5 Å, $P4_1$
PlxnA4$_{1-10}$ (domains 1-8)
8 Å, $P2_1$, molecule 1
PlxnA4$_{1-10}$ (domains 1-7)
8 Å, $P2_1$, molecule 2
PlxnA4$_{1-10}$ (domains 1-7)
8 Å, $P4_32_12$
PlxnA4$_{1-10}$ (domains 1-7)
8.5 Å, $P4_322$
PlxnA2$_{1-10}$ (domains 1-7)
10 Å, $P3_221$, molecule 1
PlxnA2$_{1-10}$ (domains 1-6)
10 Å, $P3_221$, molecule 2
PlxnA2$_{1-10}$ (domains 1-8)
10 Å, $P3_221$, molecule 3
PlxnA2$_{1-10}$ (domains 1-8)
10 Å, $P3_221$, molecule 4
B
PSI
PSI1-IPT1
PSI2-IPT2
PSI3-IPT3
IPT
C
Trp1055
IPT5
Loop
1175-1184
Tyr1145
Ile1057
IPT6

Figure S2

A

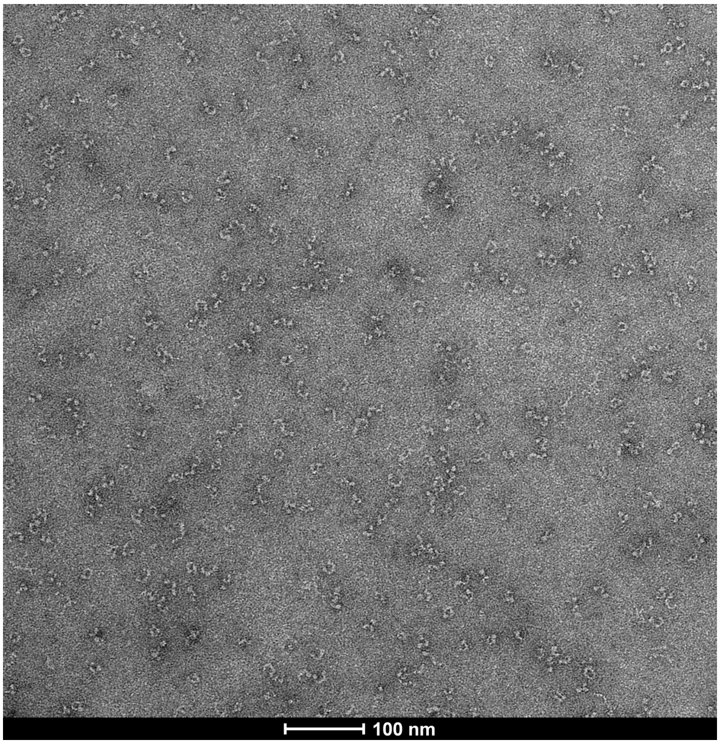

B

I.

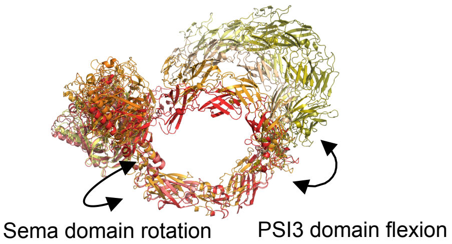

II.

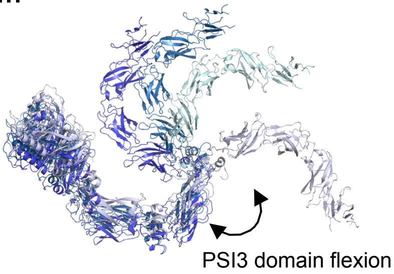

C

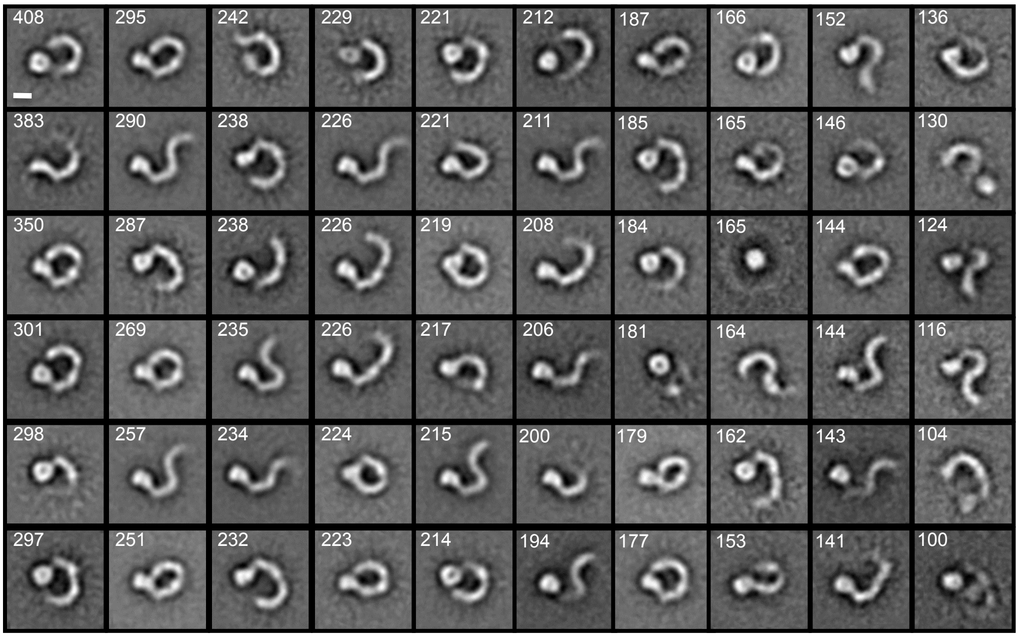

Figure S3

A

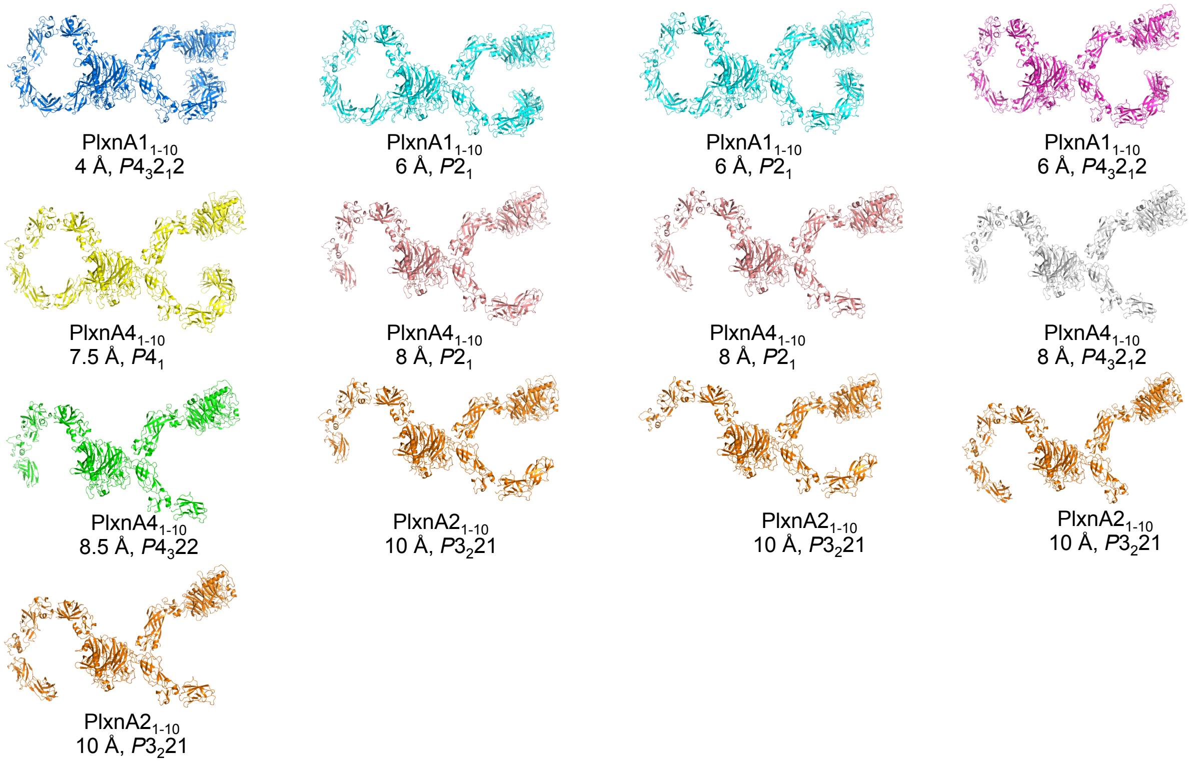

PlxnA1$_{1\text{-}10}$
4 Å, $P4_32_12$
PlxnA1$_{1\text{-}10}$
6 Å, $P2_1$
PlxnA1$_{1\text{-}10}$
6 Å, $P2_1$
PlxnA1$_{1\text{-}10}$
6 Å, $P4_32_12$
PlxnA4$_{1\text{-}10}$
7.5 Å, $P4_1$
PlxnA4$_{1\text{-}10}$
8 Å, $P2_1$
PlxnA4$_{1\text{-}10}$
8 Å, $P2_1$
PlxnA4$_{1\text{-}10}$
8 Å, $P4_32_12$
PlxnA4$_{1\text{-}10}$
8.5 Å, $P4_322$
PlxnA2$_{1\text{-}10}$
10 Å, $P3_221$
PlxnA2$_{1\text{-}10}$
10 Å, $P3_221$
PlxnA2$_{1\text{-}10}$
10 Å, $P3_221$
PlxnA2$_{1\text{-}10}$
10 Å, $P3_221$

B

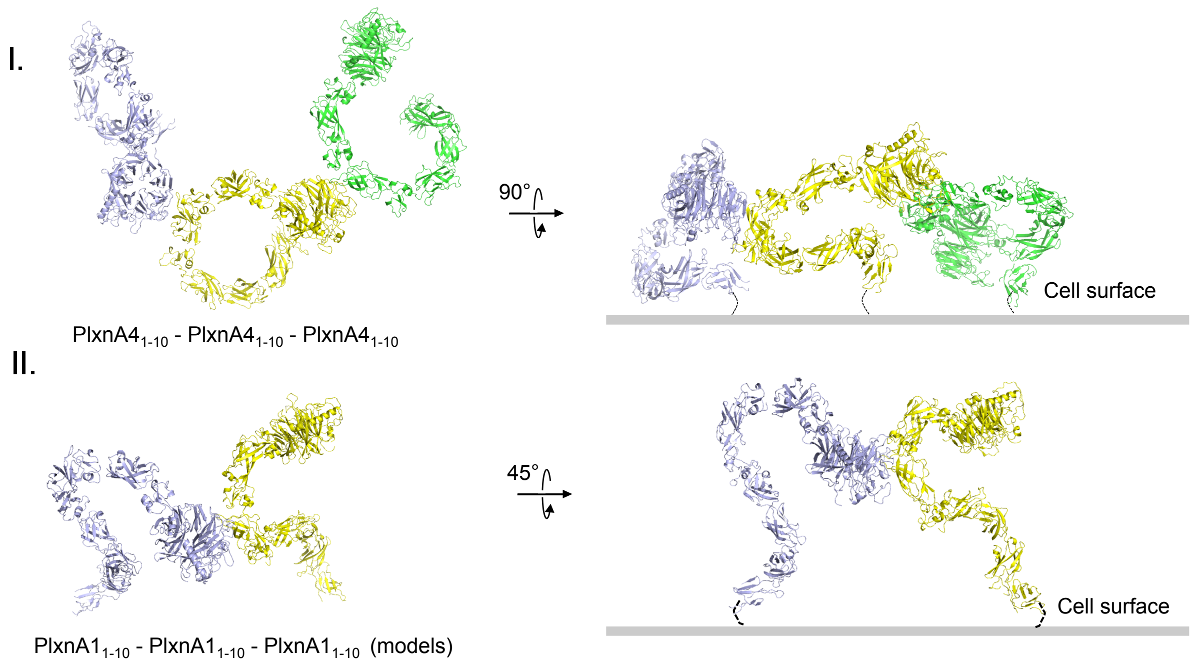

I.
90°
Cell surface
PlxnA4$_{1\text{-}10}$ - PlxnA4$_{1\text{-}10}$ - PlxnA4$_{1\text{-}10}$
II.
45°
Cell surface
PlxnA1$_{1\text{-}10}$ - PlxnA1$_{1\text{-}10}$ - PlxnA1$_{1\text{-}10}$ (models)

Figure S4

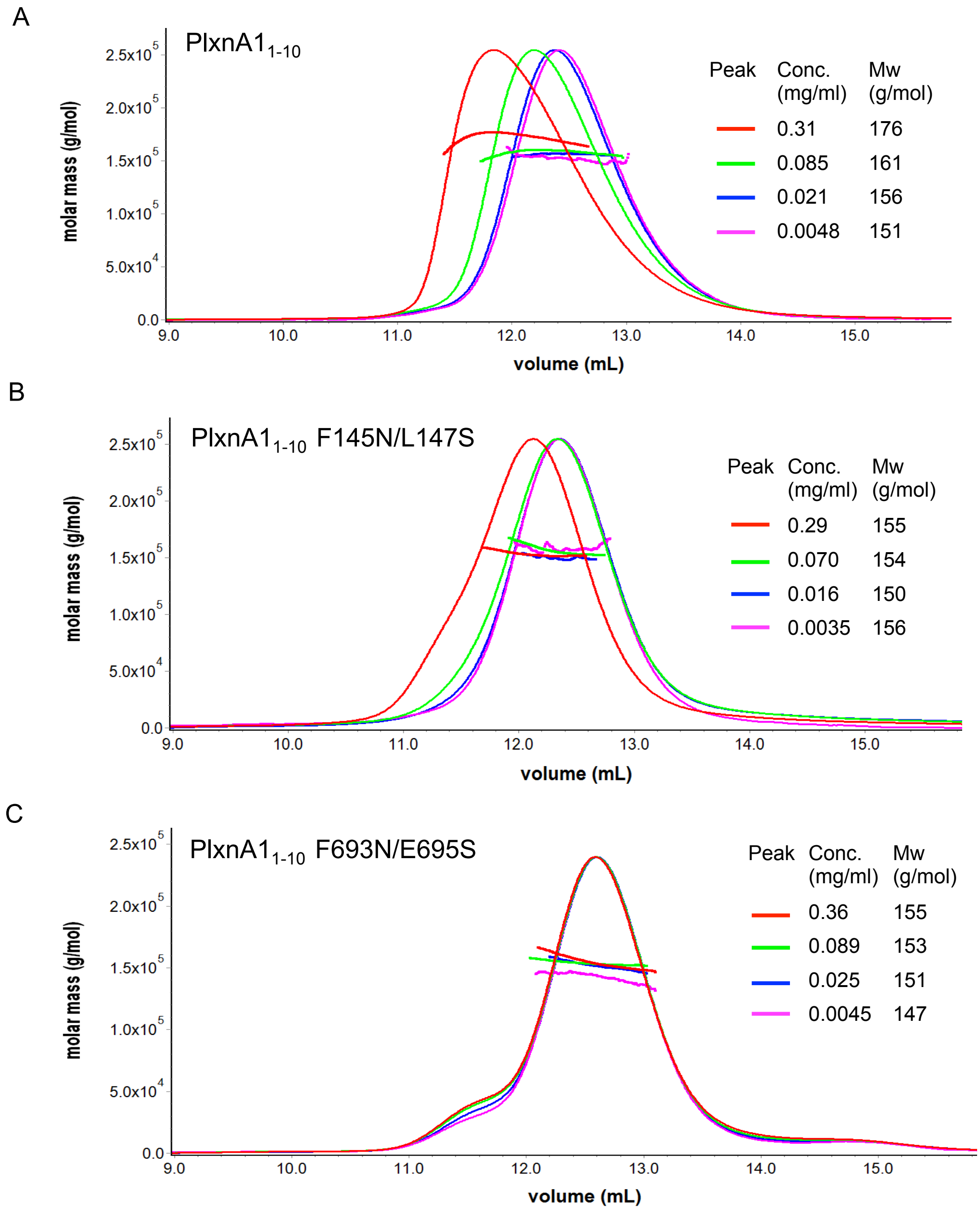

A
PlxnA1 1-10
Peak Conc. (mg/ml) Mw (g/mol)
0.31 176
0.085 161
0.021 156
0.0048 151
molar mass (g/mol)
2.5x10^5
2.0x10^5
1.5x10^5
1.0x10^5
5.0x10^4
0.0
9.0 10.0 11.0 12.0 13.0 14.0 15.0
volume (mL)
B
PlxnA1 1-10 F145N/L147S
Peak Conc. (mg/ml) Mw (g/mol)
0.29 155
0.070 154
0.016 150
0.0035 156
C
PlxnA1 1-10 F693N/E695S
Peak Conc. (mg/ml) Mw (g/mol)
0.36 155
0.089 153
0.025 151
0.0045 147

Figure S5

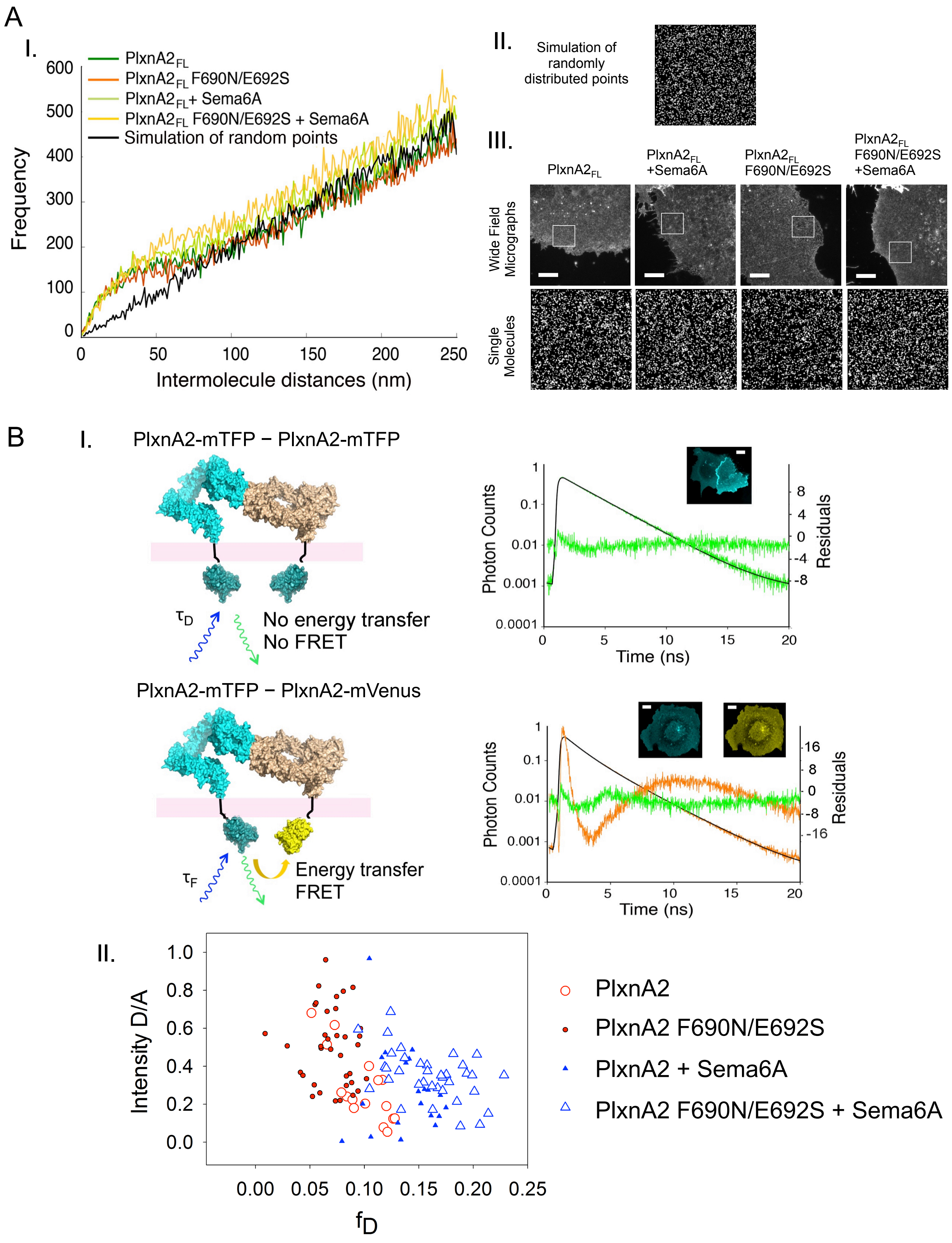
A
I.
PlxnA2FL
PlxnA2FL F690N/E692S
PlxnA2FL+ Sema6A
PlxnA2FL F690N/E692S + Sema6A
Simulation of random points
Frequency
Intermolecule distances (nm)
0
100
200
300
400
500
600
0
50
100
150
200
250
II.
Simulation of randomly distributed points
III.
PlxnA2FL
PlxnA2FL +Sema6A
PlxnA2FL F690N/E692S
PlxnA2FL F690N/E692S +Sema6A
Wide Field Micrographs
Single Molecules
B
I.
PlxnA2-mTFP − PlxnA2-mTFP
τD
No energy transfer
No FRET
Photon Counts
Residuals
Time (ns)
PlxnA2-mTFP − PlxnA2-mVenus
τF
Energy transfer
FRET
II.
Intensity D/A
fD
PlxnA2
PlxnA2 F690N/E692S
PlxnA2 + Sema6A
PlxnA2 F690N/E692S + Sema6A

Figure S6

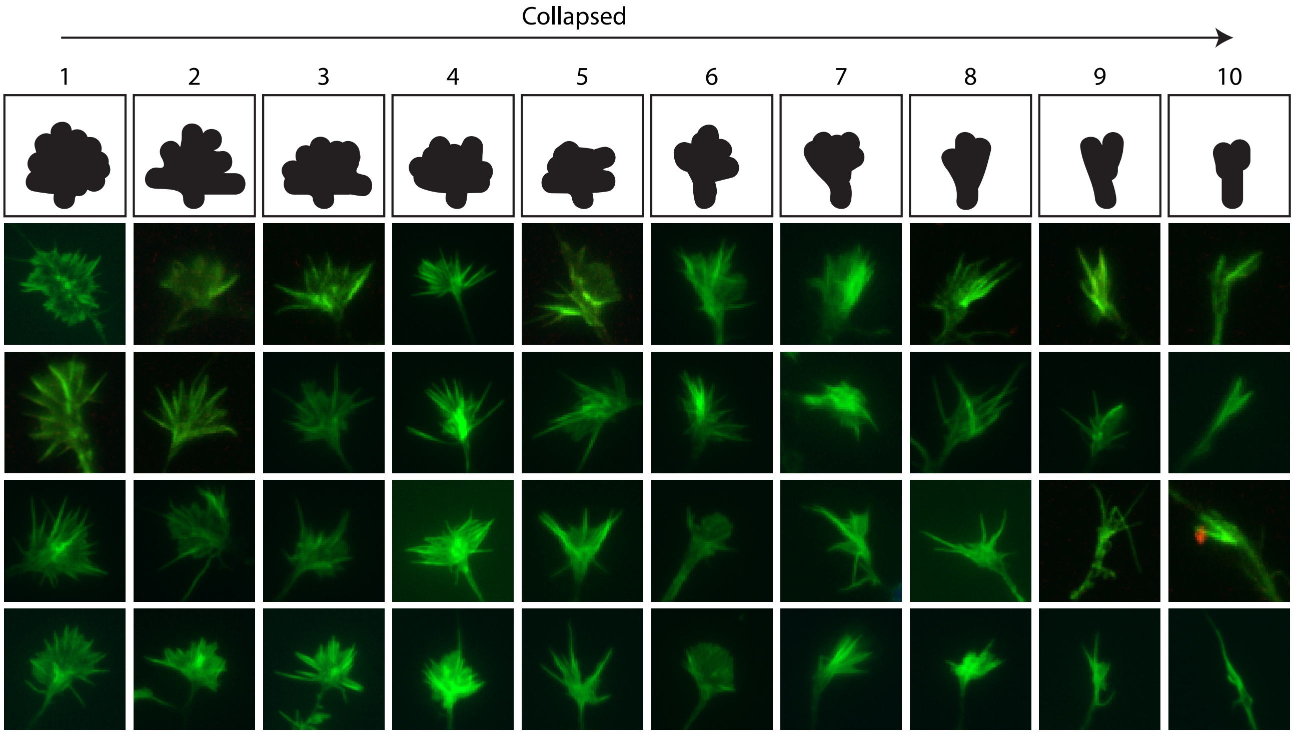

SUPPLEMENTAL FIGURE LEGENDS

Figure S1, related to Figure 1: Crystal structures of the extracellular segments of PlnxA1, PlxnA2 and PlxnA4. (A) Ribbon representation of all full extracellular segment crystal structures of PlxnA1, PlxnA2 and PlxnA4. The domains included in each structure are shown in parenthesis. Highest resolution of each structure and the space group are indicated (see also Table S1). (B) Ribbon representation of a PSI domain based superposition of the three PSI-IPT segments from the 4 Å $PlxnA1_{1\text{-}10}$ structure. The PSI3-IPT3 segment has a different interface and a different interdomain angle compared to the PSI1-IPT1 and PSI2-IPT2 segments. (C) Detailed view of the loop 1175-1184 and residues Trp1055, Ile1057 and Tyr1145 mediated IPT5-IPT6 interaction in $PlxnA1_{7\text{-}10}$.

Figure S2, related to Figure 2 and Extended Experimental Procedures: Negative stain EM and structural modelling of $PlxnA1_{1\text{-}10}$. (A) Representative negative stain electron micrograph of $PlxnA1_{1\text{-}10}$ particles. Scale bar, 100 nm. (B) Modelling of the $PlxnA1_{1\text{-}10}$ ring-like (I) and chair-like (II) conformations. For the ring-like conformations (I), the $PlxnA1_{1\text{-}10}$ stalk region was anchored at the IPT2–PSI3 (domains 5-6) interface and PSI3-IPT6 (domains 6-10) was flexed as a rigid body closer to or away from the N-terminal half of the stalk region. For the alternative, chair-like conformations (II), domains PSI3-IPT6 (domains 6-10) were first rotated by 180° around the IPT2-PSI3 (domains 5-6) linker region (805Y-806K) and then flexed to generate the series of chair-like conformations (II). One should note that the disc-like projection of the sema domain observed for some ring-like class averages required a rotation of the sema domain around the sema-PSI1 linker (Figure S2B I) although this orientation has not been observed in any of the crystal structures. (C) Class averages of $PlxnA1_{1\text{-}10}$ particles ranked from top left to bottom right based on

the number of particles within each class (listed on the top left corner). The size of each image is 298 Å by 298 Å. Scale bar, 5 nm.

Figure S3, related to Figure 3: Cell-surface attached PlxnA dimers and trimers formed via the sema–PSI2-IPT2 interface. (A) Ribbon representation of the ring-like PlxnA–PlxnA dimers formed via the sema–PSI2-IPT2 interface present in all thirteen PlxnA full ectodomain crystal structures. Highest resolution of the diffraction data and the space group are indicated (same coloring scheme as in Figure S1). (B) Possible cell surface orientations of PlxnA trimers based on the ring-like conformation (I) or dimers based on the chair-like conformation (II). The structures in (I) are based on the conformation and packing of the PlxnA4$_{1\text{-}10}$ crystal structure (space group $P4_1$). The chair-like structures in (II) are generated from a model which best correlates with the most populated negative stain EM chair-like class average (see Figure 2 and Figure S2) while keeping the same interface as in (I). Formation of a PlxnA trimer based on the chair-like conformation or a mixture of ring-like and chair-like conformations appeared sterically incompatible with cell surface attachment (data not shown).

Figure S4, related to Figure 4: MALS experiments reveal intermolecular PlxnA1$_{1\text{-}10}$ interaction via the sema–PSI2-IPT2 interface in solution. (A) MALS size exclusion chromatography elution profile of PlxnA1$_{1\text{-}10}$ shows a shift of elution maximum towards larger retention volumes at decreasing injected concentrations, indicating protein–protein interactions. The mass of 151 kDa determined at the lowest concentration fits well with a calculated mass of 156 kDa for a PlxnA1$_{1\text{-}10}$ monomer. (B) PlxnA1$_{1\text{-}10}$ F145N/L147S (an N-linked glycan introduced on the sema domain) has a less pronounced peak shift and the weight average mass indicates a

predominantly monomeric species. Despite a peak shift to lower retention volumes at the highest concentration (4 mg/ml), the measured molar mass of $PlxnA1_{1-10}$ F145N/L147S at all concentrations (150-156 kDa) remains very similar to the $PlxnA1_{1-10}$ monomer. This may be because when high concentrations of $PlxnA1_{1-10}$ F145N/L147S travelled through the SEC column, its initial weak interactions gradually diminished during dilutions in the run. (C) $PlxnA1_{1-10}$ F693N/E695S (an N-linked glycan introduced on the PSI2 domain) is monomeric at all concentrations; there is no peak shift and the weight average mass indicates the presence of only monomers. The red, blue, green and magenta traces correspond to 28.1 μM (4 mg/ml), 7.0 μM (1 mg/ml), 1.8 μM (0.25 mg/ml), and 0.45 μM (0.0625 mg/ml) concentrations at injection, respectively (molecular weight of each peak represented by intersecting lines; concentrations and molecular weights also listed in table).

Figure S5, related to Figure 5 and Extended Experimental Procedures: Fluorescence microscopy experiments reveal PlxnA *cis*-intermolecular interaction on COS-7 cell surface. (A) Single molecule localization microscopy-based cluster analyses reveal that PlxnA2 does not form large cell-surface clusters. Distribution of distances between single PlxnA2 molecules (trace colors as indicated) compared to the distribution of distances between a set of homogenous, randomly distributed points of the same mean density (black trace; I). Sample size, N = 3. A simulated set of homogenous, randomly distributed points following the Poisson process with the same density as the detected single molecules is shown in II. Representative wide-field and single molecule localization microscopy images showing the distribution of $PlxnA2_{FL}$ and $PlxnA2_{FL}$ F690N/692S before and after Sema6A binding are shown in III. The white boxes in the wide-field images represent the regions of interest (ROI; upper panels), from which single receptor molecules

located by our method are indicated in white (bottom panels). Scale bar, 5 μM. (B) FRET-FLIM analysis. When PlxnA2 ectodomain-transmembrane segments fused only to the FRET donor mTFP1 (cyan) interact, no FRET can occur (I; upper panel). The fluorescence lifetime decay of mTFP1 can be fitted to a mono-exponential model (green trace; normalized photon counts). When both PlxnA2 molecules fused to mTPF1 and molecules fused to mVenus (yellow) interact via the sema–PSI2-IPT2 interface, FRET occurs (I; lower panel). In this case the accelerated fluorescence lifetime decay of mTFP1 can be fitted to a bi-exponential model (orange trace). The extent of FRET is independent of donor/acceptor fluorescence intensity ratio as shown by the random distribution of the PlxnA2-mTFP1/PlxnA2-mVenus intensity ratio (y-axis) against the average f_D ($\overline{f_D}$) (x-axis) in II (from > 50 randomly sampled cells).

Figure S6, related to Figure 6: Growth cone morphology matrix for the quantification of DG granule cell growth cone collapse assays. Growth cones with different morphologies ranging from 1 (uncollapsed, fan-like morphology) to 10 (fully collapsed morphology) were selected from our experiments. This generates a matrix which was used to grade growth cones after the treatment of vehicles or purified PlxnA1$_{4\text{-}5}$ proteins (wild type PlxnA1$_{4\text{-}5}$ and mutant PlxnA1$_{4\text{-}5}$ F693N/E695S). Each row represents a range from 1 to 10 with different examples. All growth cones were visualized by phalloidin staining.

SUPPLEMENTAL TABLE

Table S1, related to Figure 1, Figure S1 and Extended Experimental Procedures: Data collection and refinement statistics.

	$PlxnA1_{1\text{-}10}$	$PlxnA1_{1\text{-}10}$	$PlxnA1_{1\text{-}10}$	$PlxnA2_{1\text{-}10}$	$PlxnA4_{1\text{-}10}$
**Data collection**					
Space group	$P4_32_12$	$P2_1$	$P4_32_12$	$P3_221$	$P4_1$
Cell dimensions					
a, *b*, *c* (Å)	195, 195, 176	140, 196, 145	198, 198, 229	238, 238, 642	221, 221, 66
α, β, γ (°)	90, 90, 90	90, 94.6, 90	90, 90, 90	90, 90, 120	90, 90, 90
Resolution (Å)	87-4.0 (4.24-4.00)[a]	58-6.0 (6.57-6.00)[a]	112-6.0 (6.71-6.00)[a]	63-10 (10.5-10.0)[a]	62-7.5 (8.39-7.50)[a]
R_{merge} (%)	24.1 (104)	7.0 (72.9)	7.1 (124)	18.7 (88.2)	20.7 (121)
$I/\sigma I$	5.2 (1.5)	6.8 (1.5)	9.7 (1.6)	5.1 (1.5)	12.2 (2.8)
$CC_{1/2}$	0.95 (0.52)	1.00 (0.50)	1.00 (0.49)	0.99 (0.70)	1.00 (0.59)
Completeness (%)	99.1 (99.8)	99.7 (99.9)	98.8 (98.9)	93.9 (96.8)	99.8 (100)
Redundancy	5.1 (5.1)	3.2 (3.3)	6.6 (6.9)	3.3 (3.6)	6.9 (7.1)
**Refinement**					
Resolution (Å)	87-4.0	58-6.0	112-6.0	63-10.0	55-7.5
Number of reflections	28954	19391	11641	11079	4253
R_{work}/R_{free} (%)	25.7 / 30.8	27.4 / 30.0	30.5 / 31.6	33.5 / 37.0	35.3 / 37.1
Number of atoms	9692	19191	9546	28787	9134
Protein	9692	19191	9546	28787	9134
Ligand/ion	0	0	0	0	0
Water	0	0	0	0	0
B factors ($Å^2$)					
Protein	134	273	283	237	250
Ligand/ion	–	–	–	–	–
Water	–	–	–	–	–
R.m.s. deviations					
Bond lengths (Å)	0.006	0.006	0.006	0.019	0.019
Bond angles (°)	1.2	1.3	1.2	1.7	2.4

	$PlxnA4_{1-10}$	$PlxnA4_{1-10}$	$PlxnA4_{1-10}$	$PlxnA2_{4-5}$	$PlxnA1_{7-10}$
**Data collection**					
Space group	$P2_1$	$P4_32_12$	$P4_322$	$C2$	$C222_1$
Cell dimensions					
a, *b*, *c* (Å)	142, 241, 144	272, 272, 251	189, 189, 253	107.7, 44.6, 33.0	75.9, 82.5, 125.6
α, β, γ (°)	90, 99.8, 90	90, 90, 90	90, 90, 90	90, 104.7, 90	90, 90, 90
Resolution (Å)	48-8.0 (8.95-8.00)[a]	72-8.0 (8.94-8.00)[a]	71-8.5 (9.50-8.50)[a]	52.1-1.36 (1.40-1.36)[a]	62.8-2.2 (2.27-2.20)[a]
R_{merge} (%)	15.8 (76.4)	13.0 (89.4)	33.0 (117)	3.8 (47.6)	12.0 (82.5)
$I/\sigma I$	9.0 (2.9)	14.8 (1.3)	4.2 (1.7)	17.0 (2.3)	9.2 (1.9)
$CC_{1/2}$	1.00 (0.63)	1.00 (0.56)	0.98 (0.67)	1.00 (0.70)	1.00 (0.57)
Completeness (%)	99.5 (100)	99.5 (100)	99.7 (100)	85.6 (41.0)	100 (99.8)
Redundancy	4.1 (4.3)	3.7 (3.9)	5.8 (6.1)	3.7 (3.4)	8.1 (5.0)
**Refinement**					
Resolution (Å)	48-8.0	72-8.0	70-8.5	52.1-1.36	62.8-2.2
Number of reflections	10061	10298	4374	27858	20361
R_{work}/R_{free} (%)	34.9 / 34.9	37.3 / 39.5	40.1 / 41.2	14.1 / 19.6	23.0 / 27.0
Number of atoms	15030	7189	7189	1334	2821
Protein	15030	7189	7189	1228	2680
Ligand/ion	0	0	0	3	11
Water	0	0	0	103	130
B factors (Å^2)					
Protein	264	251	163	16.3	50.4
Ligand/ion	–	–	–	24.9	77.2
Water	–	–	–	29.6	43.6
R.m.s. deviations					
Bond lengths (Å)	0.019	0.019	0.019	0.020	0.002
Bond angles (°)	2.5	2.5	2.5	2.00	0.53

[a]Highest resolution shell is shown in parenthesis.

EXTENDED EXPERIMENTAL PROCEDURES

Cloning and Protein Production

Constructs of mouse versions of PlxnA1$_{1\text{-}10}$, PlxnA1$_{7\text{-}10}$, PlxnA2$_{1\text{-}10}$, PlxnA2$_{1\text{-}2}$, PlxnA2$_{4\text{-}5}$ and PlxnA4$_{1\text{-}10}$ (residues 37-1236, 861-1236, 35-1231, 35-561, 655-804, 36-1229, respectively) were produced using the pHLsec vector (Aricescu et al., 2006) with a C-terminal hexahistidine tag and expressed in HEK 293S (Reeves et al., 2002) and HEK 293T cells. Several interface mutants, PlxnA1$_{1\text{-}10}$ F145N/L147S, PlxnA1$_{1\text{-}10}$ F693N/E695S, PlxnA2$_{4\text{-}5}$ F690N/E692S, PlxnA2$_{1\text{-}10}$ F690N/E692S-mTFP1, PlxnA2$_{1\text{-}10}$ F690N/E692S-mVenus, PlxnA2$_{fl}$-mVenus and PlxnA2$_{fl}$ F690N/E692S-mVenus, were produced via 2-step site directed mutagenesis to introduce N-linked glycosylation sites in the sema–PSI2-IPT2 interface. Ligand proteins used in SPR experiments were cloned into the pHLsec expression vector with a C-terminal hexahistidine tag or a C-terminal BirA recognition sequence for biotinylation (O'Callaghan et al., 1999). Mouse Sema6A (residues 19-571) was cloned into the pHLsec vector resulting in a protein construct with a C-terminal Fc tag that was covalently dimerized. All protein samples were purified from buffer-exchanged medium via dialysis and concentration, followed by immobilized nickel or cobalt metal-affinity chromatography and subsequent size-exclusion chromatography using a prepacked HiLoad 16/600 Superdex 200 prep grade column from GE Healthcare Life Sciences.

Crystallization and Data Collection

Crystallization experiments were conducted by mixing 100 nl (or 200 nl for PlxnA2$_{1\text{-}10}$) of protein solution with 100 nl reservoir solution using a Cartesian Technologies pipetting robot (Walter et al., 2005). Crystallization plates were maintained at 20.5 °C in a TAP Homebase storage vault and imaged with a Veeco visualization system (Mayo et al., 2005). PlxnA1$_{1\text{-}10}$ was concentrated to 8.2 mg/ml in 20 mM HEPES, pH

7.5 and 150 mM sodium chloride and subsequently crystallized in three different crystal forms. One crystal form, with space group $P4_32_12$ grown in 6% (w/v) PEG 4k and 5 mM tricine, pH 8.5 (or 5 mM TRIS, pH 8.5), diffracted to 6.0 Å resolution. Dehydration of these crystals in the plate by increasing the PEG 4k concentration in the mother liquor in three steps to 25% over a time span of 48 hours produced a second crystal form with a 53 Å shorter *c*-axis, that diffracted to 4.0 Å resolution. A third crystal form with space group $P2_1$, diffracting to 6.0 Å resolution, was grown in 15% (v/v) propanol, 20 mM magnesium chloride and 50 mM MES, pH 6.0. For $PlxnA2_{1\text{-}10}$ we obtained one crystal form. $PlxnA2_{1\text{-}10}$ was concentrated to 6.6 mg/ml in 20 mM HEPES, pH 7.5 and 150 mM sodium chloride and subsequently deglycosylated with endoglycosidase F1. Crystals of $PlxnA2_{1\text{-}10}$ with space group $P3_221$ were produced in 0.68 M potassium/sodium tartrate and 85 mM HEPES, pH 7.5 and diffracted to 10 Å resolution. $PlxnA4_{1\text{-}10}$ was crystallized in four different crystal forms. $PlxnA4_{1\text{-}10}$ concentrated to 3.8 mg/ml in 20 mM HEPES, pH 7.5, 150 mM sodium chloride, 100 mM dimethylbenzylammonium propane sulfonate (Non-Detergent Sulfobetaine 256; NDSB-256) and deglycosylated with endoglycosidase F1, crystallized in space group $P4_1$ in 29% (v/v) glycerol, 4.3% (w/v) PEG 8k and 53 mM TRIS hydrochloride, pH 8.5 and diffracted to 7.5 Å resolution. $PlxnA4_{1\text{-}10}$ concentrated to 4.2 mg/ml in 10 mM citrate, pH 5.1 yielded a second and third crystal form. Crystals with space group $P2_1$ were obtained in 0.5 M sodium succinate, 100 mM bis-TRIS propane, pH 7.0 and diffracted to 8.0 Å resolution and crystals with space group $P4_32_12$ were grown in 0.8 M ammonium sulfate, 0.4 M dimethyl (2-hydroxyethyl) ammonium propane sulfonate (NDSB-211), 100 mM TRIS, pH 8.0 and diffracted to 8 Å resolution. $PlxnA4_{1\text{-}10}$ concentrated to 5.8 mg/ml in 20 mM HEPES, pH 7.5, 150 mM sodium chloride and 100 mM NDSB-256 crystallized in a fourth space group, $P4_322$, in 1.5 lithium sulfate, 100 mM TRIS, pH 8.5 and diffracted to 8.5

Å resolution. $PlxnA2_{4-5}$ was concentrated to 6.5 mg/ml in 10 mM HEPES pH 7.5, 150 mM sodium chloride, and crystallized in space group C2, in 25% (w/v) polyethylene glycol 3,350 and 100 mM BIS-TRIS pH 5.5. Lysine methylation of $PlxnA1_{7-10}$ using dimethylamine-borane complex and formaldehyde was performed as described earlier (Walter et al., 2006). $PlxnA1_{7-10}$ was concentrated to 7.5 mg/ml in 10 mM TRIS, pH 8.0, 150 mM sodium chloride, and crystallized in space group $C222_1$, in 25% (w/v) polyethylene glycol 3,350, 100mM BIS-TRIS pH 5.5 and 200mM potassium sodium tartrate. Before diffraction data collection crystals were soaked in mother liquor supplemented with 12.5% (v/v) 2,3-butanediol for $PlxnA1_{1-10}$ crystals, 25% (v/v) glycerol for $PlxnA2_{1-10}$, $PlxnA4_{1-10}$ and $PlxnA2_{4-5}$ crystals, or 25% ethylene glycol for $PlxnA1_{7-10}$ crystals (for $PlxnA4_{1-10}$ crystals in space group $P4_1$ nothing extra was added). Data were collected at 100 K at Diamond beamlines I02, I03, I04, I04-1 and I24 and European Synchrotron Radiation Facility beamline ID23-2. Diffraction data were integrated, scaled and merged with MOSFLM (Leslie, 2006) and SCALA (Evans, 2011) or AIMLESS in CCP4 (CCP4, 1994).

Structure solution and refinement

The structure of $PlxnA1_{1-10}$ at 4.0 Å resolution was initially solved by molecular replacement in PHASER (McCoy, 2007; McCoy et al., 2007) using the structure of $PlxnA2_{1-4}$ (Janssen et al., 2010) (PDB: 3OKT) (54% sequence identity with $PlxnA1_{1-4}$). After solvent flattening in PARROT (Cowtan, 2010) this solution revealed clear electron density for PlxnA1 domains IPT2, PSI3, IPT3, IPT4 and IPT5 that were not included in the search model. To further complete the structure of $PlxnA1_{1-10}$ we generated homology models of these domains with MODELLER (Fiser and Šali, 2003) using the following template PDB–PlxnA1 domain combinations; PDB: 3OKY (Janssen et al., 2010) for IPT2, PDB: 3NVN (Liu et al., 2010) for PSI3, PDB: 2UZY (Niemann et al., 2007) for IPT3 and IPT4, and PDB: 2CXK (unpublished results) for

IPT5, with 16%, 29%, 29%, 29% and 20% sequence identity, respectively. These models were placed by molecular replacement in PHASER and the resulting structure was further optimized by manual rebuilding in COOT (Emsley and Cowtan, 2004) and refinement in REFMAC (Murshudov et al., 2011) using jelly-body restraints (Nicholls et al., 2012). However, the low 4.0 Å resolution of the data prevented unambiguous assignment of the sequence register for those parts of the structure for which no high quality homology model was available, for example for domains IPT2 and IPT5. Furthermore, the weaker electron density of domain IPT6 prevented reliable building of this domain. We therefore sought to determine much higher resolution crystal structures for these segments in isolation. We collected 1.36 Å resolution data from $PlxnA2_{4-5}$ crystals and 2.2 Å resolution data from $PlxnA1_{7-10}$ crystals. The structures of $PlxnA2_{4-5}$ and $PlxnA1_{7-10}$ were solved by molecular replacement in PHASER using the corresponding partially refined domains PSI2-IPT2 and IPT3-IPT4-IPT5, respectively, from the $PlxnA1_{1-10}$ structure. Both solutions were completed by model building in COOT and refinement in REFMAC (for $PlxnA2_{4-5}$) and BUSTER (Smart et al., 2012) and PHENIX (Adams et al., 2002) (for $PlxnA1_{7-10}$). For domain IPT6 of $PlxnA1_{7-10}$ our maps showed only fragmentary electron density for two loops (residues 1153-1163 and 1210-1217) and the C-terminal residues (1228-1236), these regions were therefore not modelled. We used these refined structures to replace domains IPT2, IPT3, IPT4, IPT5 and segments of domain IPT6 in the $PlxnA1_{1-10}$ structure which was then completed by model building in COOT and refinement in REFMAC using jelly-body restraints.

All other PlxnA full extracellular segment structures were solved using the refined 4.0 Å resolution $PlxnA1_{1-10}$ structure and, for $PlxnA2_{1-10}$, also the $PlxnA2_{1-4}$ structure (Janssen et al., 2010), PDB: 3OKT. Homology models for PlxnA2 and PlxnA4 were calculated with MODELLER using the $PlxnA1_{1-10}$ structure as the template for PlxnA2

domains 5 to 10 (IPT2, PSI3, IPT3, IPT4, IPT5 and IPT6) (53% sequence identity) and for all ten PlxnA4 domains (54% sequence identity). The other PlxnA1$_{1-10}$, PlxnA2$_{1-10}$ and PlxnA4$_{1-10}$ crystal forms were solved by molecular replacement in PHASER using individual domains of the PlxnA1$_{1-10}$ structure, of the PlxnA2$_{1-4}$ structure and PlxnA2$_{5-10}$ homology model, and of the PlxnA4$_{1-10}$ homology model, respectively, as search models. Domains were omitted from the models in cases where the electron density was too weak for interpretation due to flexibility (see Figure S1 for the entire set of structures and domains present). All solutions were refined by rigid-body refinement in PHENIX with each domain as a rigid group and a single *B* factor per domain, thus preventing any overfitting. Refinement statistics for all structures are given in Table S1. Electrostatic charge distribution was calculated with PDB2PQR (Dolinsky et al., 2004) and APBS (Baker et al., 2001), alignments were generated with Clustal Omega (Sievers et al., 2011), residue conservation was calculated with The Consurf Server (Glaser et al., 2003) or MODELLER and buried surface areas were calculated with PISA (Krissinel and Henrick, 2007). Figures were produced with The PyMOL Molecular Graphics System (Schrödinger, LLC).

Negative stain electron microscopy images analysis

For the preparation of carbon-coated grids and electron microscope set up, we followed the previously described protocols for negative stain EM (Booth et al., 2011). In brief, 2.5 µl of freshly gel-filtrated PlxnA1$_{1-10}$ at a concentration of 1-5 μg/ml in 10 mM HEPES, pH 7.5 and 150 mM sodium chloride was adsorbed to a glow-discharged carbon-coated copper grid, washed with two drops of deionized water, and stained with two drops of freshly prepared 0.75% uranyl formate. From 355 electron micrographs, a total of 12,645 particles were manually selected from 355 images using EMAN2 (Tang et al., 2007) and framed into boxes with a size of 298 Å × 298 Å. The particle images were normalized, filtered, centered, iteratively aligned

and classified without any starting reference using IMAGIC (van Heel et al., 1996) to generate 60 class averages. The structural models based on the 4 Å crystal structure of PlxnA1$_{1-10}$ were generated manually using The PyMOL Molecular Graphics System (Schrödinger, LLC). The two-dimensional projections of our models were then subjected to automated correlation analysis with the class averages in which all the class averages were compared to all projections of all models. Thirteen models were tested and of these seven were found to be sufficient to represent the experimental class averages. Two-dimensional projections of the crystal structure and models were generated using SPIDER and WEB (Frank et al., 1996) and WellMAP (Flanagan et al., 2010) software and the correlations of the class averages were performed using scripts operating through SPIDER.

Single molecule localization microscopy-based cluster analysis

COS-7 cells were transiently transfected with PlxnA2 full-length constructs conjugated to mVenus at the C-termini via X-treme GENE HP Transfection Reagent and incubated at 37 °C, 5% CO_2 for 24 hours before fixing with 4% paraformaldehyde on ice. The fixed cells were mounted onto the glass coverslip using Prolong® Gold antifate reagent from Life Technologies™. We used a single molecule localization microscopy technique based on the stochastic switching of standard fluorescent proteins (Lemmer et al., 2008), similar to the principle of (F)PALM (Betzig et al., 2006) and STORM (Rust et al., 2006) which rely on special photo-activatable or photo-switchable fluorophores. To perform localization microscopy, we used an OMX (optical microscope experimental, V2, API) microscopic system modified to enable single molecule localization microscopy using conventional fluorescent proteins according to the method described previously (Lemmer et al., 2008). The intensity of the 488 nm laser was adjusted to ~14 kW/cm² in the object plane for localization microscopy imaging of the mVenus conjugated samples. Single molecule positions

were determined using an algorithm based on a maximum-likelihood method optimized for localization microscopy data (Grull et al., 2011) and adapted to the OMX hardware configuration. Distance and cluster analyses of the single molecule data were performed with algorithms described earlier (Kaufmann et al., 2011; Seiradake et al., 2013).

Imaging PlxnA2 interactions in live COS-7 cells using FRET-FLIM

COS 7 cells transiently transfected with PlxnA2 constructs and the mTFP-mVenus Tandem together with GeneJuice transfection reagent (Novagen) were grown to 60–70% confluency on glass–bottom 35 mm Petri dishes (Mattek) in Dulbecco's modified eagle medium with 10% fetal bovine serum. Before imaging, medium was changed to 0.5% fetal bovine serum PBS imaging buffer without phenol red and the observation chamber was mounted onto a SP8–SMD Leica microscope (Leica Microsystems, Germany). A suitable image field focusing on the surface of a robust COS-7 cell was chosen with a 63×/1.4 NA oil immersion objective. For each FLIM image, mTFP1 was excited by a 440 nm pulsed laser (Picoquant GmbH, Germany) at 40 MHz using single photon counting electronics (PicoHarp 300). Emitted single photons from mTFP1 passing through a 460–500 nm filter were subsequently detected by a HyD detector (Leica Microsystems). The acquired fluorescence decay, $i(t)$, of each pixel in one whole cell were deconvoluted with the instrument response function (IRF) and fitted by a Marquandt nonlinear least–square algorithm (Marquardt, 1963) with mono- or bi-exponential theoretical models using Symphotime software from Picoquant GmbH (detailed in Padilla-Parra et al., 2008; 2015). In brief, the mean fluorescence lifetime $\langle\tau\rangle$ of a fluorescence decay $i(t)$, is defined by the following equation:

$$\langle\tau\rangle = \int t \times i(t)dt \Big/ \int i(t)dt \qquad Eq.1$$

The fluorescence lifetime obtained from a mono-exponential decay model of cells expressing only PlxnA2 molecules fused to the donor mTFP1 was first assigned. This lifetime was then fixed as the non-interacting fraction in a bi-exponential model obtained from the fluorescence lifetime decay profile of cells expressing both PlxnA2 fused to mTFP1 and mVenus (Padilla-Parra et al., 2008) using the following equation:

$$i(t) = (1 - f_D)e^{-t/\tau_D} + f_D e^{-t/\tau_F} \qquad Eq.2$$

where f_D stands for the fraction of interacting donor, τ_D is the fixed donor lifetime and τ_F is the discrete FRET lifetime. The value of f_D per pixel was calculated from the mean fluorescence lifetime ($\langle\tau\rangle$) using 1200 channels of fluorescence decay (Padilla-Parra et al., 2008; 2015). One should note that we were only capable of detecting ~1/4 of the real interaction (0.5 because of our labelling strategy and ~0.5 given the dynamic range of the FRET couple (Galperin et al., 2004; Padilla-Parra et al., 2009). This factor together with the spectral heterogeneity of the fluorophores and the temporal resolution of our technique determined that the f_D represents the minimal fraction of the actual interaction (Padilla-Parra et al., 2008; 2015; Yasuda, 2006).

DG growth cone assay

DG granule cell cultures were prepared as described previously (Van Battum et al., 2014). In brief, hippocampi were dissected from P6-8 C57bl6J mice and cut into 350 µm slices using a tissue chopper (McIllwain). The DG was dissected from these slices and collected in 1x Krebs medium (0.7% NaCl, 0.04% KCl, 0.02% KH_2PO_4, 0.2%$NaHCO_3$, 0.25% glucose and 0.001% phenol red). Cells were dissociated after

incubation in 1x Trypsin in Krebs medium for 15 min at 37°C. The reaction was stopped by adding 20 mg/ml soybean trypsin inhibitor followed by trituration using a firepolished Pasteur pipette in Krebs medium containing 2 mg soybean trypsin inhibitor and 20 μg/ml DNAseI. Dissociated DG granule cells were resuspended in Neurobasal medium supplemented with B-27, L-glutamine, penicillin-streptomycin and β-mercaptoethanol and plated on coverslips coated with poly-D-lysine (20 mg/ml) and laminin (40 μg/ml) in 24 well plate and incubated in a humidified incubator at 37°C and 5% CO_2. Proteins were filtered using 0.45 μm filter before incubation with cells. On day *in vitro* (DIV)1, cells were treated with vehicle, or wild type or mutant $PlxnA1_{4-5}$ recombinant proteins (3 mg/ml) for 30 min at 37°C, fixed in 4% PFA and sucrose for 20 min at room temperature, and immunostained with anti-βIII-tubulin antibodies and phalloidin. Images were acquired using a 100× objective on an AxioScopeA1 (Zeiss) microscope and analyzed using a growth cone morphology matrix (Figure S6) in an observer-blind manner. The matrix was composed of 40 growth cones from different experiments that represent the full range of morphologies observed, ranging from a normal fan-shaped morphology (1) to full blown collapse (10). This strategy was applied as it allows the detection of subtle changes in growth cone morphology rather than just a comparison of collapse versus non-collapse.

SUPPLEMENTAL REFERENCES

Adams, P.D., Grosse-Kunstleve, R.W., Hung, L.W., Ioerger, T.R., McCoy, A.J., Moriarty, N.W., Read, R.J., Sacchettini, J.C., Sauter, N.K., and Terwilliger, T.C. (2002). PHENIX: building new software for automated crystallographic structure determination. Acta Crystallogr. D Biol. Crystallogr. *58*, 1948–1954.

Aricescu, A.R., Lu, W., and Jones, E.Y. (2006). A time- and cost-efficient system for high-level protein production in mammalian cells. Acta Crystallogr. D Biol. Crystallogr. *62*, 1243–1250.

Baker, N.A., Sept, D., Joseph, S., Holst, M.J., and McCammon, J.A. (2001). Electrostatics of nanosystems: Application to microtubules and the ribosome. Proc. Natl. Acad. Sci. U.S.A. *98*, 10037–10041.

Betzig, E., Patterson, G.H., Sougrat, R., Lindwasser, O.W., Olenych, S., Bonifacino, J.S., Davidson, M.W., Lippincott-Schwartz, J., and Hess, H.F. (2006). Imaging Intracellular Fluorescent Proteins at Nanometer Resolution. Science *313*, 1642–1645.

Booth, D.S., Avila-Sakar, A., and Cheng, Y. (2011). Visualizing proteins and macromolecular complexes by negative stain em: from grid preparation to image acquisition. J. Vis. Exp. http://dx.doi.org/10.3791/3227.

Cowtan, K. (2010). Recent developments in classical density modification. Acta Crystallogr. D Biol. Crystallogr. 66(4), 470-478.

Dolinsky, T.J., Nielsen, J.E., McCammon, J.A., and Baker, N.A. (2004). PDB2PQR: an automated pipeline for the setup of Poisson-Boltzmann electrostatics calculations. Nucleic Acids Res. *32*, W665–W667.

Emsley, P., and Cowtan, K. (2004). Coot: model-building tools for molecular graphics. Acta Crystallogr. D Biol. Crystallogr. *60*, 2126–2132.

Evans, P.R. (2011). An introduction to data reduction: space-group determination, scaling and intensity statistics. Acta Crystallogr. D Biol. Crystallogr. *67*, 282–292.

Fiser, A., and Šali, A. (2003). Modeller: Generation and Refinement of Homology-Based Protein Structure Models. Methods Enzymol. *374*, 461–491.

Flanagan, J.F., Namy, O., Brierley, I., and Gilbert, R.J.C. (2010). Direct observation of distinct A/P hybrid-state tRNAs in translocating ribosomes. Structure *18*, 257–264.

Frank, J., Radermacher, M., Penczek, P., Zhu, J., Li, Y., Ladjadj, M., and Leith, A. (1996). SPIDER and WEB: Processing and Visualization of Images in 3D Electron Microscopy and Related Fields. J. Struct. Biol. *116*, 190–199.

Galperin, E., Verkhusha, V.V., and Sorkin, A. (2004). Three-chromophore FRET microscopy to analyze multiprotein interactions in living cells. Nat. Meth. *1*, 209–217.

Glaser, F., Pupko, T., Paz, I., Bell, R.E., Bechor-Shental, D., Martz, E., and Ben-Tal, N. (2003). ConSurf: Identification of Functional Regions in Proteins by Surface-Mapping of Phylogenetic Information. Bioinformatics *19*, 163–164.

Gruell, F., Kirchgessner, M., Kaufmann, R., Hausmann, M. & Kebschull, U. Accelerating image analysis for localization microscopy with FPGAs. (2011) International Conference on Field Programmable Logic and Applications 1–5 (IEEE).

Janssen, B.J.C., Robinson, R.A., Pérez-Branguli, F., Bell, C.H., Mitchell, K.J., Siebold, C., and Jones, E.Y. (2010). Structural basis of semaphorin-plexin signalling. Nature *467*, 1118–1122.

Kaufmann, R., Müller, P., Hildenbrand, G., Hausmann, M., and Cremer, C. (2011). Analysis of Her2/neu membrane protein clusters in different types of breast cancer cells using localization microscopy. J. Microsc. *242*, 46–54.

Krissinel, E., and Henrick, K. (2007). Inference of Macromolecular Assemblies from Crystalline State. J. Mol. Biol. *372*, 774–797.

Lemmer, P., Gunkel, M., Baddeley, D., Kaufmann, R., Urich, A., Weiland, Y., Reymann, J., Müller, P., Hausmann, M., and Cremer, C. (2008). SPDM: light microscopy with single-molecule resolution at the nanoscale. Appl. Phys. B *93*, 1–12.

Leslie, A.G.W. (2006). The integration of macromolecular diffraction data. Acta Crystallogr. D Biol. Crystallogr. *62*, 48–57.

Liu, H., Juo, Z.S., Shim, A.H.-R., Focia, P.J., Chen, X., Garcia, K.C., and He, X. (2010). Structural basis of semaphorin-plexin recognition and viral mimicry from Sema7A and A39R complexes with PlexinC1. Cell *142*, 749–761.

Marquardt, D.W. (1963). An Algorithm for Least-Squares Estimation of Nonlinear Parameters. SIAM J. Appl. Math. *11*, 431–441.

Mayo, C.J., Diprose, J.M., Walter, T.S., Berry, I.M., Wilson, J., Owens, R.J., Jones, E.Y., Harlos, K., Stuart, D.I., and Esnouf, R.M. (2005). Benefits of Automated Crystallization Plate Tracking, Imaging, and Analysis. Structure *13*, 175–182.

McCoy, A.J. (2007). Solving structures of protein complexes by molecular replacement with Phaser. Acta Crystallogr. D Biol. Crystallogr. *63*, 32–41.

McCoy, A.J., Grosse-Kunstleve, R.W., Adams, P.D., Winn, M.D., Storoni, L.C., and Read, R.J. (2007). Phasercrystallographic software. J. Appl. Crystallogr. *40*, 658–674.

Murshudov, G.N., Skubák, P., Lebedev, A.A., Pannu, N.S., Steiner, R.A., Nicholls, R.A., Winn, M.D., Long, F., and Vagin, A.A. (2011). REFMAC5 for the refinement of macromolecular crystal structures. Acta Crystallogr. D Biol. Crystallogr. *67*, 355–367.

Nicholls, R.A., Long, F., and Murshudov, G.N. (2012). Low-resolution refinement tools in REFMAC5. Acta Crystallogr. D Biol. Crystallogr. *68*, 404–417.

Niemann, H.H., Jäger, V., Butler, P.J.G., van den Heuvel, J., Schmidt, S., Ferraris, D., Gherardi, E., and Heinz, D.W. (2007). Structure of the Human Receptor Tyrosine Kinase Met in Complex with the Listeria Invasion Protein InlB. Cell *130*, 235–246.

O'Callaghan, C.A., Byford, M.F., Wyer, J.R., Willcox, B.E., Jakobsen, B.K., McMichael, A.J., and Bell, J.I. (1999). BirA Enzyme: Production and Application in the Study of Membrane Receptor–Ligand Interactions by Site-Specific Biotinylation. Anal. Biochem. *266*, 9–15.

Padilla-(, S., Audugé, N., Coppey-Moisan, M., and Tramier, M. (2008). Quantitative FRET Analysis by Fast Acquisition Time Domain FLIM at High Spatial Resolution in Living Cells. Biophys. J. *95*, 2976–2988.

Padilla-Parra, S., Audugé, N., Lalucque, H., Mevel, J.-C., Coppey-Moisan, M., and Tramier, M. (2009) Quantitative Comparison of Different Fluorescent Protein Couples for Fast FRET-FLIM Acquisition. Biophys. J. *97*, 2368–2376.

Padilla-Parra, S., Audugé, N., Tramier, M., and Coppey-Moisan, M. (2015). Time-domain fluorescence lifetime imaging microscopy: a quantitative method to follow transient protein-protein interactions in living cells. Cold Spring Harb. Protoc. *6*, 508–521.

Reeves, P.J., Callewaert, N., Contreras, R., and Khorana, H.G. (2002). Structure and function in rhodopsin: High-level expression of rhodopsin with restricted and homogeneous N-glycosylation by a tetracycline-inducible N-acetylglucosaminyltransferase I-negative HEK293S stable mammalian cell line. Proc. Natl. Acad. Sci. U.S.A. *99*, 13419–13424.

Rust, M.J., Bates, M., and Zhuang, X. (2006). Sub-diffraction-limit imaging by stochastic optical reconstruction microscopy (STORM). Nat. Meth. *3*, 793–796.

Seiradake, E., Schaupp, A., del Toro Ruiz, D., Kaufmann, R., Mitakidis, N., Harlos, K., Aricescu, A.R., Klein, R., and Jones, E.Y. (2013). Structurally encoded intraclass differences in EphA clusters drive distinct cell responses. Nat. Struct. Mol. Biol. *20*, 958–964.

Sievers, F., Wilm, A., Dineen, D., Gibson, T.J., Karplus, K., Li, W., Lopez, R., McWilliam, H., Remmert, M., Soding, J., et al. (2011). Fast, scalable generation of high-quality protein multiple sequence alignments using Clustal Omega. Mol. Syst. Biol. *7*, 539–539.

Smart, O.S., Womack, T.O., Flensburg, C., Keller, P., Paciorek, W., Sharff, A., Vonrhein, C., and Bricogne, G. (2012). Exploiting structure similarity in refinement: automated NCS and target-structure restraints in BUSTER. Acta Crystallogr. D Biol. Crystallogr. *68*, 368–380.

Tang, G., Peng, L., Baldwin, P.R., Mann, D.S., Jiang, W., Rees, I., and Ludtke, S.J. (2007). EMAN2: An extensible image processing suite for electron microscopy. J. Struct. Biol. *157*, 38–46.

van Heel, M., Harauz, G., Orlova, E.V., Schmidt, R., and Schatz, M. (1996). A New Generation of the IMAGIC Image Processing System. J. Struct. Biol., *116*, 17–24.

Walter, T.S., Diprose, J.M., Mayo, C.J., Siebold, C., Pickford, M.G., Carter, L., Sutton, G.C., Berrow, N.S., Brown, J., Berry, I.M., et al. (2005). A procedure for setting up high-throughput nanolitre crystallization experiments. Crystallization workflow for initial screening, automated storage, imaging and optimization. Acta Crystallogr. D Biol. Crystallogr. *61*, 651–657.

Walter, T.S., Meier, C., Assenberg, R., Au, K.-F., Ren, J., Verma, A., Nettleship, J.E., Owens, R.J., Stuart, D.I., and Grimes, J.M. (2006) Lysine Methylation as a Routine

Rescue Strategy for Protein Crystallization. Structure *14*, 1617–1622.

Yasuda, R. (2006). Imaging spatiotemporal dynamics of neuronal signaling using fluorescence resonance energy transfer and fluorescence lifetime imaging microscopy. Curr. Opin. Neurobiol. *16*, 551–561.

Collaborative Computational Project Number 4 (1994). The CCP4 suite: programs for protein crystallography. Acta Cryst. *D50,* 760-763.
