# Supplementary material for: Structural Basis for Plexin Activation and Regulation
Source: Neuron. 2016 Aug 3;91(3):548–60. doi: 10.1016/j.neuron.2016.06.018 (PMC4980550; doi:10.1016/j.neuron.2016.06.018)
Supplement: Document S2. Article plus Supplemental Information [file mmc2.pdf]

Article

Neuron

Structural Basis for Plexin Activation and Regulation

Highlights

- Structural studies reveal a major ring-like conformation for PlxnA ectodomains
- PlxnA ectodomains make head-to-stalk *cis*-interactions in vitro and on cell surface
- Disruption of PlxnA *cis*-interactions induces cell and growth cone collapse
- PlxnA ectodomain structure and interaction enable autoinhibition and activation

Authors

Youxin Kong, Bert J.C. Janssen, Tomas Malinauskas, ..., Sergi Padilla-Parra, R. Jeroen Pasterkamp, E. Yvonne Jones

Correspondence

r.j.pasterkamp@umcutrecht.nl (R.J.P.), yvonne@strubi.ox.ac.uk (E.Y.J.)

In Brief

PlxnA signaling, important in nervous system development and plasticity, requires multi-leveled regulation. Kong et al. reveal a novel mechanism for PlxnAs, in which autoinhibition pre- and activation post-ligand binding are achieved through distinct conformations and *cis*-interactions of the receptor ectodomains.

Accession Numbers

5L56
5L59
5L5C
5L7N
5L5G
5L74
5L5K
5L5L
5L5M
5L5N

Kong et al., 2016, Neuron *91*, 548–560
August 3, 2016 © 2016 The Authors. Published by Elsevier Inc.
http://dx.doi.org/10.1016/j.neuron.2016.06.018

Neuron
Article

Structural Basis for Plexin Activation and Regulation

Youxin Kong,[1,4] Bert J.C. Janssen,[1,5,4] Tomas Malinauskas,[1] Vamshidhar R. Vangoor,[2] Charlotte H. Coles,[1,6] Rainer Kaufmann,[1,3] Tao Ni,[1] Robert J.C. Gilbert,[1] Sergi Padilla-Parra,[1] R. Jeroen Pasterkamp,[2,*] and E. Yvonne Jones[1,*]
[1]Division of Structural Biology, Wellcome Trust Centre for Human Genetics, University of Oxford, Oxford, United Kingdom
[2]Department of Translational Neuroscience, Brain Center Rudolf Magnus, University Medical Center Utrecht, Utrecht, The Netherlands
[3]Department of Biochemistry, University of Oxford, Oxford, United Kingdom
[4]Co-first author
[5]Present address: Crystal and Structural Chemistry, Bijvoet Center for Biomolecular Research, Department of Chemistry, Faculty of Science, Utrecht University, Utrecht, The Netherlands
[6]Present address: Laboratory for Axon Growth and Regeneration, German Center for Neurodegenerative Diseases (DZNE), Bonn, Germany
*Correspondence: r.j.pasterkamp@umcutrecht.nl (R.J.P.), yvonne@strubi.ox.ac.uk (E.Y.J.)
http://dx.doi.org/10.1016/j.neuron.2016.06.018

SUMMARY

Class A plexins (PlxnAs) act as semaphorin receptors and control diverse aspects of nervous system development and plasticity, ranging from axon guidance and neuron migration to synaptic organization. PlxnA signaling requires cytoplasmic domain dimerization, but extracellular regulation and activation mechanisms remain unclear. Here we present crystal structures of PlxnA (PlxnA1, PlxnA2, and PlxnA4) full ectodomains. Domains 1–9 form a ring-like conformation from which the C-terminal domain 10 points away. All our PlxnA ectodomain structures show autoinhibitory, intermolecular "head-to-stalk" (domain 1 to domain 4-5) interactions, which are confirmed by biophysical assays, live cell fluorescence microscopy, and cell-based and neuronal growth cone collapse assays. This work reveals a 2-fold role of the PlxnA ectodomains: imposing a pre-signaling autoinhibitory separation for the cytoplasmic domains via intermolecular head-to-stalk interactions and supporting dimerization-based PlxnA activation upon ligand binding. More generally, our data identify a novel molecular mechanism for preventing premature activation of axon guidance receptors.

INTRODUCTION

The guidance of axons to their synaptic targets is an important event during neural circuit development. Axon guidance cues are also implicated in processes such as neuronal migration and polarization, neurite growth and pruning, and synaptogenesis (Kolodkin and Tessier-Lavigne, 2011; Bashaw and Klein, 2010; Yogev and Shen, 2014). Cell surface receptors at the leading tips of growing neurites or migrating cells function to detect axon guidance cues and to elicit a cellular response. Different families of guidance receptors have been identified, but our understanding of how these receptors signal or how their activity is regulated is very incomplete. For example, receptor pre-clustering prior to ligand binding may be important for the spatial and temporal regulation of signaling. However, the molecular mechanisms by which receptor activation is achieved and ligand-independent activation avoided are for many systems still obscure (Atanasova and Whitty, 2012).

Semaphorins form the largest family of secreted and membrane-anchored axon guidance cues and generally require plexins as cell surface receptors in neurons (Kolodkin and Tessier-Lavigne, 2011; Pasterkamp, 2012; Jongbloets and Pasterkamp, 2014). Plexins are large type 1 transmembrane proteins comprising typically ten domains in their extracellular segment, a single membrane spanning region that is predicted to be helical and a conserved, cytoplasmic region that contains a GTPase-activating protein (GAP) domain with a Rho GTPase-binding domain insert (Figure 1A). Plexins are subdivided into four classes (A to D) based on sequence similarity. Class A plexins (PlxnAs) are the best-characterized and act as neuronal receptors for secreted class 3 (Sema3s), as well as transmembrane class 5 and class 6 (Sema5s and Sema6s) semaphorins (Pasterkamp, 2012).

The largest portion of plexin ectodomain structurally characterized to date, $PlxnA2_{1-4}$, comprises the ligand-binding sema domain, followed by a PSI, IPT, and a second PSI domain (Janssen et al., 2010). The mechanism of semaphorin–PlxnA recognition has been uncovered by crystal structures of PlxnA ectodomain fragments in complex with their cognate semaphorins (Janssen et al., 2010; Liu et al., 2010; Nogi et al., 2010). In isolation, the semaphorin ligand is characteristically a dimer (Siebold and Jones, 2013). In semaphorin–plexin structures each subunit of a semaphorin dimer binds a monomeric plexin sema domain (Janssen et al., 2010, 2012; Liu et al., 2010; Nogi et al., 2010). These 2:2 semaphorin–plexin complexes indicate a bivalent binding mode is required to activate plexins, a mechanism supported by in solution and cell-based assays (Janssen et al., 2010; Nogi et al., 2010). Sema3s require neuropilins to cross-brace the Sema3–PlxnA complex, but the generic, 2:2 plexin–semaphorin interaction, mediated by the sema domains

 © 2016 The Authors. Published by Elsevier Inc.
This is an open access article under the CC BY license (http://creativecommons.org/licenses/by/4.0/).

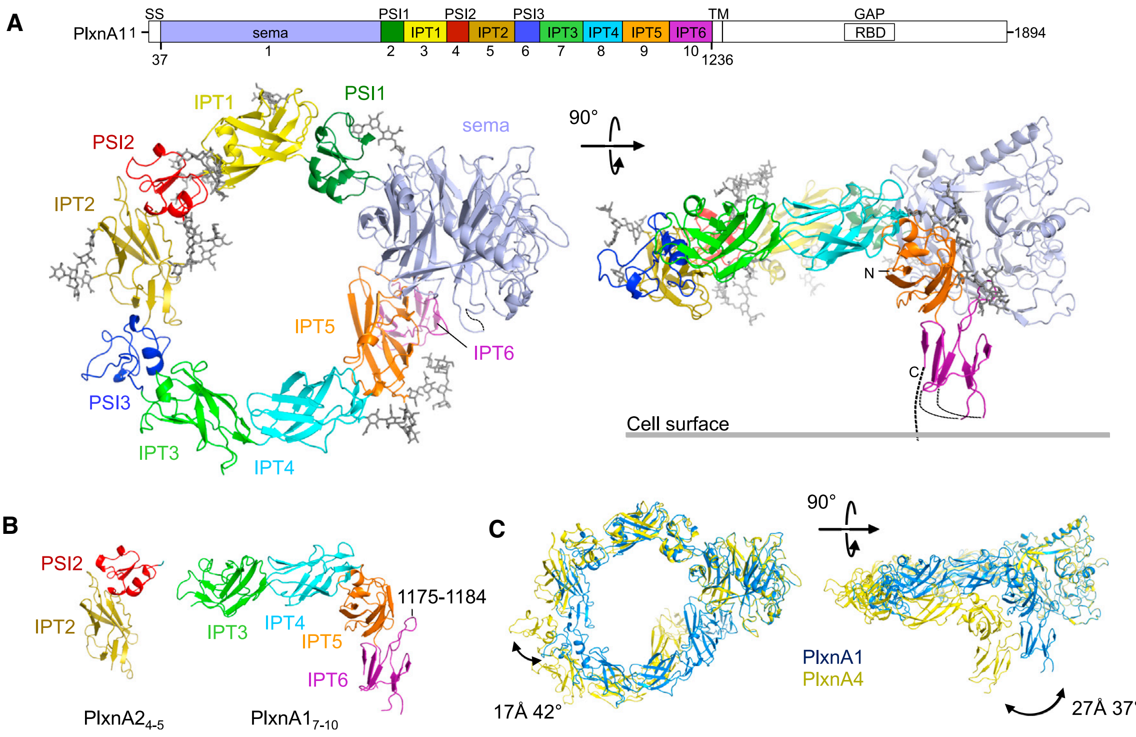

Figure 1. Crystal Structures of PlxnAs Extracellular Segments

(A) Schematic domain organization of PlxnA1 (top). SS, signal sequence; sema, semaphorin; PSI, plexin-semaphorin-integrin; IPT, Ig domain shared by plexins and transcription factors; TM, transmembrane region; GAP, GTPase activating protein; RBD, Rho GTPase-binding domain. The domains included in the crystallization constructs are colored. Ribbon representation of PlxnA1$_{1\text{-}10}$ at 4 Å resolution in top and side views (left and right, respectively). A gray line indicates how PlxnA1 is attached to the cell surface (right). N-linked glycans are shown in gray stick representation.

(B) Ribbon representation of PlxnA2$_{4\text{-}5}$ at 1.36 Å resolution (left) and of PlxnA1$_{7\text{-}10}$ at 2.2 Å resolution (right). Loop 1,175–1,184 involved in contacts with domain IPT5 is indicated.

(C) Ribbon representation of PlxnA1$_{1\text{-}10}$ and PlxnA4$_{1\text{-}10}$ superposed on the basis of the sema domain (views as in [A]). Differences in PSI3 and IPT6 domain orientation (left and right, respectively) are indicated by domain translation and rotation. See also Figure S1 and Table S1.

of both binding partners, is conserved (Janssen et al., 2012). Dimerization of the plexin cytoplasmic region is a pre-requisite for activation (Wang et al., 2012) and crystal structures of engineered cytoplasmic region dimers support a model in which the bivalent binding of the semaphorin ligand to two plexin ectodomains drives the juxtaposition of their cytoplasmic regions with consequent dimerization and activation (Wang et al., 2013). Interestingly, functional and fluorescent light microscopy analyses have also suggested that the PlxnA ectodomain plays an essential role in autoinhibition of signaling prior to semaphorin binding (Takahashi and Strittmatter, 2001; Marita et al., 2015). To establish how semaphorin binding is coupled to cytoplasmic domain dimerization and how the PlxnA ectodomain provides autoinhibition pre-ligand binding, structural data are required on the full extracellular PlxnA segment.

In this study, we first detail a distinctive ring-like architecture that is common to the full-length (ten domain) extracellular segments of PlxnA1, PlxnA2, and PlxnA4. Negative stain electron microscopy (EM) confirms this ring-like structure as the predominant form (alongside a minor twisted-open, chair-like conformation). Our crystal structures also reveal an intriguing intermolecular "head-to-stalk" interaction common to all PlxnAs. We confirm this interface mediates PlxnA–PlxnA interactions and occurs pre-semaphorin binding using structure-guided mutagenesis, biophysical assays, and fluorescence microscopy-based live cell imaging. Functional assays (i.e., COS-7 cell-based and dentate gyrus [DG] granule cell growth cone collapse experiments) confirm that intermolecular *cis*-interactions of the PlxnA ectodomains impose pre-signaling autoinhibition. Based on these results, we propose that, before signaling, PlxnAs exist as pre-formed dimers in which the association between the ring-like PlxnA ectodomains maintains a separation of their cytoplasmic domains. This "outside-together-inside-apart" mechanism keeps PlxnA signaling in check in the absence of ligands while, possibly, allowing co-localization of receptors to facilitate rapid signaling in response to ligand binding.

RESULTS

Crystal Structures of Full Extracellular Segments of PlxnAs Reveal Ring-Like Conformation

We determined crystal structures of the full extracellular segments of mouse PlxnA1, PlxnA2, and PlxnA4 from eight crystal forms providing a total of 13 independent structures that range in maximum resolution from 4 to 10 Å (Figures 1 and S1; Table S1). The 4 Å dataset was obtained by dehydrating crystals of

PlxnA1$_{1\text{-}10}$ that diffracted otherwise at best to 6 Å resolution (see Experimental Procedures). Structure determination of the full extracellular segments was aided by the available structures of PlxnA2$_{1\text{-}4}$ (Janssen et al., 2010) (PDB: 3OKT), PlxnA2$_{1\text{-}2}$ (Nogi et al., 2010) (PDB: 3AL9), and additional higher resolution crystal structures that we determined of portions of PlxnA1 and PlxnA2; that is, PlxnA2$_{4\text{-}5}$ to 1.36 Å resolution and PlxnA1$_{7\text{-}10}$ to 2.2 Å resolution (Figure 1B). In five out of 13 full extracellular structures all ten domains are resolved whereas in the other eight structures some of the C-terminal domains were not modeled due to fragmentary or absent electron density. This indicates that some flexibility is present in the extracellular segment of plexins.

In the crystal structure of PlxnA1-full ectodomain, the sema domain (domain 1, "head") together with domains 2–9 ("stalk") lie in a 230 Å long curve that forms a ring-like conformation (Figures 1A and S1A). All consecutive domains are arranged in a beads-on-a-string-like fashion, with short interdomain linkers (up to two residues) and extensive interfaces (buried surface area ranging from 716 to 1,172 Å^2), each interdomain interface having a small hydrophobic core. These substantial domain–domain interactions likely contribute to conservation of the overall curved ring-like structure among the different PlxnA members and crystal forms. Some flexibility in the stalk is evident on comparison of the 13 different structures and contributes to the ring diameter ranging from 84 Å to 99 Å (Figures 1C and S1A). The interface between PSI3 and IPT3 (domains 6 and 7) differs markedly from those of PSI1-IPT1 and PSI2-IPT2, imposing a more acute angle on the relative arrangement of these two domains, and thus reducing the ring circumference (Figure S1B). The ring is closed only in the 4 Å structure of PlxnA1$_{1\text{-}10}$, by interactions between the sema domain and domain IPT5 (domain 9). This relative compaction can, most likely, be attributed to the dehydration procedure applied to generate this crystal form. In all other structures, the plexin extracellular domains associate only via the consecutive domain-domain interactions. Thus, contrary to previous speculations on plexin ectodomain structure (Antipenko et al., 2003; Janssen et al., 2010; Takahashi and Strittmatter, 2001), in the unliganded state the sema domain does not make any intramolecular interactions with the remainder of the plexin other than to the directly ajoining PSI1 domain.

The membrane proximal domain IPT6 (domain 10) is positioned perpendicular to the ring formed by domains 1 to 9, and the IPT6 C terminus points away from the plane of the ring. This out-of-the-ring orientation of IPT6 is observed in all five PlxnA ectodomain structures for which the electron density is sufficiently well ordered to allow IPT6 to be resolved. The same relative domain orientation recurs in the structure of PlxnA1$_{7\text{-}10}$. Possibly this distinctive orientation is stabilized by a loop (1,175–1,184) extending from IPT6 that provides hydrophobic interactions with three residues in domain IPT5, Trp1055, Ile1057, and Tyr1145 (Figures 1B and S1C), which are conserved across the A class Plxns. In the full-length plexin, the linker between IPT6 and the transmembrane helix is predicted to be short (6 residues for PlxnA1), and thus, for this conformation, the plexin extracellular segment is most likely positioned close to the membrane with its ring parallel to the cell surface.

Negative Stain EM Supports a Major Ring-Like Conformation for PlxnA1$_{1\text{-}10}$

To assess the possible conformations of PlxnA full extracellular segments when removed from the constraints of crystal packing, we analyzed the structure of PlxnA1$_{1\text{-}10}$ with negative stain EM (Figures 2 and S2). In the electron micrographs, PlxnA1$_{1\text{-}10}$ is monomeric (Figure S2A). Assessed by eye, the majority of PlxnA1$_{1\text{-}10}$ molecules appeared to orient with the flat face of the ring lying on the grid in a conformation resembling the crystal structures, though a proportion adopted a twisted-open conformation (Figure S2A). In agreement with this initial assessment, the class averages of PlxnA1$_{1\text{-}10}$ sorted into two categories: "ring-like" or "chair-like" (twisted-open) conformations (Figures 2, S2B, and S2C). The ring-like species, which includes 66.0% of the particles, essentially matches the PlxnA$_{1\text{-}10}$ crystal structures. A bulky feature at one end of the ring is readily identifiable as the sema domain (flat or sideways orientations on the grid resulting in a disk-like or bulb-like projection, respectively). From the sema domain onward, the stalk region extends in a range of curved conformations, resulting, as in the crystal structures, in some variation in ring circumference. A second species, which includes 30.5% of the particles, can be identified by its distinct chair-like conformation. The N-terminal half of the stalk region curves out from the sema domain as in the ring-like structures, but the C-terminal half appears twisted by a 180° flip so that it curves away rather than completing the ring. No clear class averages could be generated for the remaining 3.5% of the particles. The PlxnA$_{1\text{-}10}$ crystal structures could be fitted to the class averages by assuming two flexion points, one between domains 1 and 2 (sema–PSI1) and another at the domain 5–domain 6 (IPT3–PSI3) junction (see Figure S2 and Supplemental Experimental Procedures). The excellent agreement between the class averages and projections of the structural models generated by rotations around these two potential flexion points reinforces the notion that the ten domain extracellular segments of PlxnAs have relatively limited flexibility (Figures 2 and S2C). Our results indicate that PlxnA ectodomains exist in only two alternative conformations with limited interdomain flexibility: the preferred, ring-like conformation as observed in crystal structures and the less frequent, chair-like conformation.

PlxnA Crystal Structures Reveal an Intermolecular Interface between the Sema and PSI2-IPT2 Domains

All our PlxnA full ectodomain crystal structures have an intermolecular plexin–plexin interface in common (Figures 3 and S3A). This interface is formed by blades 1–3 of the sema domain of one plexin molecule and the outer side of domains 4-5 (PSI2-IPT2) from the ring of a second plexin (Figure 3A). In the 4 Å PlxnA1$_{1\text{-}10}$ structure, the interface has a buried surface area of 1,303 Å^2 (355 Å^2 between sema–PSI2 and 1,008 Å^2 between sema–IPT2) and a complementary electrostatic charge distribution (Figure 3B). The residues in this interface are conserved among vertebrate PlxnAs but much less among all plexins (Figure 3B), suggesting that this plexin–plexin interaction may be an important functional feature of PlxnAs. This finding raises the possibility that PlxnA molecules on the same cell

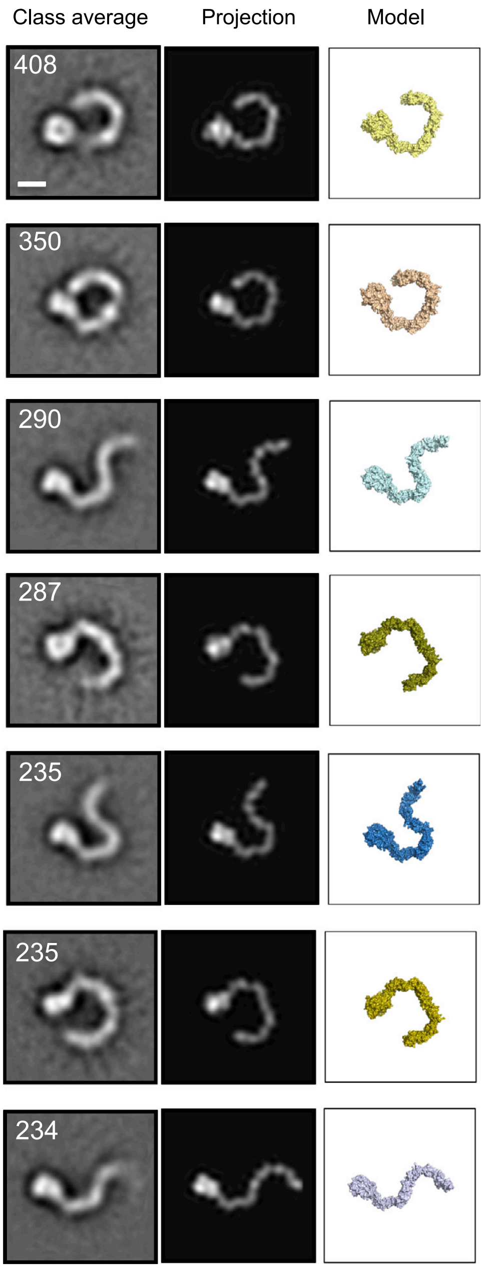

Figure 2. Negative Stain EM Reveals Two Alternative Conformations of PlxnA1$_{1\text{-}10}$

Negative stain EM class averages of PlxnA1$_{1\text{-}10}$ correlate well with 2D projections of the crystal-structure-based models. Seven representative class averages of PlxnA1$_{1\text{-}10}$ are shown alongside the correlated projections and corresponding structural models (colored according to models shown in Figure S2B). The number of particles within each class is listed on the top left corner. The projections were generated from the 6 Å PlxnA1$_{1\text{-}10}$ crystal structure of space group P2$_1$ (see Figure S1A) filtered to a Gaussian resolution of 10 Å. Scale bar, 5 nm. See also Figure S2.

surface can interact in *cis* in a "head-to-stalk" manner using the sema–PSI2-IPT2 intermolecular interface to form dimers or small oligomeric clusters. A simple model-building exercise confirmed this possibility (Figures 3C and S3B). Taking into account the tilt of 50 degrees between the rings of two interacting PlxnAs, and the flexibility in the stalk region, PlxnA dimers can be accommodated on the cell surface (Figure 3C), but arrays incorporating more than three molecules clash with the plasma membrane in the absence of substantial membrane curvature (Figure S3B). Thus, our structural data raise the possibility that the ring-like PlxnA ectodomains exist as "head-to-stalk" dimers on the cell surface.

In Solution Assays Confirm the Intermolecular Head-to-Stalk Interactions between PlxnA Ectodomains

Intermolecular interaction between full plexin ectodomains has been shown previously by us for PlxnA2 (Janssen et al., 2010) and by others for PlxnC1 (Liu et al., 2010), but the basis for this association remained unclear. In light of our PlxnA ectodomain structures, we set out to assess whether PlxnAs in solution interact via the sema–PSI2-IPT2 interface. The relatively low resolution of our full-length ectodomain structures mean that the residue-to-residue level details of the interface interactions must be interpreted with caution; we therefore chose to test the sema–PSI2-IPT2 interface by introducing glycosylation sites rather than simple point mutations. We first found that disrupting the sema–PSI2-IPT2 interface by inserting a bulky, N-linked glycan onto either the sema domain (PlxnA1$_{1\text{-}10}$ F145N/L147S) or the PSI2 domain (PlxnA1$_{1\text{-}10}$ F693N/E695S) can block the PlxnA1–PlxnA1 interaction using multi-angle light scattering (MALS) experiments coupled to size-exclusion chromatography (Figure S4). We then used analytical ultracentrifugation (AUC) sedimentation velocity experiments to demonstrate that in solution PlxnA1$_{1\text{-}10}$ exists in heterogeneous states encompassing monomers up to tetramers arising from intermolecular sema–IPT2-PSI2 interactions (Figure 4A). At a high concentration of 28.1 μM (4 mg/ml), PlxnA1$_{1\text{-}10}$ gave four distinct peaks with sedimentation coefficients corresponding to monomer, dimer, trimer, and tetramer species, respectively (Figure 4AI). When the concentration was decreased to 7.0 μM (1 mg/ml), the population of PlxnA1$_{1\text{-}10}$ dimer and higher order oligomers decreased, and most of the PlxnA1$_{1\text{-}10}$ remained monomeric (consistent with the monomeric PlxnA1$_{1\text{-}10}$ seen at concentrations of 7 to 33 nM in the EM studies; Figure S2 and Experimental Procedures). The discrete shape of the peaks is indicative of relatively slow exchange between the monomeric and oligomeric states, and the sedimentation coefficients predicted by the structural models for the different oligomeric states fall within the spectrum of each peak. There was no evidence of plexin oligomerization states larger than a tetramer, consistent with the relatively weak intermolecular interactions of PlxnA1$_{1\text{-}10}$ being insufficient to build up large oligomers. In order to assess directly the contribution of sema–IPT2-PSI2 interactions, we also analyzed the behavior of PlxnA1$_{1\text{-}10}$ F693N/E695S during AUC. This putative interaction-blocking mutant showed one major sedimentation coefficient peak corresponding to the monomer species at both 28.1 μM and 7.0 μM (Figure 4AII).

We then undertook surface plasmon resonance (SPR) binding equilibrium experiments to probe the sema–IPT2-PSI2 interface and determine the affinity of interaction. We engineered

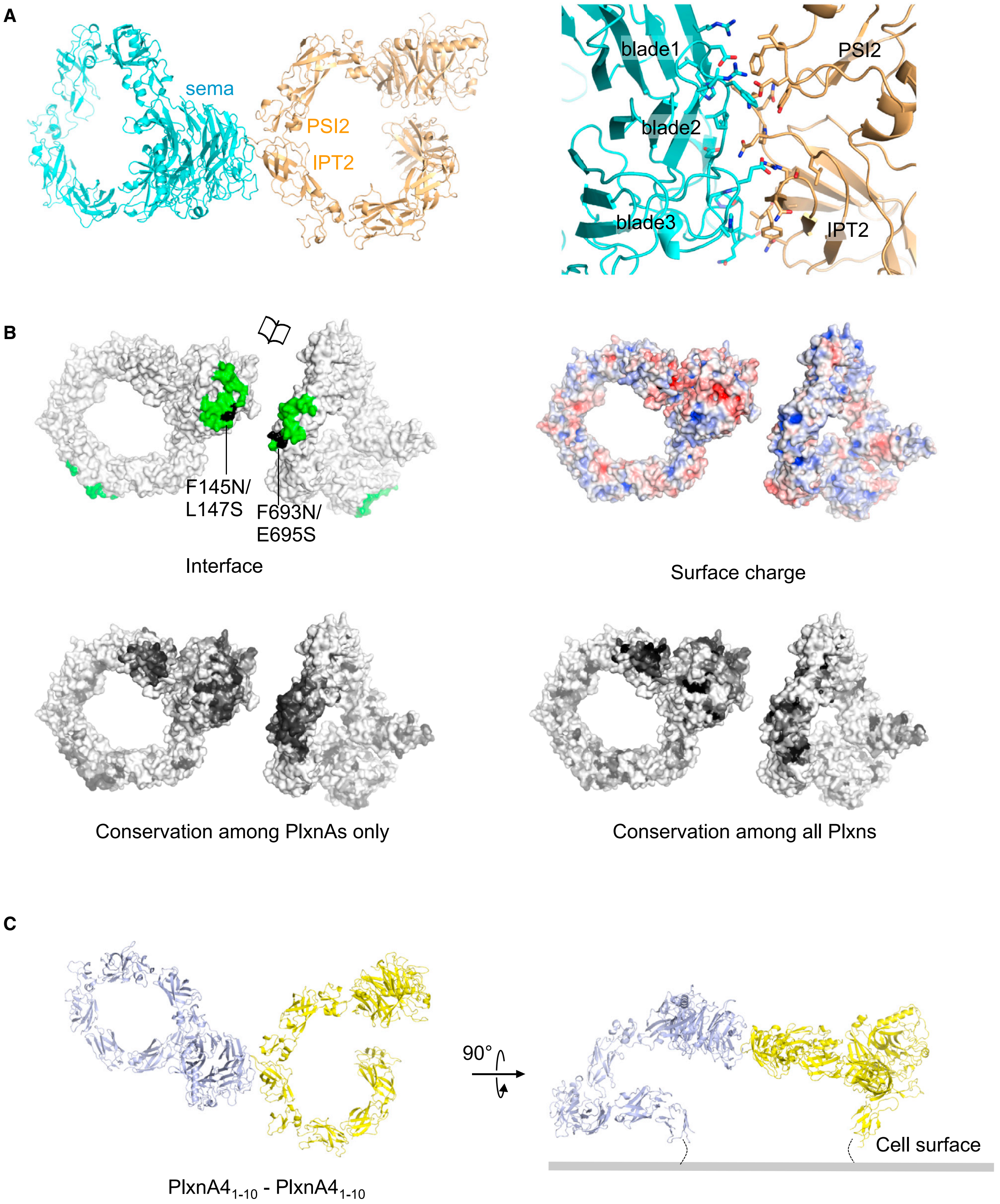

Figure 3. PlxnA Intermolecular Interface between the Sema Domain and Domains PSI2-IPT2

(A) Ribbon representation of the PlxnA1$_{1\text{-}10}$ intermolecular interaction in the 4 Å crystal structure (left) and enlargement with interface residues shown in stick representation (right).

(legend continued on next page)

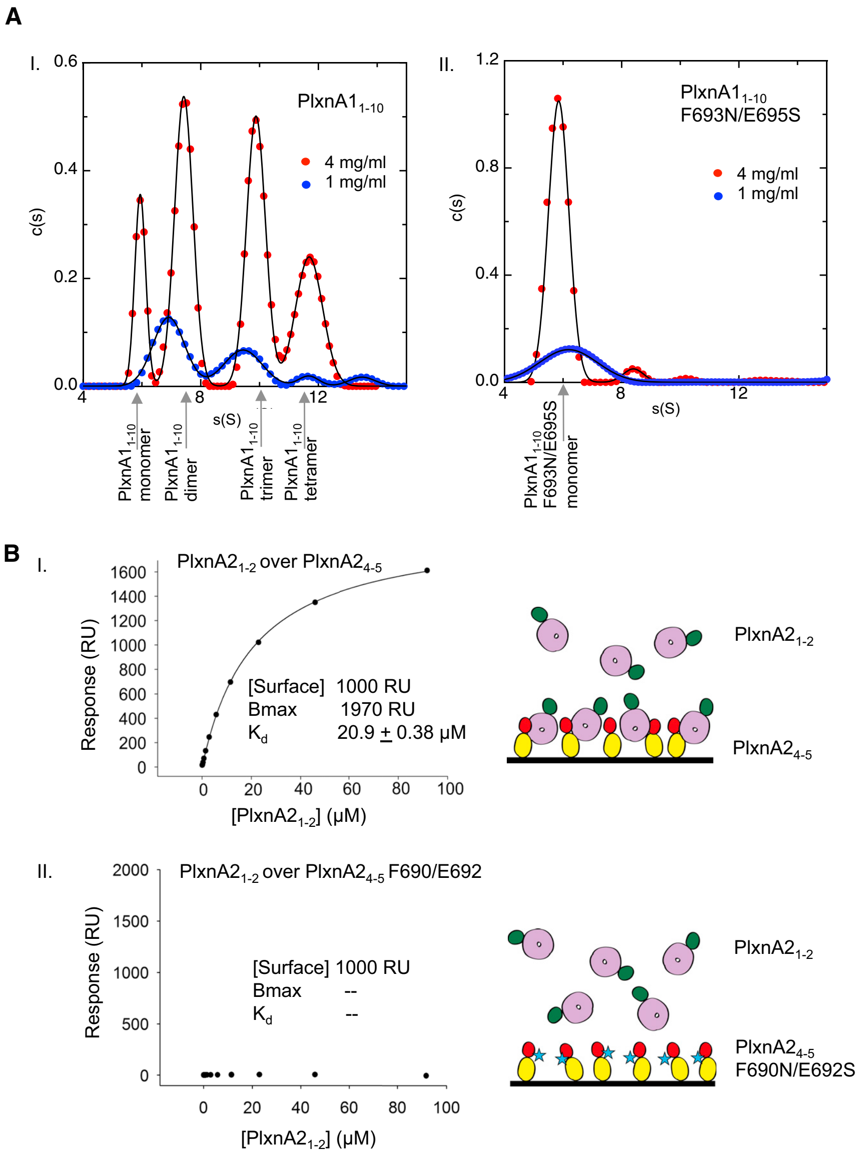

Figure 4. AUC and SPR Experiments Reveal PlxnA–PlxnA interactions via the Sema–PSI2-IPT2 Interface

(A) Distribution of sedimentation coefficients of PlxnA1$_{1-10}$ (I) and PlxnA1$_{1-10}$ F693N/E695S (II) at different concentrations measured by AUC sedimentation velocity experiments. Calculated from the Lamm equation model, c(s) (in a.u.) is plotted against the sedimentation coefficient, S (in svedbergs). The predicted sedimentation coefficients of different oligomeric states that best correspond to the peak values are indicated by arrows. For PlxnA1$_{1-10}$ F693N/E695S 28.1 μM (4 mg/ml) minor dimer and trimer peaks potentially result from other weak or unspecific intermolecular interactions.

(B) PlxnA2$_{1-2}$ bound to PlxnA2$_{4-5}$ with a K$_d$ of 20.9 ± 0.38 μM (I), while PlxnA2$_{4-5}$ F690N/E692S completely abolished binding with PlxnA2$_{1-2}$ (II) revealed by SPR equilibrium experiments. In the illustrations (right), the sema domain, PSI1 domain, PSI2 domain, and IPT2 domain are colored in purple, green, red, and yellow, respectively. The N-linked glycans introduced by the F690N/E692S mutation are represented as blue stars.

two separate segments consisting of the PlxnA2 sema-PSI1 domains (PlxnA2$_{1-2}$) and the PSI2-IPT2 domains (PlxnA2$_{4-5}$); each segment encompasses one side of the putative "head-to-stalk" intermolecular interface. We found that PlxnA2$_{1-2}$ and PlxnA2$_{4-5}$ associated with an equilibrium dissociation constant (K$_d$) of 20.9 ± 0.38 μM (Figure 4BI). The introduction of glycosylation by mutation F690N/E692S in PlxnA2$_{4-5}$ completely abrogated the interaction with PlxnA2$_{1-2}$ (Figure 4BII), consistent with our MALS and AUC results for the equivalent mutation in PlxnA1$_{1-10}$ (PlxnA1$_{1-10}$ F693N/E695S). Taken together, the results of our in solution assays point to a head-to-stalk mode of interaction between receptor ectodomains that is common to all PlxnAs.

FRET-FLIM Confirms PlxnA–PlxnA Interactions via the Sema–PSI2-IPT2 Interface on the Cell Surface

We next sought information on the oligomeric state of PlxnAs at the cell surface. Our first experiments, using localization microscopy, suggested that PlxnAs form only dimers or small oligomers (Figure S5A). As the size of these structures was too close to the resolution limit (~50 nm) of localization microscopy, we turned to Förster resonance energy transfer measured by fluorescence lifetime imaging microscopy (FRET-FLIM), a method suited to measure transient and low-affinity interactions (Padilla-Parra et al., 2008, 2015; Yamada et al., 2009; Zhao et al., 2014) (Figures 5 and S5B). Fluorescent proteins mTFP1 and mVenus were fused to the PlxnA2 extracellular-transmembrane segment via a RTLEVLFQGP linker to provide the FRET donor (PlxnA2-mTFP1) and acceptor (PlxnA2-mVenus), respectively. The linker

(B) Surface representation of an opened view of two PlxnA1$_{1-10}$ molecules with the sema–PSI2-IPT2 interface in green and interface mutants in black (top left) and electrostatic potential from red (−6 k$_b$T/e$_c$) to blue (6 k$_b$T/e$_c$) (top right). PlxnA1$_{1-10}$ is color coded according to residue conservation (from nonconserved, white, to conserved, black) on the basis of alignments containing vertebrate PlxnA sequences only (bottom left) or Plxn sequences from all classes (bottom right). The structure of domain IPT6 has several unmodelled loops (see Experimental Procedures) and hence is revealing its conserved hydrophobic core in these views. See also Figure S3.

(C) Possible cell surface orientation of PlxnA dimers formed via the sema–PSI2-IPT2 interface based on the conformation and packing of the PlxnA4$_{1-10}$ crystal structure (space group P4$_1$).

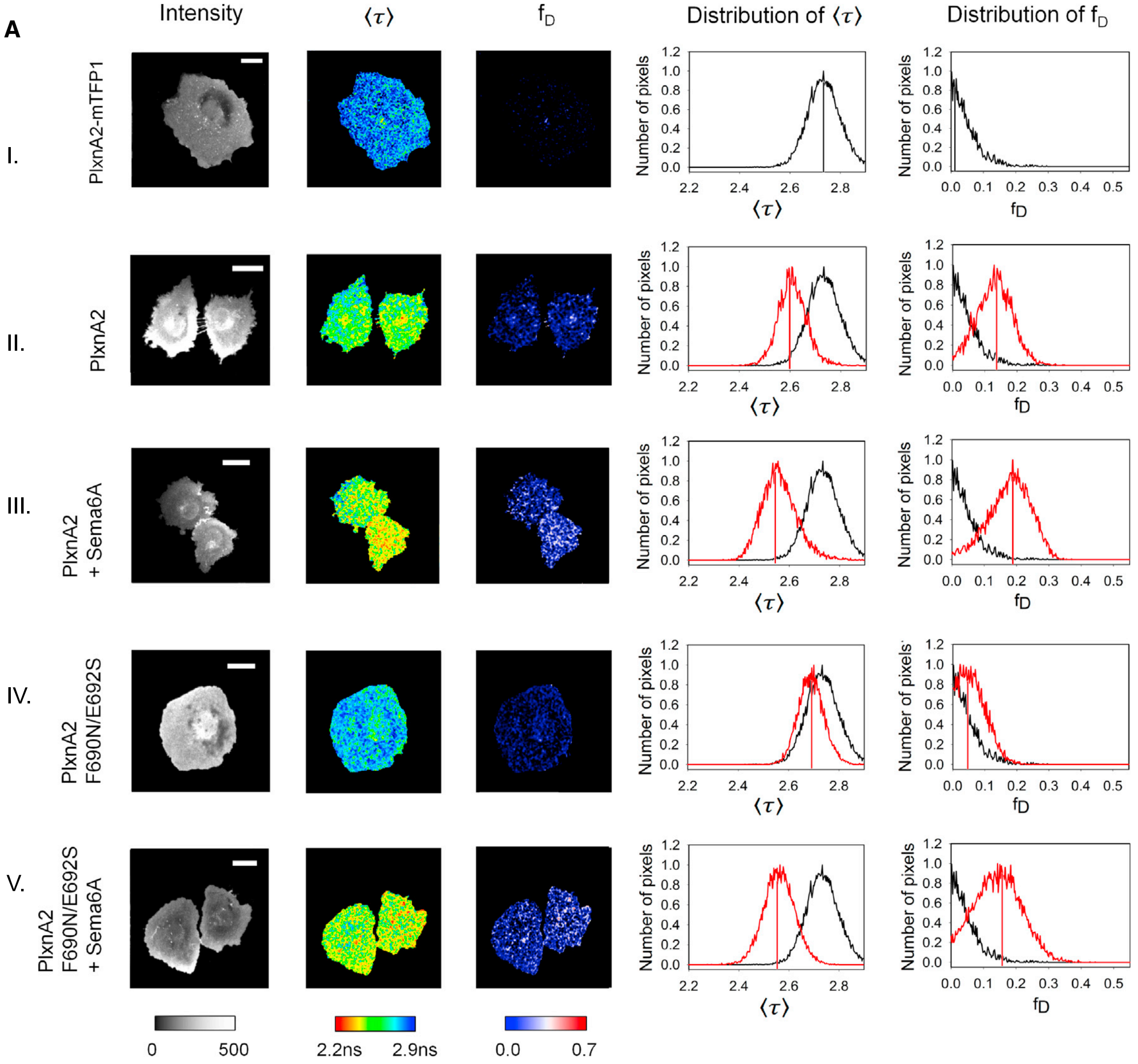

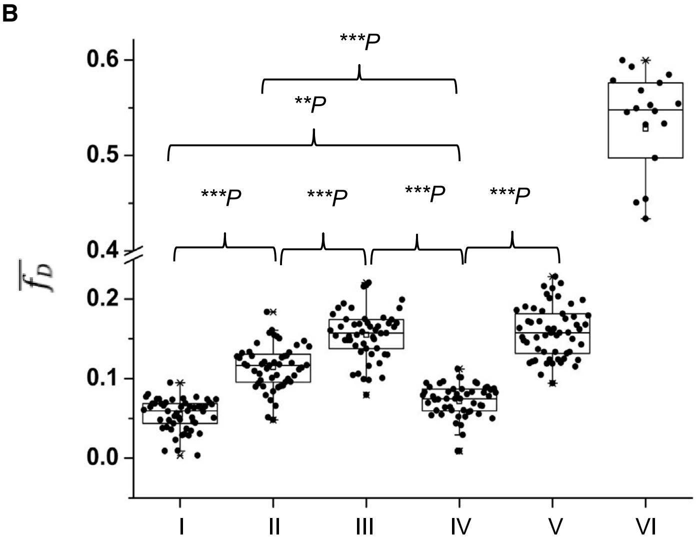

	Condition	$\overline{f_D}$
I	PlxnA2-mTFP1	0.05 ± 0.02
II	PlxnA2	0.11 ± 0.02
III	PlxnA2 + Sema6A	0.16 ± 0.03
IV	PlxnA2 F690N/E692S	0.07 ± 0.02
V	PlxnA2 F690N/E692S + Sema6A	0.16 ± 0.03
VI	mTFP1-mVenus Tandem	0.56 ± 0.07

(legend on next page)

was designed to allow FRET to occur if PlxnA2-mTFP1 and mPlxnA2-mVenus interact via the sema–IPT2-PSI2 interface, or through ligand binding (Figure S5BI).

For each FLIM image of a live COS-7 cell, intermolecular interactions were quantified by the mean fluorescence lifetime, $\langle\tau\rangle$, and the fraction of interacting donor, f_D, on a pixel-by-pixel basis (Figure S5BII; see also Supplemental Experimental Procedures) (Padilla-Parra et al., 2015). Average mean lifetime ($\overline{\langle\tau\rangle}$) and average fraction of interacting donors ($\overline{f_D}$) were calculated from these pixel-by-pixel distributions (Figure 5). To exclude potential artifacts due to protein overexpression, we only analyzed cells expressing similar PlxnA levels (assessed by fluorescence intensity). The ratio between FRET donor-fused PlxnAs and acceptor-fused PlxnAs did not correlate with the level of FRET (i.e., extent of PlxnA–PlxnA interactions) (Figure S5BII). A representative cell expressing PlxnA2-mTFP1 alone (no-FRET) has a $\overline{\langle\tau\rangle}$ of 2.73 ns and an $\overline{f_D}$ of 0.05 (Figure 5AI). In contrast, a cell co-expressing PlxnA2-mTFP1 and PlxnA2-mVenus shows a $\overline{\langle\tau\rangle}$ of 2.61 ns and an $\overline{f_D}$ of 0.11, indicating FRET is occurring (Figure 5AII). A cell co-expressing the putative interaction-inhibiting mutants PlxnA2 F690N/E692S-mTFP1 and PlxnA2 F690N/E692S-mVenus exhibits much reduced FRET, with a longer $\overline{\langle\tau\rangle}$ (2.65 ns) and a lower $\overline{f_D}$ (0.07) (Figure 5AIV). These results demonstrate that the sema–PSI2-IPT2 interface indeed mediates PlxnAs *cis*-interactions at the cell surface (for a sample size of >52 cells, $p < 0.001$; Figures 5BI, 5BII, and 5BIV). After Sema6A stimulation, a representative cell co-expressing PlxnA2-mTFP1 and PlxnA2-mVenus showed a $\overline{\langle\tau\rangle}$ of 2.55 ns and an $\overline{f_D}$ of 0.16 (Figure 5AIII). Similarly, a cell co-expressing PlxnA2 F690N/E692S-mTFP1 and PlxnA2 F690N/E692S-mVenus showed a level of FRET as high as the wild-type PlxnA2 ($\overline{\langle\tau\rangle} = 2.54$ ns and $\overline{f_D} = 0.16$) (Figure 5AV). These results (identical values of $\overline{f_D} = 0.16 \pm 0.03$ for >52 cells; Figures 5BIII and 5BV) point to increased dimerization of both wild-type and mutant PlxnA2s on ligand binding. In sum, our fluorescent microscopy-based experiments show compelling evidence for PlxnA *cis*-interactions mediated by the sema–PSI2-IPT2 interface occurring on the cell surface. Semaphorin binding to PlxnAs induces higher levels of interaction, which is most likely independent from the *cis*-interaction-modulated PlxnA pre-association.

PlxnAs Are Autoinhibited by Head-to-Stalk *cis*-Interactions

The PlxnA head-to-stalk *cis*-interactions that we observed using various approaches could function to impose pre-signaling autoinhibition. To test this model at the functional level, we first used COS-7 cells as a heterologous system that mimics growth cone collapse (Takahashi and Strittmatter, 2001). As reported previously, COS-7 cells expressing full-length, wild-type PlxnA4 (PlxnA4$_{FL}$) displayed a normal, spread-out morphology (Figure 6A) (Suto et al., 2003). In contrast, expression of PlxnA4$_{FL}$ F689N/E691S in which the sema–PSI2-IPT2 interface is blocked by an N-linked glycan (analogous to that used previously in PlxnA1 and PlxnA2) results in robust cell contraction (Figure 6A). The extent of cell contraction is similar to that induced by PlxnA4$_{\Delta ecto}$, a constitutively active mutant lacking the entire extracellular region (Figure 6B).

To further probe whether *cis*-interactions between PlxnA ectodomains mediate autoinhibitory interactions, dentate gyrus (DG) granule cells from the hippocampus were incubated with wild-type PlxnA1$_{4-5}$ (domains PSI2-IPT2) or its interaction-blocking mutant, PlxnA1$_{4-5}$ F693N/E695S (Figures 6C and S6). PlxnA1$_{4-5}$ (final concentration 200 μM, 10-fold the K_d of PlxnA2$_{4-5}$–PlxnA2$_{1-2}$ interactions measured by SPR) was predicted to bind full-length PlxnA molecules on the growth cone surface, thereby counteracting *cis*-interactions between PlxnA molecules (PlxnA1 but also other PlxnAs expressed by DG granule cells) and leading to receptor activation; DG granule cells were used because they endogenously express various PlxnAs and are responsive to a wide range of PlxnA ligands, including Sema3s and Sema6s (e.g., Suto et al., 2007). Following treatment with vehicle, growth cones of DG granule cells showed a typical fan-shaped morphology (Figure 6C). In contrast, incubation with PlxnA1$_{4-5}$, but not PlxnA1$_{4-5}$ F693N/E695S, induced marked growth cone collapse (Figure 6C). These qualitative observations were confirmed by a detailed analysis of growth cone morphology (Figure 6D). Growth cone collapse was graded using a scale from one (uncollapsed) to ten (fully collapsed) according to a matrix of growth cones with different morphologies, allowing the detection of even subtle changes in growth cone morphology (Figure S6) (van Erp et al., 2015). This analysis revealed that whereas PlxnA1$_{4-5}$ induced robust collapse, this effect was not observed when using a construct unable to bind endogenous PlxnAs, PlxnA1$_{4-5}$ F693N/E695S (Figures 6C and 6D). These results coupled with our observations in COS-7 cell contraction assays support a model in which PlxnAs are normally autoinhibited by intermolecular *cis*-interactions. Disruption of these interactions, by introducing mutations or addition of competitive protein domains, induces constitutive signaling and collapse.

DISCUSSION

The construction of a functional neuronal network by axon guidance cues requires multi-level and spatiotemporal regulation of

Figure 5. FRET-FLIM in Live COS-7 Cells Indicates Cell Surface PlxnA-PlxnA Head-to-Stalk *cis*-Interactions Occur Pre-Ligand Binding

(A) Pixel-by-pixel analysis of representative cells expressing PlxnA2-mTFP1 alone (I), PlxnA2-mTFP1 and PlxnA2-mVenus before (II) and after (III) Sema6A stimulation, and PlxnA2 F690N/E692S-mTFP1 and PlxnA2 F690N/E692S-mVenus before (IV) and after (V) Sema6A stimulation. From left to right, the images show the fluorescence intensity (photon count), $\langle\tau\rangle$, and f_D in pseudo-color scales. The histograms display the corresponding distribution of $\langle\tau\rangle$ and f_D (black: PlxnA2-mTFP1 expressed alone; red: mTFP1- and mVenus-fused PlxnA2 or PlxnA2 F690N/E692S coexpressed). The lines within the peaks indicate average values $\overline{\langle\tau\rangle}$ and $\overline{f_D}$. Scale bar, 10 μm.

(B) The distribution of $\overline{f_D}$ for large populations of cells (N > 52) expressing fluorescent-protein-fused PlxnA2s (I to V) and a high-FRET mTFP-mVenus Tandem (VI; Padilla-Parra et al., 2008). The upper and lower quartiles of each sample are represented by the upper and lower sides of the boxes; the medians are represented by the black lines, and the means by hollow points. The range of the whiskers indicates the statistical outliers with a coefficient 1.5. The p values signify the statistical significances between two selected samples determined by paired two-sample t tests. The values of $\overline{f_D}$ for each condition are listed in the table (right). **P, $p < 0.01$. ***P, $p < 0.001$. See also Figure S5B.

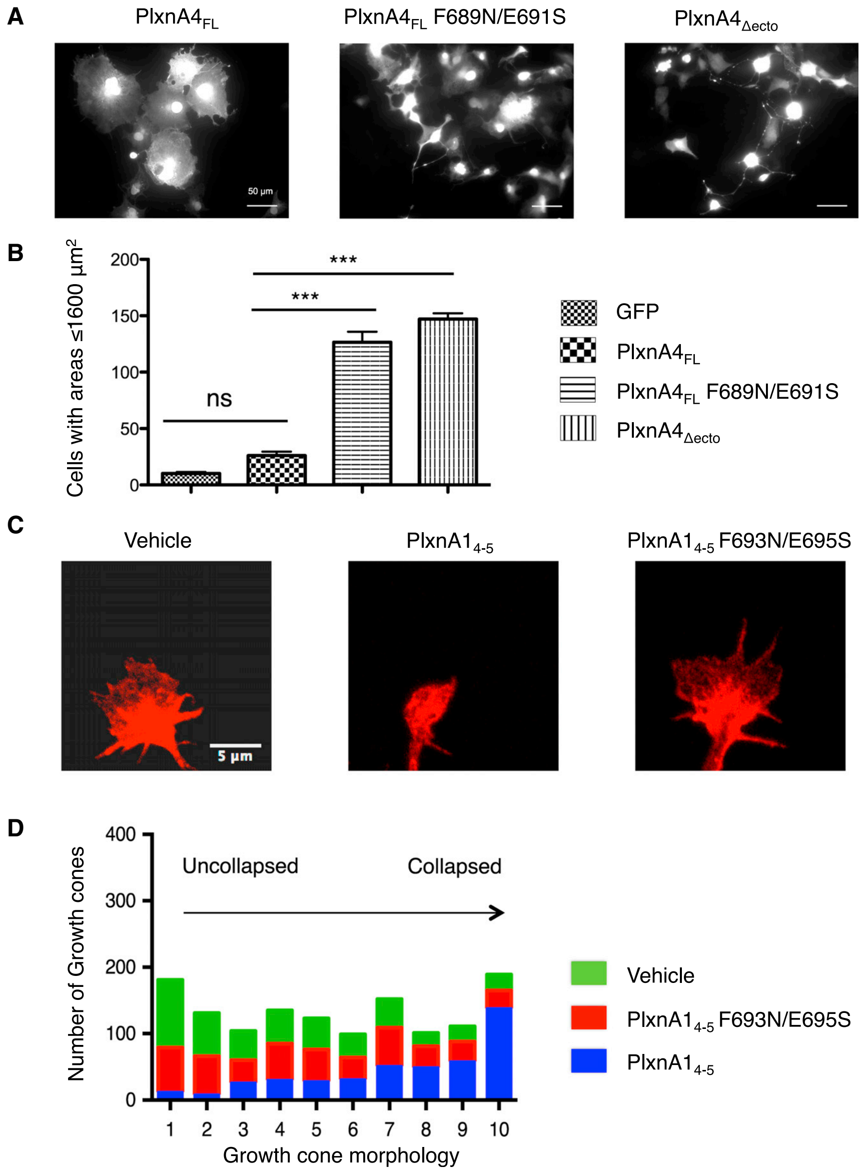

Figure 6. PlxnA *cis*-Interactions Mediate Functional Autoinhibition of PlxnA Signaling

(A) Representative images of COS-7 cells transfected with GFP and $PlxnA4_{FL}$, $PlxnA4_{FL}$ F689N/E691S, or $PlxnA4_{\Delta ecto}$ constructs. GFP signal is shown. Scale bar, 50 µm.

(B) The average number of GFP-positive COS-7 cells with an area less than 1,600 μm^2. Data are presented as means ± SEM from three to five independent experiments. N = 350–440 cells per condition. ***$p < 0.0001$, one-way ANOVA with Bonferroni multiple comparison test. ns, not significant.

(C) Representative images of dentate gyrus (DG) granule cell growth cones (stained for phalloidin in red) treated with vehicle, $PlxnA1_{4\text{-}5}$, or $PlxnA1_{4\text{-}5}$ F693N/E695S. Scale bar, 5 µm.

(D) Morphology of growth cones graded using a growth cone morphology matrix (Figure S6) that ranges from 1 (uncollapsed) to 10 (fully collapsed). Number of growth cones per condition is 453 (vehicle), 431 ($PlxnA1_{4\text{-}5}$,), and 442 ($PlxnA1_{4\text{-}5}$ F693N/E695S), respectively.

their growth cone receptors. Most guidance cue receptors are single-pass transmembrane (TM1) class cell-surface receptors, and studies in the past decades have shown that their signaling is often triggered, upon ligand binding, by receptor dimerization followed by clustering. Despite considerable progress in our understanding of the structure and function of axon guidance receptors, the multifaceted regulatory mechanisms that must underlie axon guidance receptor signaling remain poorly understood. These include the mechanisms that prevent premature signaling or that translate ligand binding into the activation of downstream signaling. To address these questions, we have focused on semaphorins and their PlxnA receptors, which have been implicated in the control of various aspects of neural circuit development (Pasterkamp, 2012). Thus far, our understanding of the molecular regulation of semaphorin–PlxnA signaling has been limited by a lack of information on the architecture and interaction modes of full-length PlxnA extracellular segments. The structural, microscopy, and functional data on full-length PlxnA ectodomains we report here show that PlxnA ectodomains are autoinhibited through intermolecular *cis*-interaction prior to ligand binding and support the notion of dimerization-based PlxnA activation upon ligand binding.

Data from multiple crystal structures of the PlxnA1, PlxnA2, and PlxnA4 ectodomain reveal that the extracellular segment in class A plexins adopts a distinctive, relatively rigid, ring-like conformation. Negative stain EM of $PlxnA1_{1\text{-}10}$ also shows this ring-like state to be the favored ectodomain conformation. The sequential arrangement of ten domains to form a well-defined, open ring is unexpected and striking. This distinctive ectodomain architecture immediately provides a satisfyingly simple solution to a longstanding puzzle. The precise arrangement of bivalent semaphorin interfacing with two diverging $PlxnA_{1\text{-}4}$ ectodomain fragments is conserved for Sema6A- and Sema3A-containing complexes (Janssen et al., 2010, 2012). How does a recognition assembly, involving only the N-terminal (sema) of ten "beads-on-a-string" domains, position the membrane proximal tenth domain (IPT6) to initiate dimerization, and consequent activation, in the cytoplasmic region? Superposition of the crystal structure for a full-length PlxnA extracellular segment onto our previously reported $Sema6A_{ecto}$–$PlxnA2_{1\text{-}4}$ complex (Janssen et al., 2010) brings the two IPT6 domains into close proximity (Figure 7). The distances between the two PlxnA ectodomain C-termini in these modeled complexes range from 10 Å to 59 Å for the five PlxnA

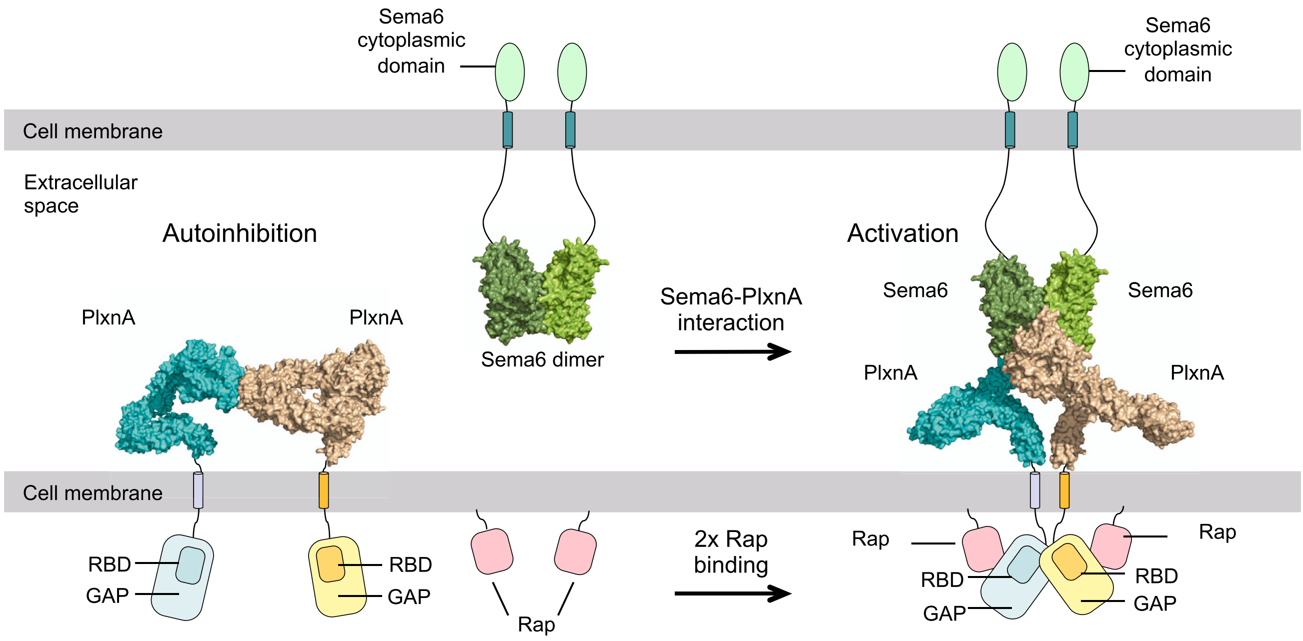

Figure 7. A Structural Model for PlxnA Autoinhibition Pre- and Activation Post-Ligand Binding

The PlxnA extracellular domain is represented by the 7.5 Å crystal structure of PlxnA4$_{1-10}$ (space group P4$_1$, see Figure S1) and superposed onto the Sema6A$_{ecto}$–PlxnA2$_{1-4}$ complex structure (PDB: 3OKY) (Janssen et. al., 2010). The transmembrane and cytoplasmic region of PlxnA as well as the extracellular C-termini, transmembrane domain, and cytoplasmic region of Sema6 are illustrated as cartoons.

crystal structures that have all ten domains resolved. This positioning of the membrane proximal domains from two PlxnAs appears appropriate to promote interaction between the transmembrane regions and, ultimately, dimerization of the cytoplasmic region. The available structural information for the Sema3-PlxnA-neuropilin complex and neuropilin a1-a2-b1-b2 ectodomain segment (Janssen et al., 2012) can also be accommodated in this model. Based on these observations, we believe that the distinctive ring-like conformation of the PlxnA ectodomain provides a mechanism for efficient and rather precisely defined coupling between extracellular and intercellular dimerization states. Interestingly, negative stain EM uncovers a less-frequent, twisted-open chair-like conformation for PlxnA1$_{1-10}$ generated by a 180° rotation around a midway hinge in the ring-like stalk. The fact that this conformation is the only alternative to the ring-like conformation suggests it may play a role in another aspect of PlxnA signaling, possibly the recently discovered PlxnAs–Sema6s *cis*-interaction that modulates PlxnA signaling in different neuronal populations (Andermatt et al., 2014; Haklai-Topper et al., 2010; Sun et al., 2013). However, uncovering what specific role (if any) the chair-like conformation plays will require significant further experimentation.

Our studies also uncover pre-signaling autoinhibition by intermolecular, head-to-stalk interactions in the receptor ectodomains as a novel regulatory mechanism for PlxnAs. Although the PlxnA head-to-stalk interaction appears relatively weak when measured in solution, for *cis*-interaction occurring between plexins tethered to the same plasma membrane, there is potential for substantial enhancement in binding affinity (Wu et al., 2011). We find that manipulations that disrupt intermolecular *cis*- interactions lead to constitutive plexin signaling and growth cone collapse. Previous work has hinted at the presence of auto-inhibitory intramolecular plexin interactions (Takahashi and Strittmatter, 2001). However, based on the structural data we report, now it seems unlikely that PlxnA ectodomains can engage in intramolecular interactions. Conversely, our work suggests that it is the combination of distinct structure and intermolecular, head-to-stalk interactions of the PlxnA ectodomains that maintains separation of the transmembrane and cytoplasmic segments pre-signaling (Figure 7). One should note that the mutated PlxnAs are not restricted to the auto-inhibited head-to-stalk dimer form; instead, they are free to rearrange and move around on the cell surface. The transmembrane and cytoplasmic regions of two mutated PlxnAs may, in many ways, transiently come into close vicinity, which would inadvertently lead to cytoplasmic domain dimerization and signaling. This conclusion is supported by previous findings (Takahashi and Strittmatter, 2001) that demonstrate that deleting the entire ectodomain or the sema domain of PlxnAs has the same cell-collapsing effect as using a glycan to block the head-to-stalk interface. Data from disparate signaling systems point to the importance of pre-association and ligand-induced conformational reorganization in the regulation of receptor activity (Atanasova and Whitty, 2012). This outside-together-inside-apart mode of PlxnA pre-clustering can prevent cytoplasmic dimerization while permitting localization of receptors poised for rapid response to ligand binding. Such receptor pre-clustering may be necessary for the exquisite spatial-temporal control of signaling required in axon guidance. Whether this previously unsuspected autoinhibitory mechanism, based on

intermolecular interactions of receptor ectodomains, is employed by other classes of plexins or axon guidance signaling systems requires further investigation.

EXPERIMENTAL PROCEDURES

Protein Production, Crystallization, and Data Collection

Mouse PlxnA1, PlxnA2, and PlxnA4 were produced using the pHLsec vector (Aricescu et al., 2006) and expressed in HEK293S (Reeves et al., 2002) cells for crystallization and in HEK293T cells for all other experiments. Samples were purified from dialysed medium by immobilized metal-affinity and size-exclusion chromatography. Crystallization experiments were set up by mixing 100 nl (or 200 nl for $PlxnA2_{1\text{-}10}$) of protein solution with 100 nl reservoir solution at 20.5°C or 4.0°C. Optimization experiments including lysine methylation of protein samples and crystal dehydration are detailed in the Supplemental Experimental Procedures. Crystallographic data was collected at 100 K, and diffraction data were integrated, scaled, and merged with MOSFLM (Leslie, 2006) and SCALA (Evans, 2011) or AIMLESS in CCP4 (1994).

Structure Solution and Refinement

The sema-PSI2 domains of the 4.0 Å crystal structure of $PlxnA1_{1\text{-}10}$ were first solved by molecular replacement in PHASER (McCoy et al., 2007) using the structure of $PlxnA2_{1\text{-}4}$ (Janssen et al., 2010) (PDB: 3OKT) (54% sequence identity with $PlxnA1_{1\text{-}4}$). To solve the structures of the remaining stalk region, homology models of PlxnA1 domains IPT2-IPT5 were placed by molecular replacement in PHASER, and the resulting structure was further optimized by manual rebuilding in COOT (Emsley and Cowtan, 2004) and refinement in REFMAC (Murshudov et al., 2011) using jelly-body restraints (Nicholls et al., 2012). The 1.36 Å structure of $PlxnA2_{4\text{-}5}$ and 2.2 Å structure of $PlxnA1_{7\text{-}10}$ were solved by molecular replacement in PHASER using the corresponding partially refined domains from the initial 4.0 Å structure of $PlxnA1_{1\text{-}10}$. These refined structures were then used to aid further refinement of the 4.0 Å structure of $PlxnA1_{1\text{-}10}$. Our final model is only missing the following residues: 265–271, 1,153–1,164, 1210–1,216, and 1,228–1,236. The solution and refinement of each individual PlxnA1, PlxnA2, PlxnA4, $PlxnA2_{4\text{-}5}$, and $PlxnA1_{7\text{-}10}$ structures are detailed in the Supplemental Experimental Procedures.

Negative Stain EM

For the preparation of carbon-coated grids and electron microscope set up, we followed the previously described protocols for negative stain EM (Booth et al., 2011). Samples were imaged at room temperature using an FEI Tecnai T12 electron microscope operating at an acceleration voltage of 120 kV and a dose of ~15 electrons per $Å^2$. Images were taken using a 4k × 4k FEI Eagle™ CCD camera at a magnification of 55,000× with under-focus values ranging from 1.0 to 1.5 μm. Images analysis was performed using a combination of EM processing programs including EMAN2 (Tang et al., 2007), IMAGIC (Van Heel and Keegstra, 1981), SPIDER and WEB (Frank et al., 1996), and WellMAP (Flanagan et al., 2010), as detailed in the Supplemental Experimental Procedures.

MALS

MALS experiments based on size-exclusion chromatography were performed with a system in which an analytical Superdex S200 10/30 column (GE Healthcare) was coupled to static light-scattering (DAWN HELEOS II, Wyatt Technology), differential refractive index (Optilab rEX, Wyatt Technology), and Agilent 1200 UV (Agilent Techologies) detectors. Data analysis was done using the ASTRA software package (Wyatt Technology).

AUC

AUC sedimentation velocity experiments were conducted using a Beckman Optima XL-I analytical ultracentrifuge and an An-60 Ti rotor (Beckman) in absorbance mode (280 nm incident light) at 40,000 rpm rotor speed. A total number of 100 scans were collected with one scan every 6 min. Scans 6–100 were analyzed with Sedfit (Schuck, 2000) and a c(s)-based sedimentation profile analysis. Expected sedimentation coefficients of the structural models were predicted using PROHYDROUS-SOMO (Brookes et al., 2010). For the ring-like and chair-like monomer, it was 5.6S and 5.5S, respectively; for the ring-like and chair-like dimers, it was 8.4S and 7.8S, respectively; for the ring-like and chair-like trimers, it was 10.6S and 9.56S, respectively; and for the ring-like and chair-like tetramers, it was 12.4S and 11.2S, respectively. The systematic peak broadening and peak shift to higher absolute sedimentation coefficients in wild-type and mutant experiments is a general observation at decreasing concentrations (Brown et al., 2001).

SPR

SPR equilibrium experiments were performed on a Biacore T200 instrument (GE Healthcare) at 25°C in SPR running buffer (10 mM HEPES [pH 7.5], 150 mM NaCl, and 0.005% (v/v) Tween 20) and regeneration buffer (2 M magnesium chloride). Wild-type and mutant $PlxnA2_{4\text{-}5}$ were enzymatically biotinylated with BirA via an engineered C-terminal tag and attached to streptavidin covalently coupled to the surface of the SPR chip. PlxnA2 was used for SPR experiments because the $PlxnA2_{1\text{-}2}$ and $PlxnA2_{4\text{-}5}$ segments were much better expressed than their PlxnA1 counterparts. Data analysis was done using Biacore™ T200 Software v2.0 with the nonlinear curve fitting of a 1:1 Langmuir binding model (Langmuir, 1918) to calculate the equilibrium dissociation constant (K_d) and the maximum analyte binding value (B_{max}) via equation $R_{bound} = (C_A \times B_{max})/(C_A + K_d)$, where "$R_{bound}$" is measured in response units (RU) and C_A is the concentration of analyte.

Förster Energy Transfer by FRET-FLIM

COS-7 cells expressing fluorescently tagged PlxnA2 were imaged using a SP8-SMD Leica microscope from Leica Microsystems (Manheim, Germany) together with a 63×/1.4 oil immersion objective, a 440 nm pulsed laser at 40 MHz coupled with single photon counting electronics (PicoHarp 300) to excite mTFP1, and a Leica Hybrid external detector to record photons. To achieve high signal to noise and minimize artifacts, only cells with 700–1,000 photons per pixel and negligible amount of bleaching after a 2 × 2 image binning were included in the analysis (Leray et al., 2013; Padilla-Parra et al., 2008). The mean fluorescence lifetime ($\langle\tau\rangle$) and fraction of interacting donor (f_D) were calculated as described in in the Supplemental Experimental Procedures (Padilla-Parra et al., 2008, 2015; Zhao et al., 2014).

COS7 Collapse Assay

COS7 cells were cultured in DMEM (high glucose) supplemented with 10% FCS, penicillin-streptomycin, and L-glutamine. Cells were plated on glass coverslips at a density of 0.5–1 × 10^4 cell/cm^2 in a 12-well plate. At day in vitro (DIV) 1, cells were transfected with 0.5 μg of PlxnA4 constructs in pcDNA3.1 (+) plasmids together with eGFP using X-tremeGENE™ 9 (Roche) following the manufacturer's instruction. One day after transfection, cells were fixed in 4% PFA and immunostained for GFP. Images were acquired with a 40× objective with AxioScopeA1 (Zeiss) microscope. Cell area was measured using ImageJ as described previously (Takahashi and Strittmatter, 2001).

DG Growth Cone Assay

DG granule cell cultures were prepared as described (Van Battum et al., 2014; van Erp et al., 2015). In brief, dissociated DG granule cells were prepared from P6–8 mouse hippocampi and cultured in Neurobasal medium supplemented with B-27, L-glutamine, penicillin-streptomycin, and β-mercaptoethanol on coverslips coated with poly-D-lysine (20 mg/ml) and laminin (40 μg/ml) in a humidified incubator at 37°C and 5% CO_2. On DIV1, cells were treated with vehicle or wild-type or mutant $PlxnA1_{4\text{-}5}$ recombinant proteins (3 mg/ml) for 30 min at 37°C, fixed in 4% PFA and sucrose for 20 min at room temperature, and immunostained with anti-βIII-tubulin antibodies and phalloidin. Images were acquired using a 100× objective on an AxioScopeA1 (Zeiss) microscope and analyzed using a growth cone morphology matrix (Figure S6) in an observer-blind manner.

ACCESSION NUMBERS

The structure factors and coordinates for $PlxnA1_{1\text{-}10}$ (spacegroup $P4_32_12$; 4 Å, $P2_1$ and $P4_32_12$; 6 Å), $PlxnA1_{7\text{-}10}$, $PlxnA2_{1\text{-}10}$, $PlxnA2_{4\text{-}5}$, and $PlxnA4_{1\text{-}10}$

(spacegroup P4$_1$, P2$_1$, P4$_3$2$_1$2, and P4$_3$22) have been deposited in the Protein Data Bank with accession codes PDB: 5L56, 5L59, 5L5C, 5L7N, 5L5G, 5L74, 5L5K, 5L5L, 5L5M, 5L5N.

SUPPLEMENTAL INFORMATION

Supplemental Information includes six figures, one table, and Supplemental Experimental Procedures and can be found with this article online at http://dx.doi.org/10.1016/j.neuron.2016.06.018.

AUTHOR CONTRIBUTIONS

Y.K., B.J.C.J., T.M., R.J.P., and E.Y.J. designed the experiments. Y.K., B.J.C.J., and T.M. performed structural and biophysical measurements. Y.K. carried out negative stain EM and light microscopy experiments. R.K. helped to perform localization experiments, C.H.C. was involved in negative stain EM, T.N. was involved in AUC experiments, R.J.C.G. helped interpret EM and AUC data, and S.P.-P. guided and interpreted the FRET-FLIM experiments. V.R.V. conducted COS-7 cell and DG growth cone assays. T.M. and V.R.V. contributed equally to this work. Main authors responsible for writing and editing the paper are Y.K., B.J.C.J., T.M., R.G., S.P.-P., R.J.P., and E.Y.J.

ACKNOWLEDGMENTS

We thank the staff of Diamond beamlines I02, I03, I04, I04-1, and I24; and the staff of ESRF beamline ID23-2 for assistance with diffraction data collection; The Cellular Imaging Core from the Wellcome Trust Centre for Human Genetics; Weixian Lu and Yuguang Zhao for mammalian cell tissue culture; Thomas S. Walter for help with crystallization; and Santiago Manrique Zuñiga for help with negative stain EM. The Biophysical Facility at the Department of Biochemistry, University of Oxford managed by David Staunton is gratefully acknowledged for experiments performed there. This work was funded by Cancer Research UK, the UK Medical Research Council (to E.Y.J., A10976 and G9900061), and Wellcome Trust (grant 090532/Z/09/Z supporting the Wellcome Trust Centre for Human Genetics). R.J.P. was supported by a VICI grant from the Netherlands Organization for Scientific Research (NWO) and a grant from the Epilepsiefonds (12-08). Y.K. was supported by the Clarendon Fund in conjunction with the Nuffield Department of Clinical Medicine; B.J.C.J. was funded by a Long-Term Fellowship from the Human Frontier Science Program and a VIDI grant from NWO; T.M. was supported by the UK Medical Research Council and is a recipient of a Long-Term Fellowship from the Human Frontier Science Program. S.P.-P.is supported by the Nuffield Department of Medicine Leadership Fellowship and the Wellcome Trust.

Received: October 24, 2014
Revised: May 11, 2016
Accepted: June 7, 2016
Published: July 7, 2016

REFERENCES

Andermatt, I., Wilson, N.H., Bergmann, T., Mauti, O., Gesemann, M., Sockanathan, S., and Stoeckli, E.T. (2014). Semaphorin 6B acts as a receptor in post-crossing commissural axon guidance. Development *141*, 3709–3720.

Antipenko, A., Himanen, J.-P., van Leyen, K., Nardi-Dei, V., Lesniak, J., Barton, W.A., Rajashankar, K.R., Lu, M., Hoemme, C., Püschel, A.W., and Nikolov, D.B. (2003). Structure of the semaphorin-3A receptor binding module. Neuron *39*, 589–598.

Aricescu, A.R., Lu, W., and Jones, E.Y. (2006). A time- and cost-efficient system for high-level protein production in mammalian cells. Acta Crystallogr. D Biol. Crystallogr. *62*, 1243–1250.

Atanasova, M., and Whitty, A. (2012). Understanding cytokine and growth factor receptor activation mechanisms. Crit. Rev. Biochem. Mol. Biol. *47*, 502–530.

Bashaw, G.J., and Klein, R. (2010). Signaling from axon guidance receptors. Cold Spring Harb. Perspect. Biol. *2*, a001941.

Booth, D.S., Avila-Sakar, A., and Cheng, Y. (2011). Visualizing proteins and macromolecular complexes by negative stain em: from grid preparation to image acquisition. J. Vis. Exp. http://dx.doi.org/10.3791/3227.

Brookes, E., Cao, W., and Demeler, B. (2010). A two-dimensional spectrum analysis for sedimentation velocity experiments of mixtures with heterogeneity in molecular weight and shape. Eur. Biophys. J. *39*, 405–414.

Brown, P.H., Balbo, A., and Schuck, P. (2001). Characterizing Protein-Protein Interactions by Sedimentation Velocity Analytical Ultracentrifugation (Hoboken, NJ, USA: John Wiley & Sons, Inc.).

Emsley, P., and Cowtan, K. (2004). Coot: model-building tools for molecular graphics. Acta Crystallogr. D Biol. Crystallogr. *60*, 2126–2132.

Evans, P.R. (2011). An introduction to data reduction: space-group determination, scaling and intensity statistics. Acta Crystallogr. D Biol. Crystallogr. *67*, 282–292.

Flanagan, J.F., 4th, Namy, O., Brierley, I., and Gilbert, R.J.C. (2010). Direct observation of distinct A/P hybrid-state tRNAs in translocating ribosomes. Structure *18*, 257–264.

Frank, J., Radermacher, M., Penczek, P., Zhu, J., Li, Y., Ladjadj, M., and Leith, A. (1996). SPIDER and WEB: processing and visualization of images in 3D electron microscopy and related fields. J. Struct. Biol. *116*, 190–199.

Haklai-Topper, L., Mlechkovich, G., Savariego, D., Gokhman, I., and Yaron, A. (2010). Cis interaction between Semaphorin6A and Plexin-A4 modulates the repulsive response to Sema6A. EMBO J. *29*, 2635–2645.

Janssen, B.J.C., Robinson, R.A., Pérez-Brangulí, F., Bell, C.H., Mitchell, K.J., Siebold, C., and Jones, E.Y. (2010). Structural basis of semaphorin-plexin signalling. Nature *467*, 1118–1122.

Janssen, B.J.C., Malinauskas, T., Weir, G.A., Cader, M.Z., Siebold, C., and Jones, E.Y. (2012). Neuropilins lock secreted semaphorins onto plexins in a ternary signaling complex. Nat. Struct. Mol. Biol. *19*, 1293–1299.

Jongbloets, B.C., and Pasterkamp, R.J. (2014). Semaphorin signalling during development. Development *141*, 3292–3297.

Kolodkin, A.L., and Tessier-Lavigne, M. (2011). Mechanisms and molecules of neuronal wiring: a primer. Cold Spring Harb. Perspect. Biol. *3*, a001727.

Langmuir, I. (1918). THE ADSORPTION OF GASES ON PLANE SURFACES OF GLASS, MICA AND PLATINUM. J. Am. Chem. Soc. *40*, 1361–1403.

Leray, A., Padilla-Parra, S., Roul, J., Héliot, L., and Tramier, M. (2013). Spatio-Temporal Quantification of FRET in living cells by fast time-domain FLIM: a comparative study of non-fitting methods [corrected]. PLoS ONE *8*, e69335.

Leslie, A.G.W. (2006). The integration of macromolecular diffraction data. Acta Crystallogr. D Biol. Crystallogr. *62*, 48–57.

Liu, H., Juo, Z.S., Shim, A.H.-R., Focia, P.J., Chen, X., Garcia, K.C., and He, X. (2010). Structural basis of semaphorin-plexin recognition and viral mimicry from Sema7A and A39R complexes with PlexinC1. Cell *142*, 749–761.

Marita, M., Wang, Y., Kaliszewski, M.J., Skinner, K.C., Comar, W.D., Shi, X., Dasari, P., Zhang, X., and Smith, A.W. (2015). Class A Plexins Are Organized as Preformed Inactive Dimers on the Cell Surface. Biophys. J. *109*, 1937–1945.

McCoy, A.J., Grosse-Kunstleve, R.W., Adams, P.D., Winn, M.D., Storoni, L.C., and Read, R.J. (2007). Phaser crystallographic software. J. Appl. Cryst. *40*, 658–674.

Murshudov, G.N., Skubák, P., Lebedev, A.A., Pannu, N.S., Steiner, R.A., Nicholls, R.A., Winn, M.D., Long, F., and Vagin, A.A. (2011). REFMAC5 for the refinement of macromolecular crystal structures. Acta Crystallogr. D Biol. Crystallogr. *67*, 355–367.

Nicholls, R.A., Long, F., and Murshudov, G.N. (2012). Low-resolution refinement tools in REFMAC5. Acta Crystallogr. D Biol. Crystallogr. *68*, 404–417.

Nogi, T., Yasui, N., Mihara, E., Matsunaga, Y., Noda, M., Yamashita, N., Toyofuku, T., Uchiyama, S., Goshima, Y., Kumanogoh, A., and Takagi, J. (2010). Structural basis for semaphorin signalling through the plexin receptor. Nature *467*, 1123–1127.

Padilla-Parra, S., Audugé, N., Coppey-Moisan, M., and Tramier, M. (2008). Quantitative FRET analysis by fast acquisition time domain FLIM at high spatial resolution in living cells. Biophys. J. *95*, 2976–2988.

Padilla-Parra, S., Audugé, N., Tramier, M., and Coppey-Moisan, M. (2015). Time-domain fluorescence lifetime imaging microscopy: a quantitative method to follow transient protein-protein interactions in living cells. Cold Spring Harb. Protoc. *2015*, 508–521.

Pasterkamp, R.J. (2012). Getting neural circuits into shape with semaphorins. Nat. Rev. Neurosci. *13*, 605–618.

Reeves, P.J., Callewaert, N., Contreras, R., and Khorana, H.G. (2002). Structure and function in rhodopsin: high-level expression of rhodopsin with restricted and homogeneous N-glycosylation by a tetracycline-inducible N-acetylglucosaminyltransferase I-negative HEK293S stable mammalian cell line. Proc. Natl. Acad. Sci. USA *99*, 13419–13424.

Schuck, P. (2000). Size-distribution analysis of macromolecules by sedimentation velocity ultracentrifugation and lamm equation modeling. Biophys. J. *78*, 1606–1619.

Siebold, C., and Jones, E.Y. (2013). Structural insights into semaphorins and their receptors. Semin. Cell Dev. Biol. *24*, 139–145.

Sun, L.O., Jiang, Z., Rivlin-Etzion, M., Hand, R., Brady, C.M., Matsuoka, R.L., Yau, K.-W., Feller, M.B., and Kolodkin, A.L. (2013). On and off retinal circuit assembly by divergent molecular mechanisms. Science *342*, 1241974–1241974.

Suto, F., Murakami, Y., Nakamura, F., Goshima, Y., and Fujisawa, H. (2003). Identification and characterization of a novel mouse plexin, plexin-A4. Mech. Dev. *120*, 385–396.

Suto, F., Tsuboi, M., Kamiya, H., Mizuno, H., Kiyama, Y., Komai, S., Shimizu, M., Sanbo, M., Yagi, T., Hiromi, Y., et al. (2007). Interactions between plexin-A2, plexin-A4, and semaphorin 6A control lamina-restricted projection of hippocampal mossy fibers. Neuron *53*, 535–547.

Takahashi, T., and Strittmatter, S.M. (2001). Plexina1 autoinhibition by the plexin sema domain. Neuron *29*, 429–439.

Tang, G., Peng, L., Baldwin, P.R., Mann, D.S., Jiang, W., Rees, I., and Ludtke, S.J. (2007). EMAN2: an extensible image processing suite for electron microscopy. J. Struct. Biol. *157*, 38–46.

Van Battum, E.Y., Gunput, R.-A.F., Lemstra, S., Groen, E.J.N., Yu, K.L., Adolfs, Y., Zhou, Y., Hoogenraad, C.C., Yoshida, Y., Schachner, M., et al. (2014). The intracellular redox protein MICAL-1 regulates the development of hippocampal mossy fibre connections. Nat. Commun. *5*, 4317.

van Erp, S., van den Heuvel, D.M.A., Fujita, Y., Robinson, R.A., Hellemons, A.J.C.G.M., Adolfs, Y., Van Battum, E.Y., Blokhuis, A.M., Kuijpers, M., Demmers, J.A.A., et al. (2015). Lrig2 Negatively Regulates Ectodomain Shedding of Axon Guidance Receptors by ADAM Proteases. Dev. Cell *35*, 537–552.

Van Heel, M., and Keegstra, W. (1981). IMAGIC: A fast, flexible and friendly image analysis software system. Ultramicroscopy *7*, 113–129.

Wang, Y., He, H., Srivastava, N., Vikarunnessa, S., Chen, Y.B., Jiang, J., Cowan, C.W., and Zhang, X. (2012). Plexins are GTPase-activating proteins for Rap and are activated by induced dimerization. Sci. Signal. *5*, ra6–ra6.

Wang, Y., Pascoe, H.G., Brautigam, C.A., He, H., and Zhang, X. (2013). Structural basis for activation and non-canonical catalysis of the Rap GTPase activating protein domain of plexin. eLife *2*, e01279–e01279.

Wu, Y., Vendome, J., Shapiro, L., Ben-Shaul, A., and Honig, B. (2011). Transforming binding affinities from three dimensions to two with application to cadherin clustering. Nature *475*, 510–513.

Yamada, H., Padilla-Parra, S., Park, S.-J., Itoh, T., Chaineau, M., Monaldi, I., Cremona, O., Benfenati, F., De Camilli, P., Coppey-Moisan, M., et al. (2009). Dynamic interaction of amphiphysin with N-WASP regulates actin assembly. J. Biol. Chem. *284*, 34244–34256.

Yogev, S., and Shen, K. (2014). Cellular and molecular mechanisms of synaptic specificity. Annu. Rev. Cell Dev. Biol. *30*, 417–437.

Zhao, Y., Ren, J., Padilla-Parra, S., Fry, E.E., and Stuart, D.I. (2014). Lysosome sorting of β-glucocerebrosidase by LIMP-2 is targeted by the mannose 6-phosphate receptor. Nat. Commun. *5*, 4321.

Neuron, Volume *91*

Supplemental Information

Structural Basis for Plexin Activation and Regulation

Youxin Kong, Bert J.C. Janssen, Tomas Malinauskas, Vamshidhar R. Vangoor, Charlotte H. Coles, Rainer Kaufmann, Tao Ni, Robert J.C. Gilbert, Sergi Padilla-Parra, R. Jeroen Pasterkamp, and E. Yvonne Jones

Structural Basis for Plexin Activation and Regulation

Youxin Kong[1*], Bert J.C. Janssen[1, 4*], Tomas Malinauskas[1], Vamshidhar R. Vangoor[2], Charlotte H. Coles[1, 6], Rainer Kaufmann[1, 3], Tao Ni[1], Robert J.C. Gilbert[1], Sergi Padilla-Parra[1], R. Jeroen Pasterkamp[2#] & E. Yvonne Jones[1#]

[1] Division of Structural Biology, Wellcome Trust Centre for Human Genetics, University of Oxford, Oxford, United Kingdom

[2] Department of Translational Neuroscience, Brain Center Rudolf Magnus, University Medical Center Utrecht, Utrecht, The Netherlands

[3] Department of Biochemistry, University of Oxford, Oxford, United Kingdom

[4] Present address: Crystal and Structural Chemistry, Bijvoet Center for Biomolecular Research, Department of Chemistry, Faculty of Science, Utrecht University, Utrecht, The Netherlands

[6] Present address: Laboratory for Axon Growth and Regeneration, German Center for Neurodegenerative Diseases (DZNE), Bonn, Germany.

[*] These authors contributed equally to this work

[#] Correspondence and requests for materials should be addressed to R.J.P. or E.Y.J. (Email: r.j.pasterkamp@umcutrecht.nl or yvonne@strubi.ox.ac.uk).

SUPPLEMENTAL INFORMATION

This file includes:

Supplemental Figures S1 to S6

Supplemental Figure Legends

Supplemental Table S1

Extended Experimental Procedures

Supplemental References

Figure S1

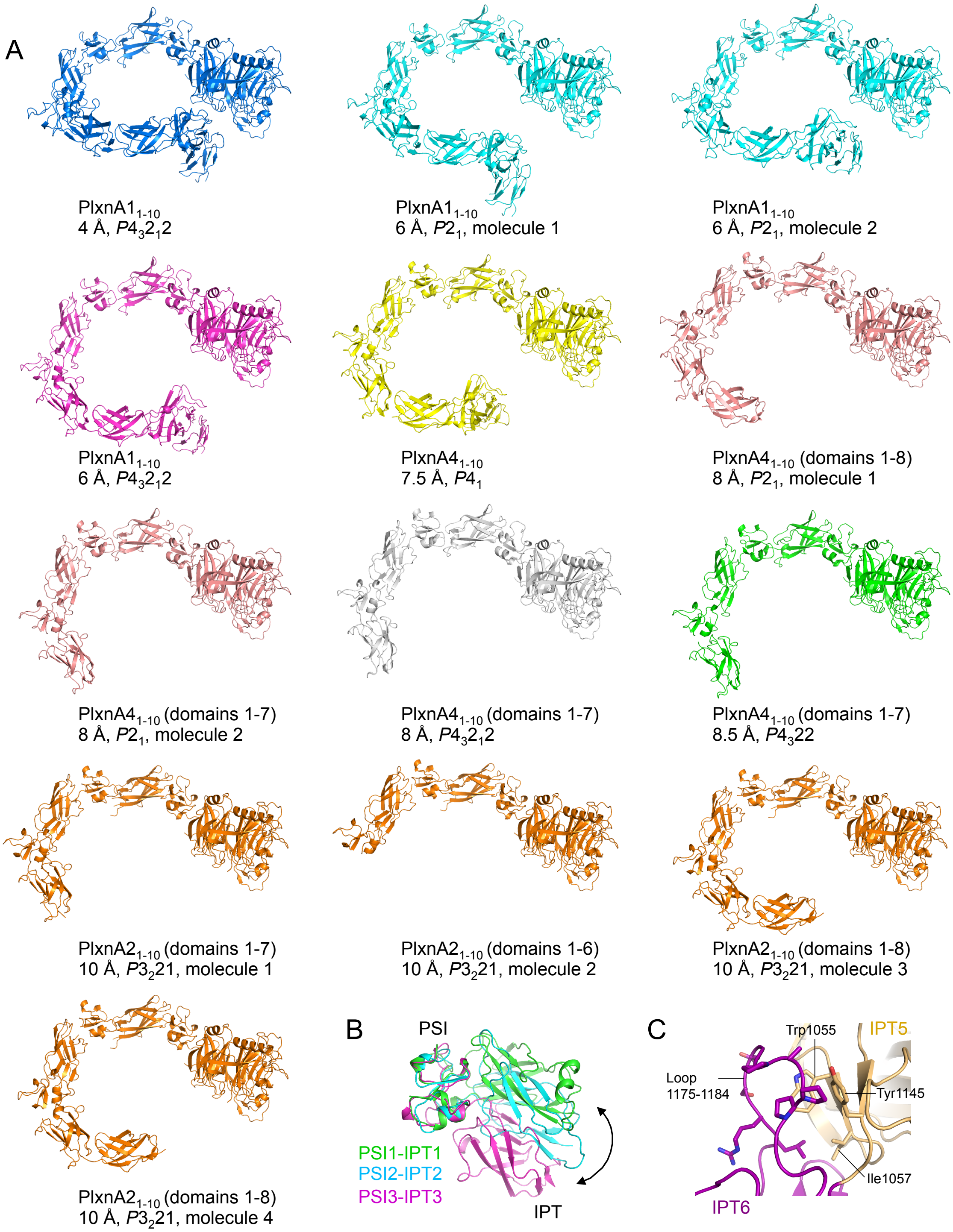

A
PlxnA1$_{1-10}$
4 Å, $P4_32_12$
PlxnA1$_{1-10}$
6 Å, $P2_1$, molecule 1
PlxnA1$_{1-10}$
6 Å, $P2_1$, molecule 2
PlxnA1$_{1-10}$
6 Å, $P4_32_12$
PlxnA4$_{1-10}$
7.5 Å, $P4_1$
PlxnA4$_{1-10}$ (domains 1-8)
8 Å, $P2_1$, molecule 1
PlxnA4$_{1-10}$ (domains 1-7)
8 Å, $P2_1$, molecule 2
PlxnA4$_{1-10}$ (domains 1-7)
8 Å, $P4_32_12$
PlxnA4$_{1-10}$ (domains 1-7)
8.5 Å, $P4_322$
PlxnA2$_{1-10}$ (domains 1-7)
10 Å, $P3_221$, molecule 1
PlxnA2$_{1-10}$ (domains 1-6)
10 Å, $P3_221$, molecule 2
PlxnA2$_{1-10}$ (domains 1-8)
10 Å, $P3_221$, molecule 3
PlxnA2$_{1-10}$ (domains 1-8)
10 Å, $P3_221$, molecule 4
B
PSI
PSI1-IPT1
PSI2-IPT2
PSI3-IPT3
IPT
C
Trp1055
IPT5
Loop
1175-1184
Tyr1145
Ile1057
IPT6

Figure S2

A

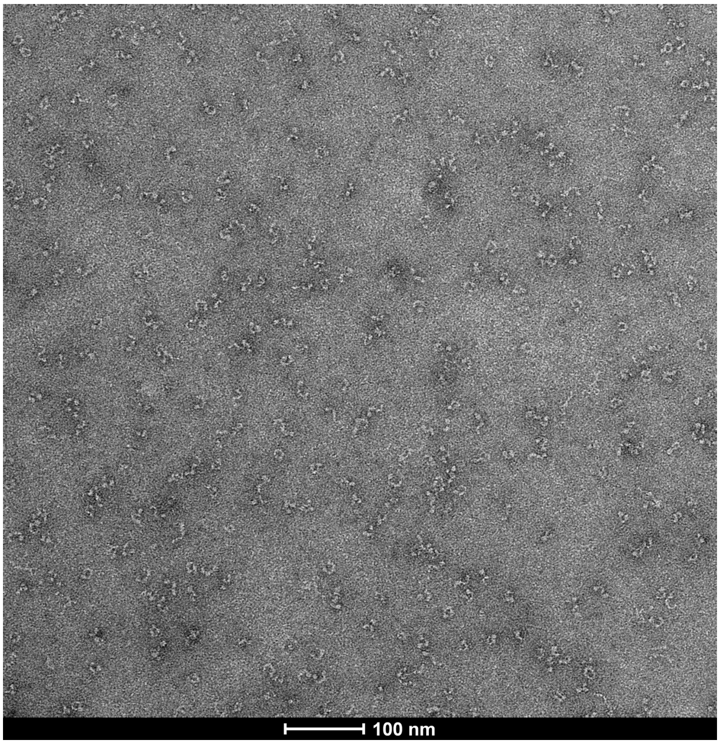

B

I.

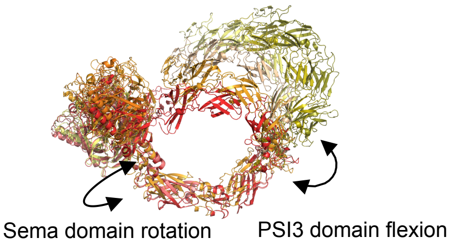

II.

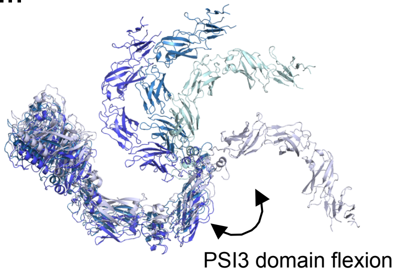

C

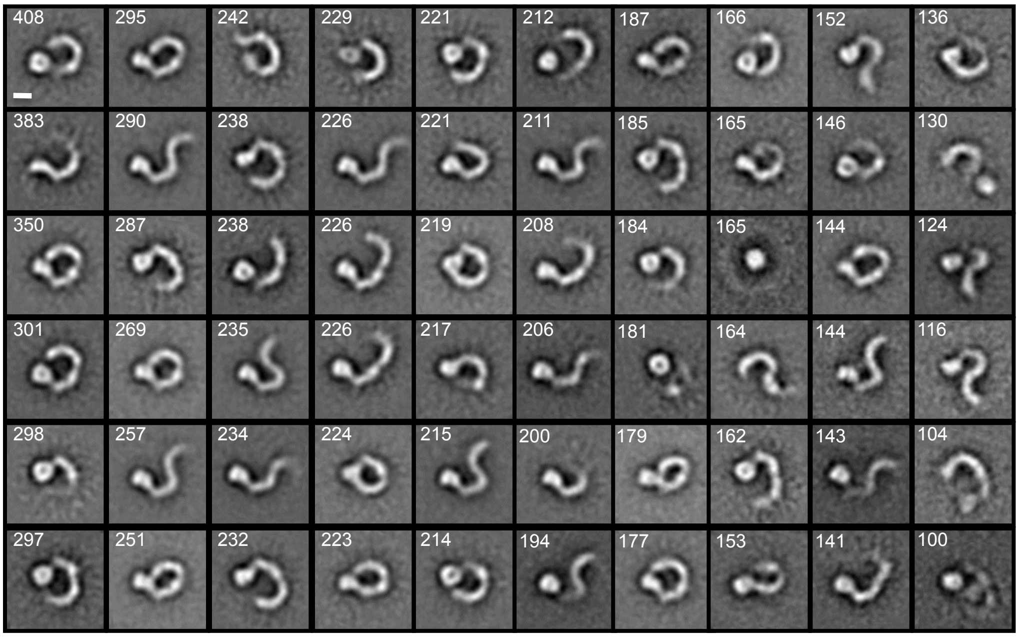

Figure S3

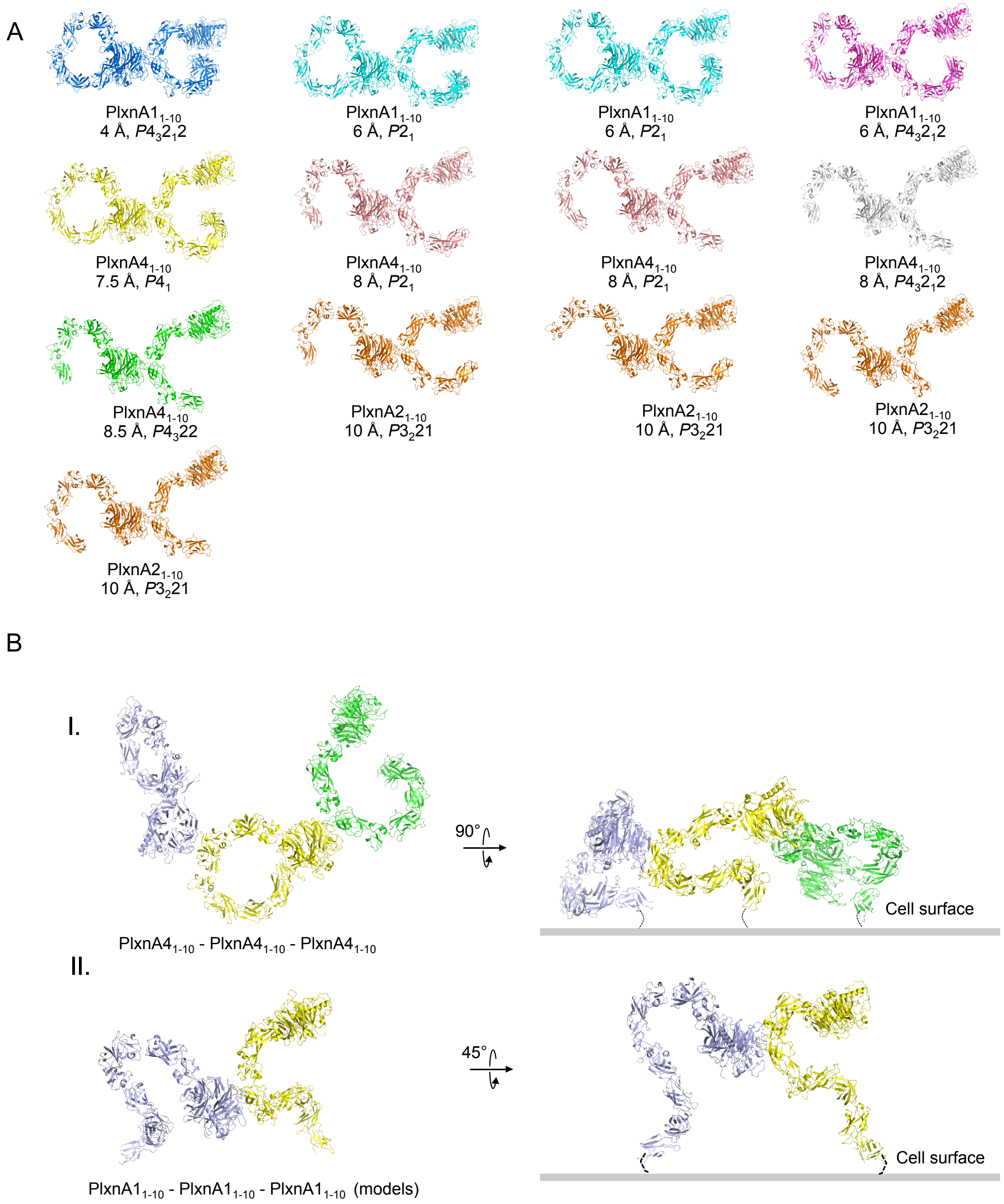
A
PlxnA1$_{1-10}$
4 Å, $P4_32_12$
PlxnA1$_{1-10}$
6 Å, $P2_1$
PlxnA1$_{1-10}$
6 Å, $P2_1$
PlxnA1$_{1-10}$
6 Å, $P4_32_12$
PlxnA4$_{1-10}$
7.5 Å, $P4_1$
PlxnA4$_{1-10}$
8 Å, $P2_1$
PlxnA4$_{1-10}$
8 Å, $P2_1$
PlxnA4$_{1-10}$
8 Å, $P4_32_12$
PlxnA4$_{1-10}$
8.5 Å, $P4_322$
PlxnA2$_{1-10}$
10 Å, $P3_221$
PlxnA2$_{1-10}$
10 Å, $P3_221$
PlxnA2$_{1-10}$
10 Å, $P3_221$
PlxnA2$_{1-10}$
10 Å, $P3_221$
B
I.
90°
Cell surface
PlxnA4$_{1-10}$ - PlxnA4$_{1-10}$ - PlxnA4$_{1-10}$
II.
45°
Cell surface
PlxnA1$_{1-10}$ - PlxnA1$_{1-10}$ - PlxnA1$_{1-10}$ (models)

Figure S4

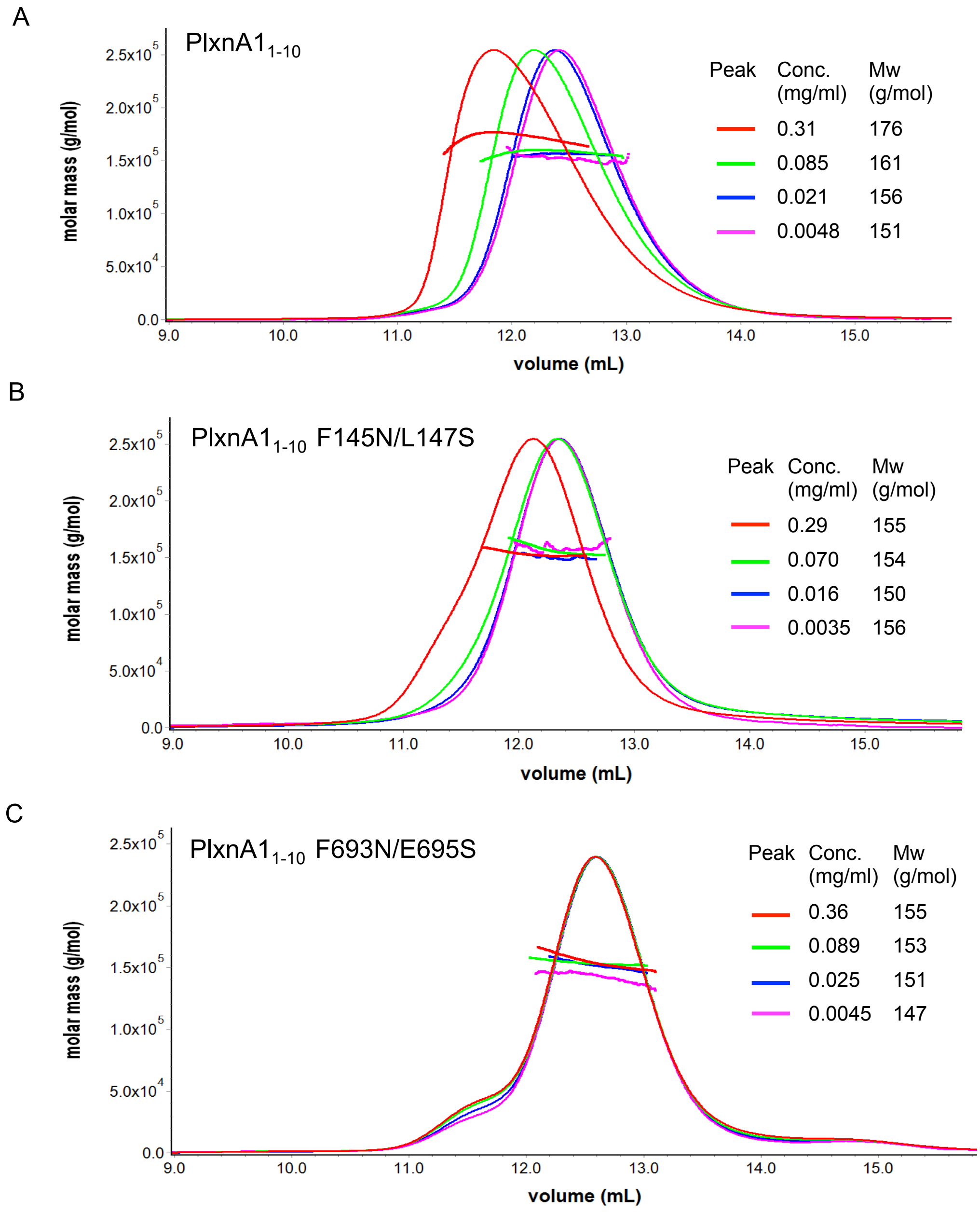

A
PlxnA1 1-10
molar mass (g/mol)
volume (mL)
Peak
Conc. (mg/ml)
Mw (g/mol)
0.31 176
0.085 161
0.021 156
0.0048 151
B
PlxnA1 1-10 F145N/L147S
molar mass (g/mol)
volume (mL)
Peak
Conc. (mg/ml)
Mw (g/mol)
0.29 155
0.070 154
0.016 150
0.0035 156
C
PlxnA1 1-10 F693N/E695S
molar mass (g/mol)
volume (mL)
Peak
Conc. (mg/ml)
Mw (g/mol)
0.36 155
0.089 153
0.025 151
0.0045 147

Figure S5

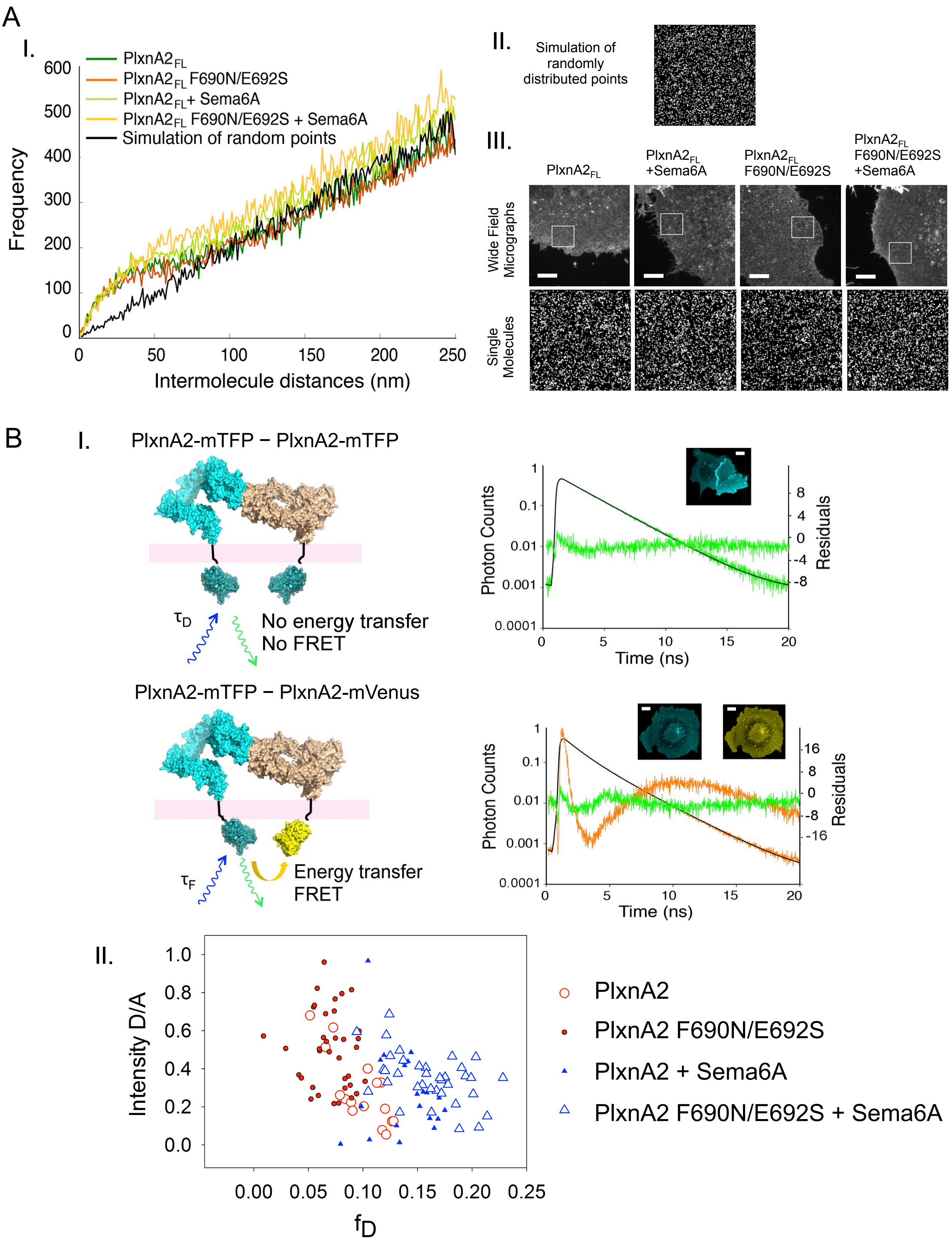

A
I.
PlxnA2FL
PlxnA2FL F690N/E692S
PlxnA2FL+ Sema6A
PlxnA2FL F690N/E692S + Sema6A
Simulation of random points
Frequency
Intermolecule distances (nm)
II.
Simulation of randomly distributed points
III.
PlxnA2FL
PlxnA2FL +Sema6A
PlxnA2FL F690N/E692S
PlxnA2FL F690N/E692S +Sema6A
Wide Field Micrographs
Single Molecules
B
I.
PlxnA2-mTFP − PlxnA2-mTFP
τD
No energy transfer
No FRET
Photon Counts
Residuals
Time (ns)
PlxnA2-mTFP − PlxnA2-mVenus
τF
Energy transfer
FRET
II.
Intensity D/A
fD
PlxnA2
PlxnA2 F690N/E692S
PlxnA2 + Sema6A
PlxnA2 F690N/E692S + Sema6A

Figure S6

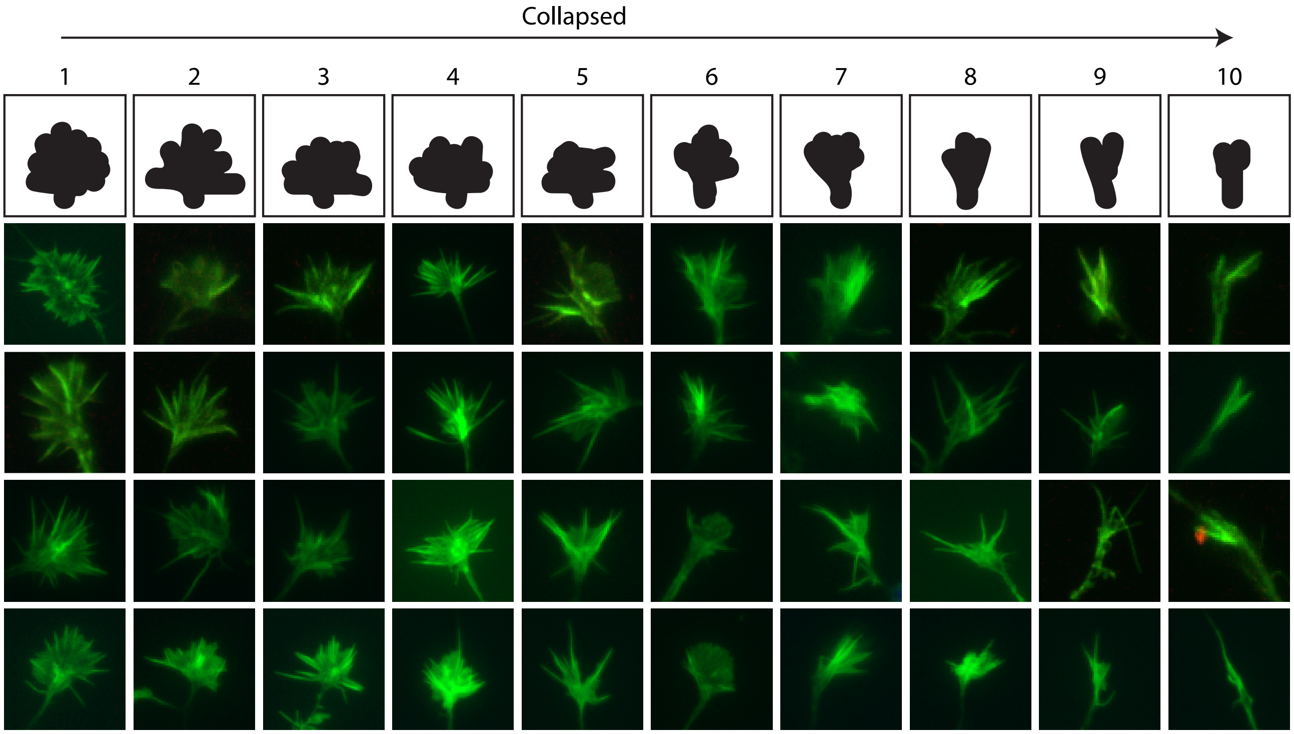

SUPPLEMENTAL FIGURE LEGENDS

Figure S1, related to Figure 1: Crystal structures of the extracellular segments of PlnxA1, PlxnA2 and PlxnA4. (A) Ribbon representation of all full extracellular segment crystal structures of PlxnA1, PlxnA2 and PlxnA4. The domains included in each structure are shown in parenthesis. Highest resolution of each structure and the space group are indicated (see also Table S1). (B) Ribbon representation of a PSI domain based superposition of the three PSI-IPT segments from the 4 Å $PlxnA1_{1\text{-}10}$ structure. The PSI3-IPT3 segment has a different interface and a different interdomain angle compared to the PSI1-IPT1 and PSI2-IPT2 segments. (C) Detailed view of the loop 1175-1184 and residues Trp1055, Ile1057 and Tyr1145 mediated IPT5-IPT6 interaction in $PlxnA1_{7\text{-}10}$.

Figure S2, related to Figure 2 and Extended Experimental Procedures: Negative stain EM and structural modelling of $PlxnA1_{1\text{-}10}$. (A) Representative negative stain electron micrograph of $PlxnA1_{1\text{-}10}$ particles. Scale bar, 100 nm. (B) Modelling of the $PlxnA1_{1\text{-}10}$ ring-like (I) and chair-like (II) conformations. For the ring-like conformations (I), the $PlxnA1_{1\text{-}10}$ stalk region was anchored at the IPT2–PSI3 (domains 5-6) interface and PSI3-IPT6 (domains 6-10) was flexed as a rigid body closer to or away from the N-terminal half of the stalk region. For the alternative, chair-like conformations (II), domains PSI3-IPT6 (domains 6-10) were first rotated by 180° around the IPT2-PSI3 (domains 5-6) linker region (805Y-806K) and then flexed to generate the series of chair-like conformations (II). One should note that the disc-like projection of the sema domain observed for some ring-like class averages required a rotation of the sema domain around the sema-PSI1 linker (Figure S2B I) although this orientation has not been observed in any of the crystal structures. (C) Class averages of $PlxnA1_{1\text{-}10}$ particles ranked from top left to bottom right based on

the number of particles within each class (listed on the top left corner). The size of each image is 298 Å by 298 Å. Scale bar, 5 nm.

Figure S3, related to Figure 3: Cell-surface attached PlxnA dimers and trimers formed via the sema–PSI2-IPT2 interface. (A) Ribbon representation of the ring-like PlxnA–PlxnA dimers formed via the sema–PSI2-IPT2 interface present in all thirteen PlxnA full ectodomain crystal structures. Highest resolution of the diffraction data and the space group are indicated (same coloring scheme as in Figure S1). (B) Possible cell surface orientations of PlxnA trimers based on the ring-like conformation (I) or dimers based on the chair-like conformation (II). The structures in (I) are based on the conformation and packing of the PlxnA4$_{1\text{-}10}$ crystal structure (space group $P4_1$). The chair-like structures in (II) are generated from a model which best correlates with the most populated negative stain EM chair-like class average (see Figure 2 and Figure S2) while keeping the same interface as in (I). Formation of a PlxnA trimer based on the chair-like conformation or a mixture of ring-like and chair-like conformations appeared sterically incompatible with cell surface attachment (data not shown).

Figure S4, related to Figure 4: MALS experiments reveal intermolecular PlxnA1$_{1\text{-}10}$ interaction via the sema–PSI2-IPT2 interface in solution. (A) MALS size exclusion chromatography elution profile of PlxnA1$_{1\text{-}10}$ shows a shift of elution maximum towards larger retention volumes at decreasing injected concentrations, indicating protein–protein interactions. The mass of 151 kDa determined at the lowest concentration fits well with a calculated mass of 156 kDa for a PlxnA1$_{1\text{-}10}$ monomer. (B) PlxnA1$_{1\text{-}10}$ F145N/L147S (an N-linked glycan introduced on the sema domain) has a less pronounced peak shift and the weight average mass indicates a

predominantly monomeric species. Despite a peak shift to lower retention volumes at the highest concentration (4 mg/ml), the measured molar mass of $PlxnA1_{1-10}$ F145N/L147S at all concentrations (150-156 kDa) remains very similar to the $PlxnA1_{1-10}$ monomer. This may be because when high concentrations of $PlxnA1_{1-10}$ F145N/L147S travelled through the SEC column, its initial weak interactions gradually diminished during dilutions in the run. (C) $PlxnA1_{1-10}$ F693N/E695S (an N-linked glycan introduced on the PSI2 domain) is monomeric at all concentrations; there is no peak shift and the weight average mass indicates the presence of only monomers. The red, blue, green and magenta traces correspond to 28.1 μM (4 mg/ml), 7.0 μM (1 mg/ml), 1.8 μM (0.25 mg/ml), and 0.45 μM (0.0625 mg/ml) concentrations at injection, respectively (molecular weight of each peak represented by intersecting lines; concentrations and molecular weights also listed in table).

Figure S5, related to Figure 5 and Extended Experimental Procedures: Fluorescence microscopy experiments reveal PlxnA *cis*-intermolecular interaction on COS-7 cell surface. (A) Single molecule localization microscopy-based cluster analyses reveal that PlxnA2 does not form large cell-surface clusters. Distribution of distances between single PlxnA2 molecules (trace colors as indicated) compared to the distribution of distances between a set of homogenous, randomly distributed points of the same mean density (black trace; I). Sample size, N = 3. A simulated set of homogenous, randomly distributed points following the Poisson process with the same density as the detected single molecules is shown in II. Representative wide-field and single molecule localization microscopy images showing the distribution of $PlxnA2_{FL}$ and $PlxnA2_{FL}$ F690N/692S before and after Sema6A binding are shown in III. The white boxes in the wide-field images represent the regions of interest (ROI; upper panels), from which single receptor molecules

located by our method are indicated in white (bottom panels). Scale bar, 5 µM. (B) FRET-FLIM analysis. When PlxnA2 ectodomain-transmembrane segments fused only to the FRET donor mTFP1 (cyan) interact, no FRET can occur (I; upper panel). The fluorescence lifetime decay of mTFP1 can be fitted to a mono-exponential model (green trace; normalized photon counts). When both PlxnA2 molecules fused to mTPF1 and molecules fused to mVenus (yellow) interact via the sema–PSI2-IPT2 interface, FRET occurs (I; lower panel). In this case the accelerated fluorescence lifetime decay of mTFP1 can be fitted to a bi-exponential model (orange trace). The extent of FRET is independent of donor/acceptor fluorescence intensity ratio as shown by the random distribution of the PlxnA2-mTFP1/PlxnA2-mVenus intensity ratio (y-axis) against the average f_D ($\overline{f_D}$) (x-axis) in II (from > 50 randomly sampled cells).

Figure S6, related to Figure 6: Growth cone morphology matrix for the quantification of DG granule cell growth cone collapse assays. Growth cones with different morphologies ranging from 1 (uncollapsed, fan-like morphology) to 10 (fully collapsed morphology) were selected from our experiments. This generates a matrix which was used to grade growth cones after the treatment of vehicles or purified PlxnA1$_{4\text{-}5}$ proteins (wild type PlxnA1$_{4\text{-}5}$ and mutant PlxnA1$_{4\text{-}5}$ F693N/E695S). Each row represents a range from 1 to 10 with different examples. All growth cones were visualized by phalloidin staining.

SUPPLEMENTAL TABLE

Table S1, related to Figure 1, Figure S1 and Extended Experimental Procedures: Data collection and refinement statistics.

	$PlxnA1_{1\text{-}10}$	$PlxnA1_{1\text{-}10}$	$PlxnA1_{1\text{-}10}$	$PlxnA2_{1\text{-}10}$	$PlxnA4_{1\text{-}10}$
**Data collection**					
Space group	$P4_32_12$	$P2_1$	$P4_32_12$	$P3_221$	$P4_1$
Cell dimensions					
a, *b*, *c* (Å)	195, 195, 176	140, 196, 145	198, 198, 229	238, 238, 642	221, 221, 66
α, β, γ (°)	90, 90, 90	90, 94.6, 90	90, 90, 90	90, 90, 120	90, 90, 90
Resolution (Å)	87-4.0 (4.24-4.00)[a]	58-6.0 (6.57-6.00)[a]	112-6.0 (6.71-6.00)[a]	63-10 (10.5-10.0)[a]	62-7.5 (8.39-7.50)[a]
R_{merge} (%)	24.1 (104)	7.0 (72.9)	7.1 (124)	18.7 (88.2)	20.7 (121)
$I/\sigma I$	5.2 (1.5)	6.8 (1.5)	9.7 (1.6)	5.1 (1.5)	12.2 (2.8)
$CC_{1/2}$	0.95 (0.52)	1.00 (0.50)	1.00 (0.49)	0.99 (0.70)	1.00 (0.59)
Completeness (%)	99.1 (99.8)	99.7 (99.9)	98.8 (98.9)	93.9 (96.8)	99.8 (100)
Redundancy	5.1 (5.1)	3.2 (3.3)	6.6 (6.9)	3.3 (3.6)	6.9 (7.1)
**Refinement**					
Resolution (Å)	87-4.0	58-6.0	112-6.0	63-10.0	55-7.5
Number of reflections	28954	19391	11641	11079	4253
R_{work}/R_{free} (%)	25.7 / 30.8	27.4 / 30.0	30.5 / 31.6	33.5 / 37.0	35.3 / 37.1
Number of atoms	9692	19191	9546	28787	9134
Protein	9692	19191	9546	28787	9134
Ligand/ion	0	0	0	0	0
Water	0	0	0	0	0
B factors (Å^2)					
Protein	134	273	283	237	250
Ligand/ion	–	–	–	–	–
Water	–	–	–	–	–
R.m.s. deviations					
Bond lengths (Å)	0.006	0.006	0.006	0.019	0.019
Bond angles (°)	1.2	1.3	1.2	1.7	2.4

	$PlxnA4_{1-10}$	$PlxnA4_{1-10}$	$PlxnA4_{1-10}$	$PlxnA2_{4-5}$	$PlxnA1_{7-10}$
**Data collection**					
Space group	$P2_1$	$P4_32_12$	$P4_322$	$C2$	$C222_1$
Cell dimensions					
a, *b*, *c* (Å)	142, 241, 144	272, 272, 251	189, 189, 253	107.7, 44.6, 33.0	75.9, 82.5, 125.6
α, β, γ (°)	90, 99.8, 90	90, 90, 90	90, 90, 90	90, 104.7, 90	90, 90, 90
Resolution (Å)	48-8.0 (8.95-8.00)[a]	72-8.0 (8.94-8.00)[a]	71-8.5 (9.50-8.50)[a]	52.1-1.36 (1.40-1.36)[a]	62.8-2.2 (2.27-2.20)[a]
R_{merge} (%)	15.8 (76.4)	13.0 (89.4)	33.0 (117)	3.8 (47.6)	12.0 (82.5)
$I/\sigma I$	9.0 (2.9)	14.8 (1.3)	4.2 (1.7)	17.0 (2.3)	9.2 (1.9)
$CC_{1/2}$	1.00 (0.63)	1.00 (0.56)	0.98 (0.67)	1.00 (0.70)	1.00 (0.57)
Completeness (%)	99.5 (100)	99.5 (100)	99.7 (100)	85.6 (41.0)	100 (99.8)
Redundancy	4.1 (4.3)	3.7 (3.9)	5.8 (6.1)	3.7 (3.4)	8.1 (5.0)
**Refinement**					
Resolution (Å)	48-8.0	72-8.0	70-8.5	52.1-1.36	62.8-2.2
Number of reflections	10061	10298	4374	27858	20361
R_{work}/R_{free} (%)	34.9 / 34.9	37.3 / 39.5	40.1 / 41.2	14.1 / 19.6	23.0 / 27.0
Number of atoms	15030	7189	7189	1334	2821
Protein	15030	7189	7189	1228	2680
Ligand/ion	0	0	0	3	11
Water	0	0	0	103	130
B factors ($Å^2$)					
Protein	264	251	163	16.3	50.4
Ligand/ion	–	–	–	24.9	77.2
Water	–	–	–	29.6	43.6
R.m.s. deviations					
Bond lengths (Å)	0.019	0.019	0.019	0.020	0.002
Bond angles (°)	2.5	2.5	2.5	2.00	0.53

[a]Highest resolution shell is shown in parenthesis.

EXTENDED EXPERIMENTAL PROCEDURES

Cloning and Protein Production

Constructs of mouse versions of PlxnA1$_{1\text{-}10}$, PlxnA1$_{7\text{-}10}$, PlxnA2$_{1\text{-}10}$, PlxnA2$_{1\text{-}2}$, PlxnA2$_{4\text{-}5}$ and PlxnA4$_{1\text{-}10}$ (residues 37-1236, 861-1236, 35-1231, 35-561, 655-804, 36-1229, respectively) were produced using the pHLsec vector (Aricescu et al., 2006) with a C-terminal hexahistidine tag and expressed in HEK 293S (Reeves et al., 2002) and HEK 293T cells. Several interface mutants, PlxnA1$_{1\text{-}10}$ F145N/L147S, PlxnA1$_{1\text{-}10}$ F693N/E695S, PlxnA2$_{4\text{-}5}$ F690N/E692S, PlxnA2$_{1\text{-}10}$ F690N/E692S-mTFP1, PlxnA2$_{1\text{-}10}$ F690N/E692S-mVenus, PlxnA2$_{fl}$-mVenus and PlxnA2$_{fl}$ F690N/E692S-mVenus, were produced via 2-step site directed mutagenesis to introduce N-linked glycosylation sites in the sema–PSI2-IPT2 interface. Ligand proteins used in SPR experiments were cloned into the pHLsec expression vector with a C-terminal hexahistidine tag or a C-terminal BirA recognition sequence for biotinylation (O'Callaghan et al., 1999). Mouse Sema6A (residues 19-571) was cloned into the pHLsec vector resulting in a protein construct with a C-terminal Fc tag that was covalently dimerized. All protein samples were purified from buffer-exchanged medium via dialysis and concentration, followed by immobilized nickel or cobalt metal-affinity chromatography and subsequent size-exclusion chromatography using a prepacked HiLoad 16/600 Superdex 200 prep grade column from GE Healthcare Life Sciences.

Crystallization and Data Collection

Crystallization experiments were conducted by mixing 100 nl (or 200 nl for PlxnA2$_{1\text{-}10}$) of protein solution with 100 nl reservoir solution using a Cartesian Technologies pipetting robot (Walter et al., 2005). Crystallization plates were maintained at 20.5 °C in a TAP Homebase storage vault and imaged with a Veeco visualization system (Mayo et al., 2005). PlxnA1$_{1\text{-}10}$ was concentrated to 8.2 mg/ml in 20 mM HEPES, pH

7.5 and 150 mM sodium chloride and subsequently crystallized in three different crystal forms. One crystal form, with space group $P4_32_12$ grown in 6% (w/v) PEG 4k and 5 mM tricine, pH 8.5 (or 5 mM TRIS, pH 8.5), diffracted to 6.0 Å resolution. Dehydration of these crystals in the plate by increasing the PEG 4k concentration in the mother liquor in three steps to 25% over a time span of 48 hours produced a second crystal form with a 53 Å shorter *c*-axis, that diffracted to 4.0 Å resolution. A third crystal form with space group $P2_1$, diffracting to 6.0 Å resolution, was grown in 15% (v/v) propanol, 20 mM magnesium chloride and 50 mM MES, pH 6.0. For PlxnA2$_{1\text{-}10}$ we obtained one crystal form. PlxnA2$_{1\text{-}10}$ was concentrated to 6.6 mg/ml in 20 mM HEPES, pH 7.5 and 150 mM sodium chloride and subsequently deglycosylated with endoglycosidase F1. Crystals of PlxnA2$_{1\text{-}10}$ with space group $P3_221$ were produced in 0.68 M potassium/sodium tartrate and 85 mM HEPES, pH 7.5 and diffracted to 10 Å resolution. PlxnA4$_{1\text{-}10}$ was crystallized in four different crystal forms. PlxnA4$_{1\text{-}10}$ concentrated to 3.8 mg/ml in 20 mM HEPES, pH 7.5, 150 mM sodium chloride, 100 mM dimethylbenzylammonium propane sulfonate (Non-Detergent Sulfobetaine 256; NDSB-256) and deglycosylated with endoglycosidase F1, crystallized in space group $P4_1$ in 29% (v/v) glycerol, 4.3% (w/v) PEG 8k and 53 mM TRIS hydrochloride, pH 8.5 and diffracted to 7.5 Å resolution. PlxnA4$_{1\text{-}10}$ concentrated to 4.2 mg/ml in 10 mM citrate, pH 5.1 yielded a second and third crystal form. Crystals with space group $P2_1$ were obtained in 0.5 M sodium succinate, 100 mM bis-TRIS propane, pH 7.0 and diffracted to 8.0 Å resolution and crystals with space group $P4_32_12$ were grown in 0.8 M ammonium sulfate, 0.4 M dimethyl (2-hydroxyethyl) ammonium propane sulfonate (NDSB-211), 100 mM TRIS, pH 8.0 and diffracted to 8 Å resolution. PlxnA4$_{1\text{-}10}$ concentrated to 5.8 mg/ml in 20 mM HEPES, pH 7.5, 150 mM sodium chloride and 100 mM NDSB-256 crystallized in a fourth space group, $P4_322$, in 1.5 lithium sulfate, 100 mM TRIS, pH 8.5 and diffracted to 8.5

Å resolution. $PlxnA2_{4\text{-}5}$ was concentrated to 6.5 mg/ml in 10 mM HEPES pH 7.5, 150 mM sodium chloride, and crystallized in space group C2, in 25% (w/v) polyethylene glycol 3,350 and 100 mM BIS-TRIS pH 5.5. Lysine methylation of $PlxnA1_{7\text{-}10}$ using dimethylamine-borane complex and formaldehyde was performed as described earlier (Walter et al., 2006). $PlxnA1_{7\text{-}10}$ was concentrated to 7.5 mg/ml in 10 mM TRIS, pH 8.0, 150 mM sodium chloride, and crystallized in space group $C222_1$, in 25% (w/v) polyethylene glycol 3,350, 100mM BIS-TRIS pH 5.5 and 200mM potassium sodium tartrate. Before diffraction data collection crystals were soaked in mother liquor supplemented with 12.5% (v/v) 2,3-butanediol for $PlxnA1_{1\text{-}10}$ crystals, 25% (v/v) glycerol for $PlxnA2_{1\text{-}10}$, $PlxnA4_{1\text{-}10}$ and $PlxnA2_{4\text{-}5}$ crystals, or 25% ethylene glycol for $PlxnA1_{7\text{-}10}$ crystals (for $PlxnA4_{1\text{-}10}$ crystals in space group $P4_1$ nothing extra was added). Data were collected at 100 K at Diamond beamlines I02, I03, I04, I04-1 and I24 and European Synchrotron Radiation Facility beamline ID23-2. Diffraction data were integrated, scaled and merged with MOSFLM (Leslie, 2006) and SCALA (Evans, 2011) or AIMLESS in CCP4 (CCP4, 1994).

Structure solution and refinement

The structure of $PlxnA1_{1\text{-}10}$ at 4.0 Å resolution was initially solved by molecular replacement in PHASER (McCoy, 2007; McCoy et al., 2007) using the structure of $PlxnA2_{1\text{-}4}$ (Janssen et al., 2010) (PDB: 3OKT) (54% sequence identity with $PlxnA1_{1\text{-}4}$). After solvent flattening in PARROT (Cowtan, 2010) this solution revealed clear electron density for PlxnA1 domains IPT2, PSI3, IPT3, IPT4 and IPT5 that were not included in the search model. To further complete the structure of $PlxnA1_{1\text{-}10}$ we generated homology models of these domains with MODELLER (Fiser and Šali, 2003) using the following template PDB–PlxnA1 domain combinations; PDB: 3OKY (Janssen et al., 2010) for IPT2, PDB: 3NVN (Liu et al., 2010) for PSI3, PDB: 2UZY (Niemann et al., 2007) for IPT3 and IPT4, and PDB: 2CXK (unpublished results) for

IPT5, with 16%, 29%, 29%, 29% and 20% sequence identity, respectively. These models were placed by molecular replacement in PHASER and the resulting structure was further optimized by manual rebuilding in COOT (Emsley and Cowtan, 2004) and refinement in REFMAC (Murshudov et al., 2011) using jelly-body restraints (Nicholls et al., 2012). However, the low 4.0 Å resolution of the data prevented unambiguous assignment of the sequence register for those parts of the structure for which no high quality homology model was available, for example for domains IPT2 and IPT5. Furthermore, the weaker electron density of domain IPT6 prevented reliable building of this domain. We therefore sought to determine much higher resolution crystal structures for these segments in isolation. We collected 1.36 Å resolution data from $PlxnA2_{4\text{-}5}$ crystals and 2.2 Å resolution data from $PlxnA1_{7\text{-}10}$ crystals. The structures of $PlxnA2_{4\text{-}5}$ and $PlxnA1_{7\text{-}10}$ were solved by molecular replacement in PHASER using the corresponding partially refined domains PSI2-IPT2 and IPT3-IPT4-IPT5, respectively, from the $PlxnA1_{1\text{-}10}$ structure. Both solutions were completed by model building in COOT and refinement in REFMAC (for $PlxnA2_{4\text{-}5}$) and BUSTER (Smart et al., 2012) and PHENIX (Adams et al., 2002) (for $PlxnA1_{7\text{-}10}$). For domain IPT6 of $PlxnA1_{7\text{-}10}$ our maps showed only fragmentary electron density for two loops (residues 1153-1163 and 1210-1217) and the C-terminal residues (1228-1236), these regions were therefore not modelled. We used these refined structures to replace domains IPT2, IPT3, IPT4, IPT5 and segments of domain IPT6 in the $PlxnA1_{1\text{-}10}$ structure which was then completed by model building in COOT and refinement in REFMAC using jelly-body restraints.

All other PlxnA full extracellular segment structures were solved using the refined 4.0 Å resolution $PlxnA1_{1\text{-}10}$ structure and, for $PlxnA2_{1\text{-}10}$, also the $PlxnA2_{1\text{-}4}$ structure (Janssen et al., 2010), PDB: 3OKT. Homology models for PlxnA2 and PlxnA4 were calculated with MODELLER using the $PlxnA1_{1\text{-}10}$ structure as the template for PlxnA2

domains 5 to 10 (IPT2, PSI3, IPT3, IPT4, IPT5 and IPT6) (53% sequence identity) and for all ten PlxnA4 domains (54% sequence identity). The other PlxnA1$_{1\text{-}10}$, PlxnA2$_{1\text{-}10}$ and PlxnA4$_{1\text{-}10}$ crystal forms were solved by molecular replacement in PHASER using individual domains of the PlxnA1$_{1\text{-}10}$ structure, of the PlxnA2$_{1\text{-}4}$ structure and PlxnA2$_{5\text{-}10}$ homology model, and of the PlxnA4$_{1\text{-}10}$ homology model, respectively, as search models. Domains were omitted from the models in cases where the electron density was too weak for interpretation due to flexibility (see Figure S1 for the entire set of structures and domains present). All solutions were refined by rigid-body refinement in PHENIX with each domain as a rigid group and a single *B* factor per domain, thus preventing any overfitting. Refinement statistics for all structures are given in Table S1. Electrostatic charge distribution was calculated with PDB2PQR (Dolinsky et al., 2004) and APBS (Baker et al., 2001), alignments were generated with Clustal Omega (Sievers et al., 2011), residue conservation was calculated with The Consurf Server (Glaser et al., 2003) or MODELLER and buried surface areas were calculated with PISA (Krissinel and Henrick, 2007). Figures were produced with The PyMOL Molecular Graphics System (Schrödinger, LLC).

Negative stain electron microscopy images analysis

For the preparation of carbon-coated grids and electron microscope set up, we followed the previously described protocols for negative stain EM (Booth et al., 2011). In brief, 2.5 µl of freshly gel-filtrated PlxnA1$_{1\text{-}10}$ at a concentration of 1-5 μg/ml in 10 mM HEPES, pH 7.5 and 150 mM sodium chloride was adsorbed to a glow-discharged carbon-coated copper grid, washed with two drops of deionized water, and stained with two drops of freshly prepared 0.75% uranyl formate. From 355 electron micrographs, a total of 12,645 particles were manually selected from 355 images using EMAN2 (Tang et al., 2007) and framed into boxes with a size of 298 Å × 298 Å. The particle images were normalized, filtered, centered, iteratively aligned

and classified without any starting reference using IMAGIC (van Heel et al., 1996) to generate 60 class averages. The structural models based on the 4 Å crystal structure of $PlxnA1_{1-10}$ were generated manually using The PyMOL Molecular Graphics System (Schrödinger, LLC). The two-dimensional projections of our models were then subjected to automated correlation analysis with the class averages in which all the class averages were compared to all projections of all models. Thirteen models were tested and of these seven were found to be sufficient to represent the experimental class averages. Two-dimensional projections of the crystal structure and models were generated using SPIDER and WEB (Frank et al., 1996) and WellMAP (Flanagan et al., 2010) software and the correlations of the class averages were performed using scripts operating through SPIDER.

Single molecule localization microscopy-based cluster analysis

COS-7 cells were transiently transfected with PlxnA2 full-length constructs conjugated to mVenus at the C-termini via X-treme GENE HP Transfection Reagent and incubated at 37 °C, 5% CO_2 for 24 hours before fixing with 4% paraformaldehyde on ice. The fixed cells were mounted onto the glass coverslip using Prolong® Gold antifate reagent from Life Technologies™. We used a single molecule localization microscopy technique based on the stochastic switching of standard fluorescent proteins (Lemmer et al., 2008), similar to the principle of (F)PALM (Betzig et al., 2006) and STORM (Rust et al., 2006) which rely on special photo-activatable or photo-switchable fluorophores. To perform localization microscopy, we used an OMX (optical microscope experimental, V2, API) microscopic system modified to enable single molecule localization microscopy using conventional fluorescent proteins according to the method described previously (Lemmer et al., 2008). The intensity of the 488 nm laser was adjusted to ~14 kW/cm^2 in the object plane for localization microscopy imaging of the mVenus conjugated samples. Single molecule positions

were determined using an algorithm based on a maximum-likelihood method optimized for localization microscopy data (Grull et al., 2011) and adapted to the OMX hardware configuration. Distance and cluster analyses of the single molecule data were performed with algorithms described earlier (Kaufmann et al., 2011; Seiradake et al., 2013).

Imaging PlxnA2 interactions in live COS-7 cells using FRET-FLIM

COS 7 cells transiently transfected with PlxnA2 constructs and the mTFP-mVenus Tandem together with GeneJuice transfection reagent (Novagen) were grown to 60–70% confluency on glass–bottom 35 mm Petri dishes (Mattek) in Dulbecco's modified eagle medium with 10% fetal bovine serum. Before imaging, medium was changed to 0.5% fetal bovine serum PBS imaging buffer without phenol red and the observation chamber was mounted onto a SP8–SMD Leica microscope (Leica Microsystems, Germany). A suitable image field focusing on the surface of a robust COS-7 cell was chosen with a 63×/1.4 NA oil immersion objective. For each FLIM image, mTFP1 was excited by a 440 nm pulsed laser (Picoquant GmbH, Germany) at 40 MHz using single photon counting electronics (PicoHarp 300). Emitted single photons from mTFP1 passing through a 460–500 nm filter were subsequently detected by a HyD detector (Leica Microsystems). The acquired fluorescence decay, $i(t)$, of each pixel in one whole cell were deconvoluted with the instrument response function (IRF) and fitted by a Marquandt nonlinear least–square algorithm (Marquardt, 1963) with mono- or bi-exponential theoretical models using Symphotime software from Picoquant GmbH (detailed in Padilla-Parra et al., 2008; 2015). In brief, the mean fluorescence lifetime $\langle\tau\rangle$ of a fluorescence decay $i(t)$, is defined by the following equation:

$$\langle \tau \rangle = \int t \times i(t) dt \Big/ \int i(t) dt \qquad Eq.1$$

The fluorescence lifetime obtained from a mono-exponential decay model of cells expressing only PlxnA2 molecules fused to the donor mTFP1 was first assigned. This lifetime was then fixed as the non-interacting fraction in a bi-exponential model obtained from the fluorescence lifetime decay profile of cells expressing both PlxnA2 fused to mTFP1 and mVenus (Padilla-Parra et al., 2008) using the following equation:

$$i(t) = (1 - f_D)e^{-t/\tau_D} + f_D e^{-t/\tau_F} \qquad Eq.2$$

where f_D stands for the fraction of interacting donor, τ_D is the fixed donor lifetime and τ_F is the discrete FRET lifetime. The value of f_D per pixel was calculated from the mean fluorescence lifetime ($\langle \tau \rangle$) using 1200 channels of fluorescence decay (Padilla-Parra et al., 2008; 2015). One should note that we were only capable of detecting ~1/4 of the real interaction (0.5 because of our labelling strategy and ~0.5 given the dynamic range of the FRET couple (Galperin et al., 2004; Padilla-Parra et al., 2009). This factor together with the spectral heterogeneity of the fluorophores and the temporal resolution of our technique determined that the f_D represents the minimal fraction of the actual interaction (Padilla-Parra et al., 2008; 2015; Yasuda, 2006).

DG growth cone assay

DG granule cell cultures were prepared as described previously (Van Battum et al., 2014). In brief, hippocampi were dissected from P6-8 C57bl6J mice and cut into 350 µm slices using a tissue chopper (McIllwain). The DG was dissected from these slices and collected in 1x Krebs medium (0.7% NaCl, 0.04% KCl, 0.02% KH_2PO_4, 0.2%$NaHCO_3$, 0.25% glucose and 0.001% phenol red). Cells were dissociated after

incubation in 1x Trypsin in Krebs medium for 15 min at 37°C. The reaction was stopped by adding 20 mg/ml soybean trypsin inhibitor followed by trituration using a firepolished Pasteur pipette in Krebs medium containing 2 mg soybean trypsin inhibitor and 20 µg/ml DNAseI. Dissociated DG granule cells were resuspended in Neurobasal medium supplemented with B-27, L-glutamine, penicillin-streptomycin and β-mercaptoethanol and plated on coverslips coated with poly-D-lysine (20 mg/ml) and laminin (40 µg/ml) in 24 well plate and incubated in a humidified incubator at 37°C and 5% CO_2. Proteins were filtered using 0.45 µm filter before incubation with cells. On day *in vitro* (DIV)1, cells were treated with vehicle, or wild type or mutant $PlxnA1_{4-5}$ recombinant proteins (3 mg/ml) for 30 min at 37°C, fixed in 4% PFA and sucrose for 20 min at room temperature, and immunostained with anti-βIII-tubulin antibodies and phalloidin. Images were acquired using a 100× objective on an AxioScopeA1 (Zeiss) microscope and analyzed using a growth cone morphology matrix (Figure S6) in an observer-blind manner. The matrix was composed of 40 growth cones from different experiments that represent the full range of morphologies observed, ranging from a normal fan-shaped morphology (1) to full blown collapse (10). This strategy was applied as it allows the detection of subtle changes in growth cone morphology rather than just a comparison of collapse versus non-collapse.

SUPPLEMENTAL REFERENCES

Adams, P.D., Grosse-Kunstleve, R.W., Hung, L.W., Ioerger, T.R., McCoy, A.J., Moriarty, N.W., Read, R.J., Sacchettini, J.C., Sauter, N.K., and Terwilliger, T.C. (2002). PHENIX: building new software for automated crystallographic structure determination. Acta Crystallogr. D Biol. Crystallogr. *58*, 1948–1954.

Aricescu, A.R., Lu, W., and Jones, E.Y. (2006). A time- and cost-efficient system for high-level protein production in mammalian cells. Acta Crystallogr. D Biol. Crystallogr. *62*, 1243–1250.

Baker, N.A., Sept, D., Joseph, S., Holst, M.J., and McCammon, J.A. (2001). Electrostatics of nanosystems: Application to microtubules and the ribosome. Proc. Natl. Acad. Sci. U.S.A. *98*, 10037–10041.

Betzig, E., Patterson, G.H., Sougrat, R., Lindwasser, O.W., Olenych, S., Bonifacino, J.S., Davidson, M.W., Lippincott-Schwartz, J., and Hess, H.F. (2006). Imaging Intracellular Fluorescent Proteins at Nanometer Resolution. Science *313*, 1642–1645.

Booth, D.S., Avila-Sakar, A., and Cheng, Y. (2011). Visualizing proteins and macromolecular complexes by negative stain em: from grid preparation to image acquisition. J. Vis. Exp. http://dx.doi.org/10.3791/3227.

Cowtan, K. (2010). Recent developments in classical density modification. Acta Crystallogr. D Biol. Crystallogr. 66(4), 470-478.

Dolinsky, T.J., Nielsen, J.E., McCammon, J.A., and Baker, N.A. (2004). PDB2PQR: an automated pipeline for the setup of Poisson-Boltzmann electrostatics calculations. Nucleic Acids Res. *32*, W665–W667.

Emsley, P., and Cowtan, K. (2004). Coot: model-building tools for molecular graphics. Acta Crystallogr. D Biol. Crystallogr. *60*, 2126–2132.

Evans, P.R. (2011). An introduction to data reduction: space-group determination, scaling and intensity statistics. Acta Crystallogr. D Biol. Crystallogr. *67*, 282–292.

Fiser, A., and Šali, A. (2003). Modeller: Generation and Refinement of Homology-Based Protein Structure Models. Methods Enzymol. *374*, 461–491.

Flanagan, J.F., Namy, O., Brierley, I., and Gilbert, R.J.C. (2010). Direct observation of distinct A/P hybrid-state tRNAs in translocating ribosomes. Structure *18*, 257–264.

Frank, J., Radermacher, M., Penczek, P., Zhu, J., Li, Y., Ladjadj, M., and Leith, A. (1996). SPIDER and WEB: Processing and Visualization of Images in 3D Electron Microscopy and Related Fields. J. Struct. Biol. *116*, 190–199.

Galperin, E., Verkhusha, V.V., and Sorkin, A. (2004). Three-chromophore FRET microscopy to analyze multiprotein interactions in living cells. Nat. Meth. *1*, 209–217.

Glaser, F., Pupko, T., Paz, I., Bell, R.E., Bechor-Shental, D., Martz, E., and Ben-Tal, N. (2003). ConSurf: Identification of Functional Regions in Proteins by Surface-Mapping of Phylogenetic Information. Bioinformatics *19*, 163–164.

Gruell, F., Kirchgessner, M., Kaufmann, R., Hausmann, M. & Kebschull, U. Accelerating image analysis for localization microscopy with FPGAs. (2011) International Conference on Field Programmable Logic and Applications 1–5 (IEEE).

Janssen, B.J.C., Robinson, R.A., Pérez-Brangulí, F., Bell, C.H., Mitchell, K.J., Siebold, C., and Jones, E.Y. (2010). Structural basis of semaphorin-plexin signalling. Nature *467*, 1118–1122.

Kaufmann, R., Müller, P., Hildenbrand, G., Hausmann, M., and Cremer, C. (2011). Analysis of Her2/neu membrane protein clusters in different types of breast cancer cells using localization microscopy. J. Microsc. *242*, 46–54.

Krissinel, E., and Henrick, K. (2007). Inference of Macromolecular Assemblies from Crystalline State. J. Mol. Biol. *372*, 774–797.

Lemmer, P., Gunkel, M., Baddeley, D., Kaufmann, R., Urich, A., Weiland, Y., Reymann, J., Müller, P., Hausmann, M., and Cremer, C. (2008). SPDM: light microscopy with single-molecule resolution at the nanoscale. Appl. Phys. B *93*, 1–12.

Leslie, A.G.W. (2006). The integration of macromolecular diffraction data. Acta Crystallogr. D Biol. Crystallogr. *62*, 48–57.

Liu, H., Juo, Z.S., Shim, A.H.-R., Focia, P.J., Chen, X., Garcia, K.C., and He, X. (2010). Structural basis of semaphorin-plexin recognition and viral mimicry from Sema7A and A39R complexes with PlexinC1. Cell *142*, 749–761.

Marquardt, D.W. (1963). An Algorithm for Least-Squares Estimation of Nonlinear Parameters. SIAM J. Appl. Math. *11*, 431–441.

Mayo, C.J., Diprose, J.M., Walter, T.S., Berry, I.M., Wilson, J., Owens, R.J., Jones, E.Y., Harlos, K., Stuart, D.I., and Esnouf, R.M. (2005). Benefits of Automated Crystallization Plate Tracking, Imaging, and Analysis. Structure *13*, 175–182.

McCoy, A.J. (2007). Solving structures of protein complexes by molecular replacement with Phaser. Acta Crystallogr. D Biol. Crystallogr. *63*, 32–41.

McCoy, A.J., Grosse-Kunstleve, R.W., Adams, P.D., Winn, M.D., Storoni, L.C., and Read, R.J. (2007). Phasercrystallographic software. J. Appl. Crystallogr. *40*, 658–674.

Murshudov, G.N., Skubák, P., Lebedev, A.A., Pannu, N.S., Steiner, R.A., Nicholls, R.A., Winn, M.D., Long, F., and Vagin, A.A. (2011). REFMAC5 for the refinement of macromolecular crystal structures. Acta Crystallogr. D Biol. Crystallogr. *67*, 355–367.

Nicholls, R.A., Long, F., and Murshudov, G.N. (2012). Low-resolution refinement tools in REFMAC5. Acta Crystallogr. D Biol. Crystallogr. *68*, 404–417.

Niemann, H.H., Jäger, V., Butler, P.J.G., van den Heuvel, J., Schmidt, S., Ferraris, D., Gherardi, E., and Heinz, D.W. (2007). Structure of the Human Receptor Tyrosine Kinase Met in Complex with the Listeria Invasion Protein InlB. Cell *130*, 235–246.

O'Callaghan, C.A., Byford, M.F., Wyer, J.R., Willcox, B.E., Jakobsen, B.K., McMichael, A.J., and Bell, J.I. (1999). BirA Enzyme: Production and Application in the Study of Membrane Receptor–Ligand Interactions by Site-Specific Biotinylation. Anal. Biochem. *266*, 9–15.

Padilla-(, S., Audugé, N., Coppey-Moisan, M., and Tramier, M. (2008). Quantitative FRET Analysis by Fast Acquisition Time Domain FLIM at High Spatial Resolution in Living Cells. Biophys. J. *95*, 2976–2988.

Padilla-Parra, S., Audugé, N., Lalucque, H., Mevel, J.-C., Coppey-Moisan, M., and Tramier, M. (2009) Quantitative Comparison of Different Fluorescent Protein Couples for Fast FRET-FLIM Acquisition. Biophys. J. *97*, 2368–2376.

Padilla-Parra, S., Audugé, N., Tramier, M., and Coppey-Moisan, M. (2015). Time-domain fluorescence lifetime imaging microscopy: a quantitative method to follow transient protein-protein interactions in living cells. Cold Spring Harb. Protoc. *6*, 508–521.

Reeves, P.J., Callewaert, N., Contreras, R., and Khorana, H.G. (2002). Structure and function in rhodopsin: High-level expression of rhodopsin with restricted and homogeneous N-glycosylation by a tetracycline-inducible N-acetylglucosaminyltransferase I-negative HEK293S stable mammalian cell line. Proc. Natl. Acad. Sci. U.S.A. *99*, 13419–13424.

Rust, M.J., Bates, M., and Zhuang, X. (2006). Sub-diffraction-limit imaging by stochastic optical reconstruction microscopy (STORM). Nat. Meth. *3*, 793–796.

Seiradake, E., Schaupp, A., del Toro Ruiz, D., Kaufmann, R., Mitakidis, N., Harlos, K., Aricescu, A.R., Klein, R., and Jones, E.Y. (2013). Structurally encoded intraclass differences in EphA clusters drive distinct cell responses. Nat. Struct. Mol. Biol. *20*, 958–964.

Sievers, F., Wilm, A., Dineen, D., Gibson, T.J., Karplus, K., Li, W., Lopez, R., McWilliam, H., Remmert, M., Soding, J., et al. (2011). Fast, scalable generation of high-quality protein multiple sequence alignments using Clustal Omega. Mol. Syst. Biol. *7*, 539–539.

Smart, O.S., Womack, T.O., Flensburg, C., Keller, P., Paciorek, W., Sharff, A., Vonrhein, C., and Bricogne, G. (2012). Exploiting structure similarity in refinement: automated NCS and target-structure restraints in BUSTER. Acta Crystallogr. D Biol. Crystallogr. *68*, 368–380.

Tang, G., Peng, L., Baldwin, P.R., Mann, D.S., Jiang, W., Rees, I., and Ludtke, S.J. (2007). EMAN2: An extensible image processing suite for electron microscopy. J. Struct. Biol. *157*, 38–46.

van Heel, M., Harauz, G., Orlova, E.V., Schmidt, R., and Schatz, M. (1996). A New Generation of the IMAGIC Image Processing System. J. Struct. Biol., *116*, 17–24.

Walter, T.S., Diprose, J.M., Mayo, C.J., Siebold, C., Pickford, M.G., Carter, L., Sutton, G.C., Berrow, N.S., Brown, J., Berry, I.M., et al. (2005). A procedure for setting up high-throughput nanolitre crystallization experiments. Crystallization workflow for initial screening, automated storage, imaging and optimization. Acta Crystallogr. D Biol. Crystallogr. *61*, 651–657.

Walter, T.S., Meier, C., Assenberg, R., Au, K.-F., Ren, J., Verma, A., Nettleship, J.E., Owens, R.J., Stuart, D.I., and Grimes, J.M. (2006) Lysine Methylation as a Routine

Rescue Strategy for Protein Crystallization. Structure *14*, 1617–1622.

Yasuda, R. (2006). Imaging spatiotemporal dynamics of neuronal signaling using fluorescence resonance energy transfer and fluorescence lifetime imaging microscopy. Curr. Opin. Neurobiol. *16*, 551–561.

Collaborative Computational Project Number 4 (1994). The CCP4 suite: programs for protein crystallography. Acta Cryst. *D50,* 760-763.
